# Local-to-global signal transduction at the core of a Mn^2+^ sensing riboswitch

**DOI:** 10.1038/s41467-019-12230-5

**Published:** 2019-09-20

**Authors:** Krishna C. Suddala, Ian R. Price, Shiba S. Dandpat, Michal Janeček, Petra Kührová, Jiří Šponer, Pavel Banáš, Ailong Ke, Nils G. Walter

**Affiliations:** 1 Single Molecule Analysis Group and Center for RNA Biomedicine, Department of Chemistry, University of Michigan, Ann Arbor, MI 48109 USA; 2 Department of Molecular Biology and Genetics, Cornell University, Ithaca, NY 14850 USA; 3 Institute of Biophysics of the Czech Academy of Sciences, Kralovopolská 135, Brno, 612 65 Czech Republic; 4 Department of Physical Chemistry, Faculty of Science, Palacký University, tř. 17 listopadu 12, Olomouc, 771 46 Czech Republic; 5 Regional Centre of Advanced Technologies and Materials, Faculty of Science, Palacký University, tř. 17 listopadu 12, Olomouc, 771 46 Czech Republic

**Keywords:** Molecular conformation, Single-molecule biophysics, Riboswitches

## Abstract

The widespread Mn^2+^-sensing *yybP-ykoY* riboswitch controls the expression of bacterial Mn^2+^ homeostasis genes. Here, we first determine the crystal structure of the ligand-bound *yybP-ykoY* riboswitch aptamer from *Xanthomonas oryzae* at 2.96 Å resolution, revealing two conformations with docked four-way junction (4WJ) and incompletely coordinated metal ions. In >100 µs of MD simulations, we observe that loss of divalents from the core triggers local structural perturbations in the adjacent docking interface, laying the foundation for signal transduction to the regulatory switch helix. Using single-molecule FRET, we unveil a previously unobserved extended 4WJ conformation that samples transient docked states in the presence of Mg^2+^. Only upon adding sub-millimolar Mn^2+^, however, can the 4WJ dock stably, a feature lost upon mutation of an adenosine contacting Mn^2+^ in the core. These observations illuminate how subtly differing ligand preferences of competing metal ions become amplified by the coupling of local with global RNA dynamics.

## Abstract (web summary)

Riboswitches bind intracellular metabolites and control bacterial gene expression. Here, by using X-ray crystallography, molecular dynamics simulations, and single-molecule fluorescence resonance energy transfer, the authors show how a local Mn2+ ion-binding signal is transduced across the *yybP-ykoY* riboswitch from *Xanthomonas oryzae*.

## Introduction

Riboswitches are structured RNA domains commonly found in the 5'-untranslated regions of bacterial mRNAs, where they regulate many essential and virulence genes in response to binding of a specific ligand^1–3^. Currently, there are over 40 different riboswitch classes known to respond to ligands ranging from metabolites^4^, enzyme cofactors^5^, signaling molecules^6–8^, tRNAs^9^, and to metal ions^10–12^. Ligand binding generally stabilizes a conformation of the riboswitch that modulates either Rho-independent transcriptional termination or translation initiation through accessibility of the Shine-Dalgarno (SD) sequence. The static ligand-bound structures, and the ligand-recognition modes, of a number of riboswitch aptamers have been determined at atomic resolution^2,12–14^. Often, the ligand occupies a linchpin position in the global fold where distal residues of the RNA are brought together; however, the dynamic paths by which the local binding of a ligand as small as a metal ion are transduced into the large-scale molecular rearrangements necessary for a regulatory decision by the gene expression machinery largely remain enigmatic^14^.

**Fig. 1:**
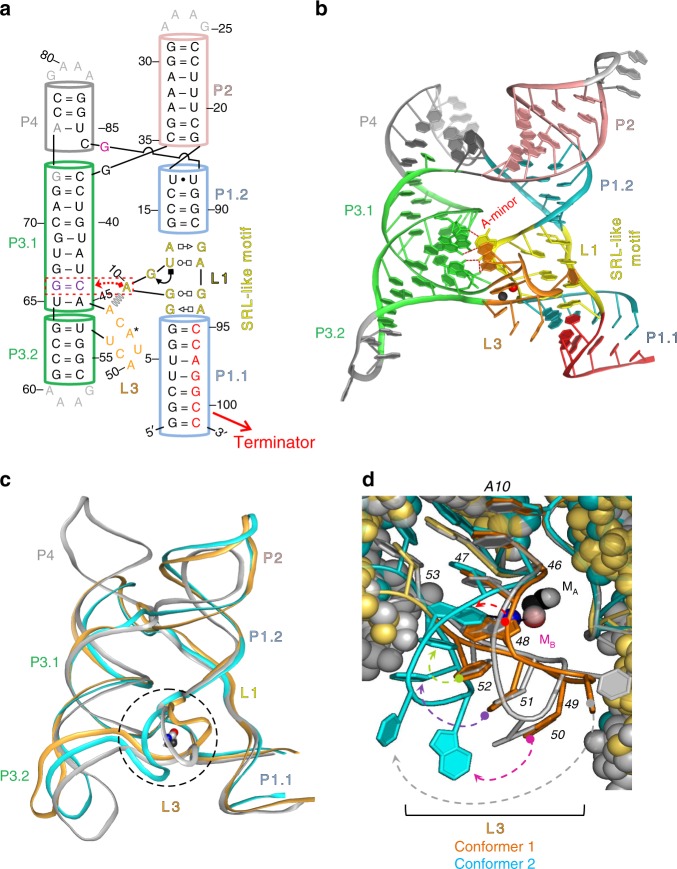
Sequence and structure of the *X. oryzae* (*Xory*) crystal structure. **a** Secondary structure of the *X. oryzae* crystal construct. A native CA dinucleotide was omitted between G73–A74 for crystallization purposes only. The A-minor interaction between L1 and L3 and the SRL-like conformation of L1 is shown. **b** Crystal structure of Conformer 1 (PDB ID: 6N2V) with different secondary structures labeled. **c** Comparison of overall structures of Conformers 1 (orange) and 2 (cyan) and the previous Mn^2+^-bound *L. lactis* structure (gray). The two molecules in the asymmetric unit are overall fairly similar. However, they differ dramatically at the metal ion binding sites (dotted circle). **d** Conformer 1 (orange) is relatively similar to the *L. lactis* structure at the M_B,Mn_ binding site. All the same metal ion contacts are made, although U49, away from the Mn^2+^ site, is shifted (gray dotted arrow). Conformer 2 (cyan) differs in that a metal ion is still bound at the M_B,Mn_ site, but only half of the metal ion contacts are made, and the binding site A48 is flipped (red dotted arrow) to expose N1 rather than N7. U49 (gray arrow), A50 (magenta arrow), C51 (purple arrow), and U52 (green arrow) are all significantly shifted from the previously reported Mn-bound conformation

The *yybP–ykoY* RNA motif is one of the most widespread riboswitches across bacteria, including many human and plant pathogens^15–17^. It has evolved to sensitively detect Mn^2+^ metal ions and broadly regulate a variety of genes, particularly those involved in Mn^2+^ homeostasis, at the levels of either transcription or translation^12,15,18^. We and others have previously solved crystal structures of the aptamer domain of this riboswitch^12,19^, henceforth simply referred to as “riboswitch”, revealing that it senses the charge, geometry, and Lewis-acid hardness of Mn^2+^ by forming direct inner-sphere contacts from five phosphoryl oxygens and the N7 of an invariable adenosine. The global structure showed that formation of the Mn^2+^ binding site requires “docking” of two distal helical legs of a four-way junction (4WJ) to form a paperclip-shaped global architecture, facilitated by an A-minor interaction and a second, nonspecific divalent metal ion binding site (Fig. 1a)^12^. However, the transduction of ligand binding in this metal-sensing core into global structural changes that affect the distal helix P1.1 involved in riboswitching is not understood, rendering it an archetypical representative of our level of understanding of many crystallized riboswitches^14^.

Here, we first solve the structures of two ligand-binding states of the *yybP–ykoY* transcriptional riboswitch from the rice pathogen *X. oryzae*^20,21^, captured in distinct conformations that offer snapshots of structural changes *en route* to full ligand binding. We then use these conformers for atomistic molecular dynamics (MD) simulations that reveal how ligand-dependent local structural perturbations in the metal-sensing core are linked to the stability of distal P1.1 helix to affect riboswitching. Finally, using single-molecule FRET (smFRET), we investigate the global structural dynamics of the riboswitch in the presence of varying concentrations of Mg^2+^ and Mn^2+^, as well as other transition metals, revealing a previously unobserved extended conformation of the riboswitch. We show that addition of Mg^2+^ induces two kinetically distinct docked and undocked conformations that are in dynamic equilibrium with one another. In contrast, upon addition of submillimolar Mn^2+^, the riboswitch adopts a stably docked (SD) conformation that becomes abolished upon mutation of the conserved core adenosine. Taken together, our work reveals the ligand-dependent (un)folding pathway of the Mn^2+^ sensing riboswitch as a guide for how subtle binding preferences distinguishing two similar metal ion ligands cascade through the coupling of local with global RNA conformational dynamics into powerful effects on gene regulation.

## Results

### RNA-based Mn^2+^ sensing by stepwise removal of metal ion hydration

Previously, we determined the structures of the Mn^2+^-bound and Mn^2+^-free states from the *L. lactis* (Llac-Mn PDB: 4Y1I) and *E. coli* (PDB: 4Y1M) *yybP–ykoY* riboswitches, respectively^12^. The Mn^2+^-sensing core was shown to be first bridged via the binding of a Mg^2+^ ion to a nearby site, then undergoes further conformational changes into the final Mn^2+^-bound state. Importantly, Mn^2+^ sensing involves the complete stripping of the Mn^2+^ ion’s hydration shell, substituted with a set of selective metal–RNA contacts. Since completely dehydrating a metal ion is energetically costly, this process was speculated to take place in stepwise fashion, possibly achieved by a stepwise set of local conformational changes in the metal ion sensing loop^12^. However, no structural intermediates have been captured so far; hence our understanding of the selective metal ion sensing process has remained vague.

**Fig. 2:**
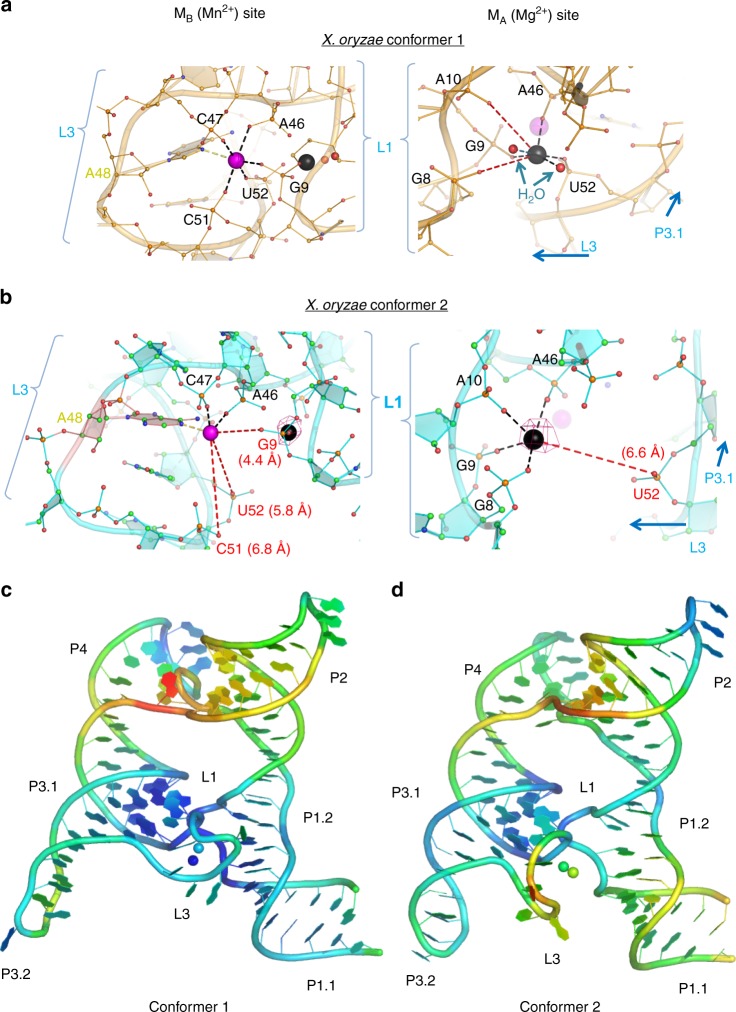
Conformational differences in the metal ion binding sites and B-factors in the structures. Close-up view of the M_A,Mg_ (black) and M_B,Mn_ (purple) metal ion binding sites of **a** Conformer 1 and **b** Conformer 2 showing different contacts with surrounding L1 and L3 residues. Waters are shown as red spheres. The lost metal ion contacts are shown as red dashed lines with their distances. The anomalous difference map at 4*σ* (magenta mesh) shows that Sr^2+^ is not observed in Conformer 1 and weakly occupies the M_A,Mg_ site but not the M_B,Mn_ site in Conformer 2. The Llac-Mn structure is shown in Supplemental Fig. 5 in the same orientation for comparison. Overall *X. oryzae* Conformer 1 (**c**) and Conformer 2 (**d**) structures, colored by individual atomic temperature B-factors (range: 38–135 Å^2^ for Conformer 1, 44–170 Å^2^ for Conformer 2). Notice both are well-structured in L1 and in P3 around the binding site. However, Conformer 1 is much more tightly structured in L3 than is Conformer 2. Also, the metal ions in Conformer 1 are somewhat less variable. In both molecules, there is flexibility around the 4WJ

In this study, we report the partial and complete Mn^2+^ dehydration states, captured in the same crystal lattice of the *X. oryzae yybP–ykoY* riboswitch and resolved to 2.96 Å (Xory-Mn, Fig. 1a, b and Table 1). This riboswitch controls the *yebN* Mn^2+^ efflux pump gene^21^ in *X. oryzae, a* phytopathogenic bacterium that causes rice blight. To facilitate crystallization, the wild-type (WT) *Xory* sequence was modified distally from the metal ion sensing core not to affect metal ion binding, tightening the 4WJ and stabilizing P1.1 to aid crystallization (see Methods, Fig. 1a). The crystal lattice contains two Xory-Mn riboswitch molecules (Supplementary Figs. 1–4 and 6). While their overall architecture is similar to that of the previously determined *L. lactis* 4Y1I (Llac-Mn) structure, with an r.m.s.d of ~2.5 Å, they represent two distinct functional states (Fig. 1c). Conformer 1 is clearly in the canonical Mn^2+^-sensing state despite minor sequence differences in the metal ion binding core, hence providing a comparable reference point for mechanistic interpretation. Its structure superimposes well with the previously determined, canonical, Mn^2+^-bound *L. lactis* structure (Fig. 1c). Both feature two coaxial stacked “legs” connected at the top by the 4WJ, a superimposable metal ion sensing core, with bound Mn^2+^ and Mg^2+^ ions in superimposable positions^12^ (Fig. 1c). As in Llac-Mn, the L1 loop in Xory-Mn adopts a sarcin-ricin-like motif (SRL-like)^19^ and extrudes a conserved A10 to form a cross-helix A-minor interaction with helix P3.1 above L3 (Figs. 1c and 2a, b)^12^. The L3 loop is extruded from the opposite side of the helical stacks, adopting a highly compressed conformation and stacks below A10 in L1. The close juxtaposition of phosphates in the L1–L3 interface creates a metal ion binding hotspot. In Llac-Mn, a largely inner-sphere coordinated (five phosphoryl oxygens and one water molecule) metal ion is located above the A10 residue (Supplementary Fig. 5). Both Mg^2+^ and Mn^2+^ can access this site^12^; we will henceforth refer to it as M_A,Mg_ because the physiological concentration of Mg^2+^ is usually 100-fold higher than Mn^2+^. This metal ion binding site is preserved in Xory-conformer 1, although the inner-sphere contacts to A10 and G8 are either lost or replaced by outer-sphere contacts (Fig. 2a). Of note, although 80 mM SrCl_2_ were present in the crystallization buffer, discernible strontium anomalous signals were found in many sites of Xory-conformer 1 but almost none in the M_A,Mg_ site (Supplementary Fig. 3), suggesting that, like Llac-Mn, Xory-conformer 1 prefers smaller divalent ions such as Mg^2+^ or Mn^2+^ in M_A,Mg_. Mn^2+^ is coordinated specifically in a nearby site (M_B,Mn_), through complete dehydration and a Mn^2+^-specific “soft” contact from the N7 of an invariable adenosine residue (A41 in *Llac* and A48 in *Xory*)^12^ (Fig. 1d). In Llac-Mn, the four residues following the Mn^2+^-sensing adenosine in the L3 loop form a three-residue stack with U43 mediating a U-turn motif^12^. Due to sequence differences (ACA_48_U_49_ACU_52_ in *Xory vs* UCA_41_AU_43_UC in *Llac*), while the three-residue-stacking theme is preserved, the U-turn is found to be mediated by the first residue (U_49_) in Xory-conformer 1, rather than the second residue (U_43_) in Llac-Mn (Fig. 1d and Supplementary Fig. 5). Despite the idiosyncratic folding in the L3 loop, the inner-sphere contacts to Mn^2+^ are mediated by the same set of phosphates and the invariable adenosine, suggesting that they utilize the same mechanism to sense Mn^2+^ (Fig. 2a).

**Table 1:** X-ray crystallographic data collection and refinement statistics for the *X. oryzae* yybP–ykoY Mn^2+^ riboswitch

*Xory yybP–ykoY* riboswitch	Crystal 1
*Data collection*	
Space group	P 21 21 21
Cell dimensions	
*a*, *b*, *c* (Å)	81.32, 85.19, 92.49
*α*, *β*, *γ* (°)	90, 90, 90
Resolution (Å)	62.66–2.96 (3.07–2.96)*
*R*_sym_ or *R*_merge_	0.110 (1.51)
*I* /σ*I*	14.2 (1.26)
Completeness (%)	99.83 (99.63)
Redundancy	81.90
CC1/2	1 (0.599)
CC*	1 (0.866)
*Refinement*	
Resolution (Å)	62.66–2.96 (3.07–2.96)
No. of reflections	13901 (1361)
*R*_work_/*R*_free_	0.205 (0.220)
No. of atoms	
RNA	4214
Ligand/ion	120
Water	7
*B*-factors	
RNA	69.17
Ligand/ion	93.43
Water	66.69
R.M.S. deviations	
Bond lengths (Å)	0.005
Bond angles (°)	0.59
PDB accession code	6N2V

Note that the data were mildly anisotropic, with reflections along the*l*reciprocal axis stronger. Thus, I/sigma for these reflections was well over 1.2 (indeed, over 1.5 up to 2.85 Å resolution)

^*^One crystal was used. Values in parentheses are for highest-resolution shell

Whereas Xory-conformer 1 serves as a reference point to reveal common themes in Mn^2+^ sensing among *yybP–ykoY* riboswitches, Xory-conformer 2 provides a rare, intermediate snapshot of the riboswitch sampling for Mn^2+^ through a partial dehydration process. The Mg^2+^ ion is more loosely coordinated in the M_A,Mg_ site; an inner-sphere coordination from U52 of the L3 loop is lost (Fig. 2b). This is because the L3 loop has yet to adopt its final Mn^2+^-sensing conformation, as the first four residues in L3 have not formed the ordered stacks atop the invariable A48. Importantly, a partially dehydrated metal ion is already present at the M_B,Mn_ site despite lacking half of the contacts seen in Conformer 1, where only the inner-sphere contacts by the phosphates of A45 and A46 are preserved (Fig. 2b). Interestingly, the electron density is sufficiently clear to suggest that the invariable adenosine residue (A48) rotates slightly and orients its N1 group closer than N7 towards M_B,Mn_ (a 2.6 Å outer-sphere contact). Also of note, the temperature B-factors for L3 are higher in Conformer 2 than Conformer 1 (Fig. 2c, d), indicating elevated conformational flexibility. Importantly, this includes larger flexibility around P1.1, suggesting a possible link between the flexibilities of these two key regions. We interpret this conformation as representing the functional state of the *yybP–ykoY* riboswitch sampling metal ions in solution. The partial dehydration process selects a metal ion with an ionic radius similar to Mn^2+^. Mg^2+^ could in theory access the site, however, the N1 and N7 functional groups in the invariable adenosine residue would favor a softer metal ion such as Mn^2+^ (Fig. 2b). We envision that a further conformational transition to that in Conformer 1 would complete the metal ion selection process, as this process would exert additional stringency by not only selecting ionic radius, but also specifying an octahedral coordination and a clear preference for a “soft” transition metal ion (Fig. 2a, b). Taken together, our crystal structures provide important insights into the stepwise metal-sensing process and form the basis for the following MD simulations.

### Effect of M_A,Mg_ and M_B,Mn_ inner-sphere contacts on L3 stacking

To provide deeper insights into the RNA dynamics associated with metal ion identity, we performed 43 atomistic MD simulations, equivalent to a total of 113 µs of real-time (see Supplementary Table 1, Methods), with different metal ions in the M_A,Mg_ and M_B,Mn_ sites and starting either from Xory-Mn Conformers 1 or 2 (as reported here) or from *L. lactis* structures (PDB IDs 4Y1I and 6CB3)^12,19^. We found that the arrangement of the ion binding sites in chain A of the 6CB3 *L. lactis* structure involved three Cd^2+^ ions and required the deprotonated form of uracil U44^−^ to prevent loss of its first-shell coordination in the simulations. In addition, the replacement of the Cd^2+^ ions by one Mn^2+^ and two Mg^2+^ ions in the M_B,Mn_, M_A,Mg_, and M_C_ ion binding sites resulted in modest reorganization of their first-shell coordination spheres even in the presence of a deprotonated U44^−^. Therefore, this particular arrangement of ion binding sites appears to be specific for large Cd^2+^ ions, likely due to their enhanced affinity to nitrogen ligands and preference for hepta-coordination over the native Mn^2+^ ion (Supplementary Discussion, Supplementary Note 3 and Supplementary Figs. 11–14). Therefore, we view this specific arrangement around the toxic and xenobiotic Cd^2+^ ions as not fully relevant for physiological Mn^2+^/Mg^2+^ ionic conditions.

**Fig. 3:**
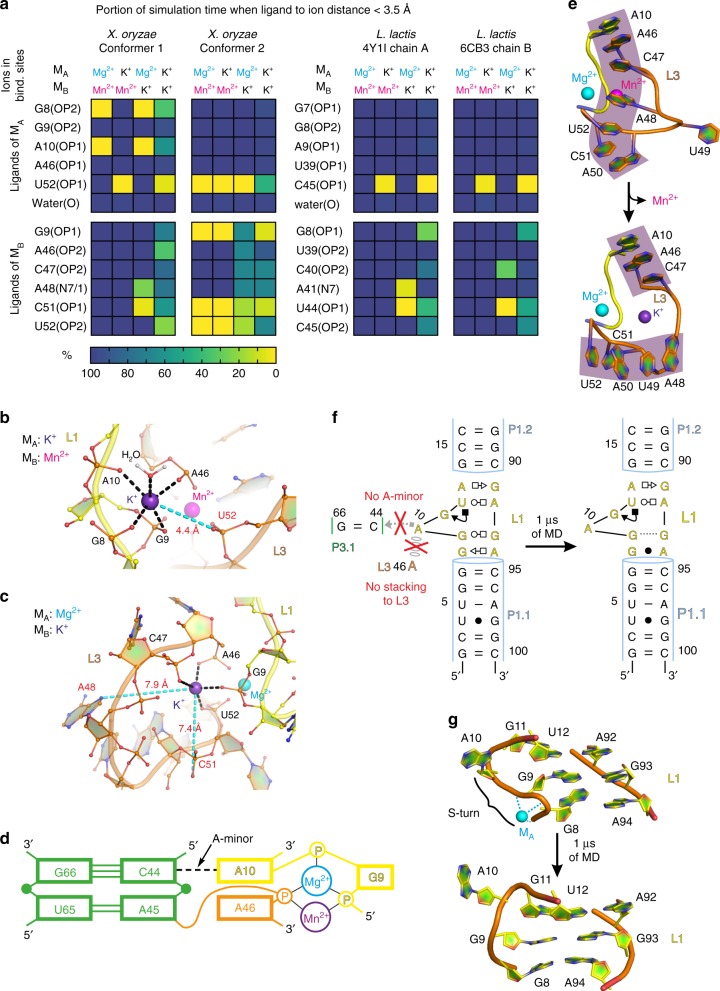
Structural dynamics of inner-shell ligands of the M_A,Mg_ and M_B,Mn_ sites, L3 and L1 loops as revealed by MD simulations. **a** The population of native inner-shell contacts in the M_A,Mg_ and M_B,Mn_ sites in percent as a function of the type of ion in these sites. A representative structure of the **b** M_A,Mg_ and **c** M_B,Mn_ site as typically observed in MD simulations with a K^+^ ion occupying the site. The most labile inner-shell contacts are depicted in cyan. **d** 2D representation of the most stable part of the L1–L3 tertiary interaction that is likely anchored by the A-minor interaction and related A10|A46 stacking. **e** Partial unfolding of the stacking pattern in the L3 loop observed upon replacement of Mn^2+^ with K^+^ in the M_B,Mn_ site. **f** The loss of native G9–G93 *trans* Watson-Crick/Hoogsteen and G8–A94 *trans* Sugar-Edge/Hoogsteen base pairing, and **g** loss of the S-turn backbone conformation involving a phosphate notch forming part of the M_A,Mg_ ion binding site in response to loss of the L1–L3 tertiary interaction with its A10…G66 = C44 A-minor interaction

As expected, given the limited timescales compared to experiments^22,23^, we found that our simulations generally maintained quite stable inner-sphere contacts for divalent metal ions, including their coordination with water molecules (Fig. 3a). To accelerate these dynamics, we performed additional MD simulations that replaced one or both divalent ions at the M_A,Mg_ and M_B,Mn_ ion binding sites with monovalent K^+^ ions (Supplementary Table 1). Replacement of divalent ions by monovalents increases flexibility of the starting ion-binding arrangement, which allows us to observe more local dynamics on the simulation timescale while retaining most relevant aspects of ion binding, since monovalent and divalent ions do compete for the same binding sites.^22,23^ Indeed, the global architecture of the riboswitch was maintained except for local perturbations of L3 and a variable P2–P4 interhelical angle (Supplementary Figs. 7, 8, and Supplementary Note 1).

The simulations revealed that the inner coordination spheres of monovalent ions in both the M_A,Mg_ and M_B,Mn_ sites were significantly more stable when the second binding site was occupied by a divalent rather than monovalent ion (Fig. 3a). This observation suggests that binding of the divalent ion into one binding site helps pre-organize and stabilize the second binding site, consistent with the notion of cooperativity between M_A,Mg_ and M_B,Mn_. Furthermore, while some experimentally observed inner-sphere contacts remained very stable upon replacement with a monovalent, other contacts were lost (Fig. 3a–c). Notably, the bridging inner-sphere contacts of the U52 phosphate with the M_A,Mg_ ion and those of the C51 and G9 phosphates with the M_B,Mn_ metal ion were found to be most labile across all simulations (Fig. 3a, Supplementary Table 2, and Supplementary Figs. 15–19). These are the same inner-sphere contacts found to be lost in the crystallized Conformers 1 and 2 compared to the 4Y1I and 6CB3 (chain B) *L. lactis* structures, supporting the notion that the crystallized *X. oryzae* Conformers are relevant as *en route* to (un)folding of the metal ion binding sites. In contrast, the inner-shell contacts of the A46 phosphate with the ions in both M_A,Mg_ and M_B,Mn_, as well as that of the G9 phosphate with the ion in M_A,Mg_, were stable across all simulations (Fig. 3a, Supplementary Table 2, and Supplementary Figs. 15–19). These interactions are likely stabilized by coupling of the A-minor interaction of A10 to the G66–C44 base pair and associated stacking of A10 on the neighboring A46. All these interactions are parts of the L1–L3 tertiary docking contact and remained stable across all simulations (Fig. 3d).

We also found the dynamics of the entire L3 loop to be sensitive to the type of ion in the M_B,Mn_ site. When Mn^2+^ in the M_B,Mn_ site was replaced with K^+^, the flexibility of the L3 loop was increased so that the loop populated various conformations with broken stacking patterns (Fig. 3e and Supplementary Note 4). These perturbations originated from the weakened inner-sphere contact of the A48(N7) nitrogen to the M_B,Mn_ metal, suggesting that the L3 loop is involved in direct sensing of the Mn^2+^ ion by forming a linchpin to support a tight stacking patterns only in the presence of the native Mn^2+^ (Fig. 3e).

### Stability of the L1 loop requires an A-minor interaction

To complement our probing of structural dynamics in the docked state with those in the undocked state, we performed additional MD simulations of the segment consisting only of P1.1, P1.2, and L1. We started these undocked simulations from either of the two Xory-Mn Conformers and Llac-Mn structure (Supplementary Table 1), with the aim to reveal their structural dynamics in the absence of the L1–L3 docking contact. While the L1 loop in the context of the entire riboswitch with its docked L1–L3 interaction always populated the SRL-like conformation, two of our three undocked simulations lost this motif (Fig. 3f, g, Supplementary Fig. 22, and Supplementary Note 5). In particular, in one of the simulations we observed loss of the G9–G93 *trans* Watson–Crick/Hoogsteen and G8–A94 *trans* Sugar-Edge/Hoogsteen base pairing^24^ as well as the S-turn^25^ that forms part of the M_A,Mg_ site (Fig. 3f, g). The former two base pairs are coaxially stacked on the P1.1 stem, suggesting that their loss may highlight the beginning of a transduction path by which P1.1 could become destabilized.

### smFRET reveals an undocked state that docks upon Mg^2+^ addition

**Fig. 4:**
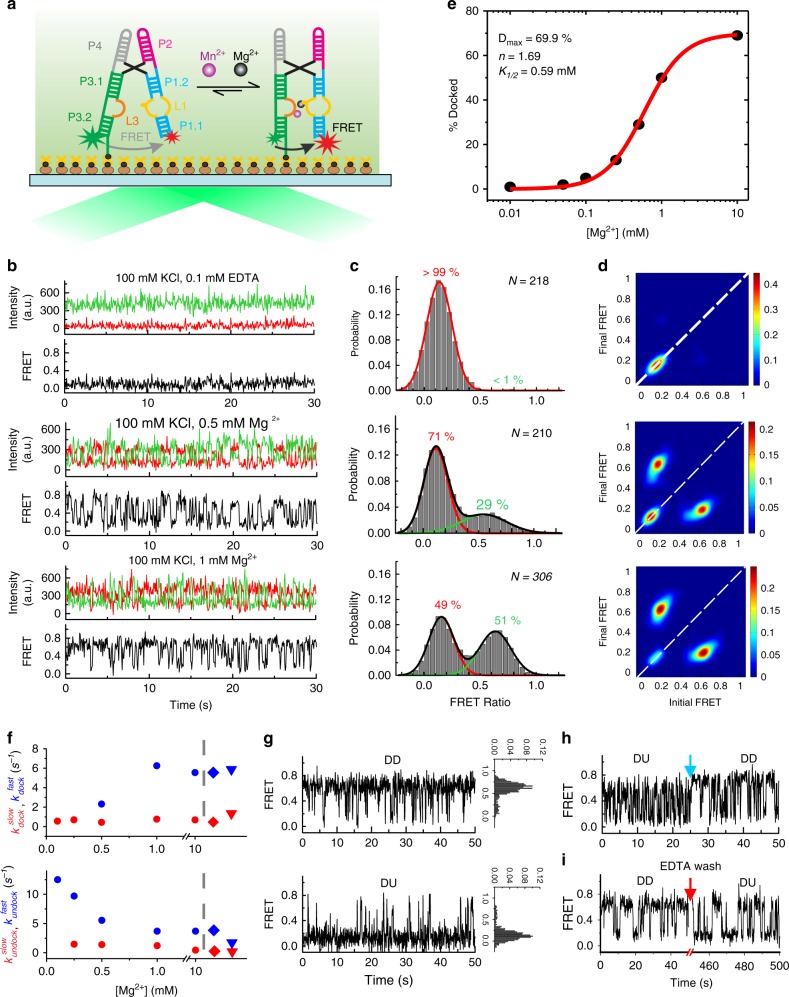
smFRET analysis of the WT *Xory* Mn^2+^ riboswitch. **a** Schematic of the smFRET experiment using TIRFM indicating the fluorophore labeling positions on the riboswitch. **b** Representative smFRET traces under different buffer conditions (top–bottom): no divalents (+0.1 mM EDTA), 0.5 mM Mg^2+^ and 1 mM Mg^2+^, respectively. Green, Cy3; Red, Cy5; Black, FRET. **c** Population FRET histograms showing the equilibrium distribution of two FRET states under the conditions in panel (**b**). Gaussian peaks for the low- and high-FRET states are shown in red and green, respectively with the cumulative fit shown in black. Reported are the percentages of FRET states at equilibrium, as well as the number of molecules N analyzed. **d** TODPs showing the static and dynamic trances as “on-diagonal” and “off-diagonal” heat map contours, respectively. The color code indicates the fraction of each population. **e** Fraction of the high-FRET state as a function of Mg^2+^ concentration, fit with a standard Hill equation (red). **f** Kinetics of structural dynamics as a function of Mg^2+^ and Mn^2+^ concentration. The diamond symbols represent rates in 1 mM Mg^2+^ and 0.1 mM Mn^2+^ while the triangle symbols represent rates in 0.1 mM Mn^2+^ alone. **g** Exemplary dynamic docked (DD, top) and dynamic undocked (DU, bottom) traces in the presence of 1 mM Mg^2+^. FRET histograms for the individual traces are shown on the right. **h** Rare examples of interconversion between different kinetic regimes showing dynamic heterogeneity. The blue arrow indicates the time of switching between the two kinetic regimes. **i** Exemplary trace showing conversion of a DU trace to DD trace upon chelation of Mg^2+^ with EDTA (indicated by the red arrow) and reintroducing 1 mM Mg^2+^. Source data for panels (**e**) and (**f**) are provided as a Source Data file

To probe the global structural dynamics in the presence of Mg^2+^ and Mn^2+^, we used smFRET to monitor fluorophores positioned on the distal legs of the *Xory* riboswitch (Fig. 4a, Supplementary Fig. 23, and Methods). smFRET traces at 100 mM KCl in the absence of any divalent metal ions showed a stable low-FRET value of ~0.1 without global dynamics (Fig. 4b), with a population FRET histogram displaying a single peak centered on ~0.13 ± 0.10 (mean ± standard deviation) (Fig. 4c). The nondynamic nature of the traces is also evident as an on-diagonal contour centered at ~0.13 in the transition occupancy density plot (TODP), which represents as a heat map the fraction of single molecule traces that exhibit any given specific initial-to-final FRET transition at least once (Fig. 4d and Methods)^26^. This FRET value corresponds to an estimated distance of ~74 Å between the two fluorophores and suggests an extended, stably undocked (SU) conformation where the two RNA legs are distal and do not interact, unlike the docked crystal structure (Fig. 1b).

Addition of Mg^2+^ up to 0.1 mM does not result in significant changes in the FRET histograms since almost all traces remain in the SU conformation, with ~3% of them showing brief excursions into a higher ~0.6 FRET state (Supplementary Fig. 24). Further raising the Mg^2+^ concentration results in more dynamic traces transiently adopting this high-FRET state, accompanied by a corresponding decrease in the population of SU traces (Fig. 4b–d and Supplementary Fig. 24). At a near-physiological concentration of 1 mM Mg^2+^, the time- and population-averaged distribution between low- and high-FRET, with mean FRET values of ~0.15 ± 0.11 (49%) and 0.63 ± 0.14 (51%), respectively, became almost equal (Fig. 4c). A FRET value of 0.63 corresponds to a distance of ~49 Å between the two labeled RNA arms, similar to the distance observed in the crystal structures, suggesting adoption of the compact “docked” conformation. Reaching 10 mM Mg^2+^, the fraction of docked conformation further increases and saturates at ~69%, with a sigmoidal Mg^2+^ concentration dependence that fit well with a Hill equation to yield a half-saturation point of *K*_1/2_ ~ 0.6 mM and a cooperatively coefficient of *n* = 1.7 (Fig. 4e). These data demonstrate that the Mn^2+^ riboswitch adopts an extended SU conformation in the absence of divalents, which increasingly samples transient docked conformations upon a rise in Mg^2+^ concentration.

At a low-Mg^2+^ concentration of 0.1 mM, single-exponential kinetics are observed with a slow docking rate constant, *k*_dock_ ~ 0.56 s^−^^1^, and a fast undocking rate constant, *k*_undock_ ~ 12.5 s^−1^ (Fig. 4f and Supplementary Fig. 25). Further increasing the Mg^2+^ concentration to 1 mM results in the emergence of double-exponential kinetics in both *k*_dock_ and *k*_undock_. The docking kinetics exhibit $k_{\mathrm{dock}}^{\mathrm{fast}}\,\sim 6.25\,{\mathrm{s}}^{ - 1}$ and $k_{\mathrm{dock}}^{\mathrm{slow}}\,\sim 0.76\,{\mathrm{s}}^{ - 1}$, while the undocking kinetics display $k_{\mathrm{undock}}^{\mathrm{fast}}\,\sim 3.70\,{\mathrm{s}}^{ - 1}$ and $k_{\mathrm{undock}}^{\mathrm{slow}}\,\sim 1.21\,{\mathrm{s}}^{ - 1}$ (Fig. 4f and Supplementary Fig. 25). The TODP further shows that a majority (82%) of traces are dynamically transitioning between the two FRET states, as highlighted by dominant off-diagonal contours, while only a small fraction (~18%) remains in the stable low-FRET state (Fig. 4d). The double-exponential kinetics arises from two distinct populations: dynamic docked (DD) and dynamic undocked (DU) traces corresponding to molecules residing largely in the docked and undocked states, respectively (Fig. 4g). Among the dynamic traces, ~65% were DD, while ~35% were DU traces. As observed for other RNAs that undergo docking of two adjacent helical arms^27–33^, the heterogeneity observed in the population is largely static and molecular behaviors interconvert only rarely (<2% of traces) over the experimental timescale (5–10 min) (Fig. 4h). Interconversion between the DU and DD behaviors is observed more readily, however, when first chelating, then reintroducing Mg^2+^ (Fig. 4i), suggesting that they represent kinetically trapped conformations on a deeply rugged folding free energy landscape^34,35^.

### Submillimolar Mn^2+^ uniquely yields a SD riboswitch

**Fig. 5:**
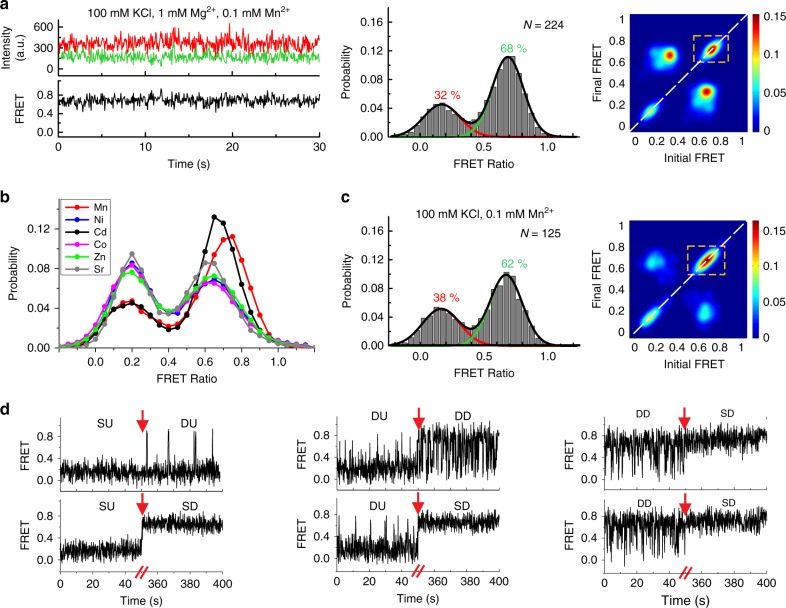
Effect of Mn^2+^ on the folding of the riboswitch. **a** Representative smFRET trace, FRET histogram and TODP for the WT riboswitch in the presence of 0.1 mM Mn^2+^ and 1 mM Mg^2+^. The stable docked (SD) conformation appears only in the presence of Mn^2+^, as highlighted by the dashed yellow box in the TODP. **b** FRET histograms for the WT riboswitch at 0.1 mM concentration of different transition metals in the presence of 1 mM Mg^2+^. Over 150 molecules were analyzed for each histogram. The corresponding TODPs are shown in Supplementary Fig. 26. **c** FRET histogram and TODP with SD population highlighted by dashed yellow box for the WT riboswitch in the presence of 0.1 mM Mn^2+^ alone. **d** Example smFRET traces for the WT riboswitch showing transitions between SU, DU, DD, and SD traces upon addition of 0.1 mM Mn^2+^. The time of Mn^2+^ addition is indicated by the red arrow

We next asked what specific effect Mn^2+^ has on the folding of the riboswitch. In the presence of 1 mM Mg^2+^, addition of 0.1 mM Mn^2+^ results in the appearance of a unique population of SD (~43%) traces residing in the high-FRET state for >30 s (*k*_undock_ < 0.03 s^−1^) before photobleaching (Fig. 5a). Accordingly, the FRET histogram shows two peaks with mean FRET values of 0.17 ± 0.14 and 0.69 ± 0.12 and an increased ~68% population of the docked conformation (Fig. 5a). The SD population is evident in the TODP as a new on-diagonal contour centered on the ~0.7-FRET value (Fig. 5a). Double-exponential kinetics similar to the 1 mM Mg^2+^ alone condition are observed, with $k_{\mathrm{dock}}^{\mathrm{fast}} = 5.55\,{\mathrm{s}}^{ - 1}$, $k_{\mathrm{dock}}^{\mathrm{slow}} = 0.46\,{\mathrm{s}}^{ - 1}$, $k_{\mathrm{undock}}^{\mathrm{fast}} = 3.84\,{\mathrm{s}}^{ - 1}$ and a ~5-fold slower $k_{\mathrm{undock}}^{\mathrm{slow}}$ of 0.23 s^−1^. Notably, in the presence of Mn^2+^, most of the dynamic traces show DD character. These data demonstrate that Mn^2+^ binding stabilizes the docked conformation while uniquely enabling an SD state. FRET histograms further show that out of a variety of divalent metal ions tested, only Cd^2+^ is effective in promoting docked conformations over 1 mM Mg^2+^ alone; Ni^2+^, Co^2+^, Sr^2+^, or Zn^2+^ had little effect (Fig. 5b and Supplementary Fig. 26). Interestingly, in the absence of Mg^2+^, while 0.1 mM Mn^2+^ alone leads to the appearance of DD and SD traces with ~62% docked population (mean FRET 0.67 ± 0.12) (Fig. 5c), 0.1 mM of Ni^2+^, Co^2+^, Sr^2+^, or Zn^2+^ does not affect SU traces and Cd^2+^ has only a small effect in promoting DD traces (Supplementary Note 6 and Supplementary Fig. 26). These results suggest that while the *Xory* riboswitch has some degree of plasticity in recognizing ligands in a background of Mg^2+^, it preferentially recognizes Mn^2+^ and—to a lesser extent—Cd^2+^.

To probe how the kinetically distinct SU, DU, and DD traces respond to Mn^2+^, we observed the same set of molecules at 1 mM Mg^2+^, before and after the addition of Mn^2+^. We find that all three populations respond to Mn^2+^ and are capable of forming the stably docked (SD) population (Fig. 5d). In particular, the SU traces convert into DU, DD, and SD conformations with similar probabilities, suggesting that they are correctly folded with metal-sensing sites poised to bind ligand. By comparison, a majority of DD traces convert into SD traces, whereas DU traces adopt DD and SD behavior upon Mn^2+^ addition. Only a small fraction of traces show no response at low Mn^2+^ suggesting that they may be misfolded.

### Mutation of A48 results in complete loss of SD conformation

**Fig. 6:**
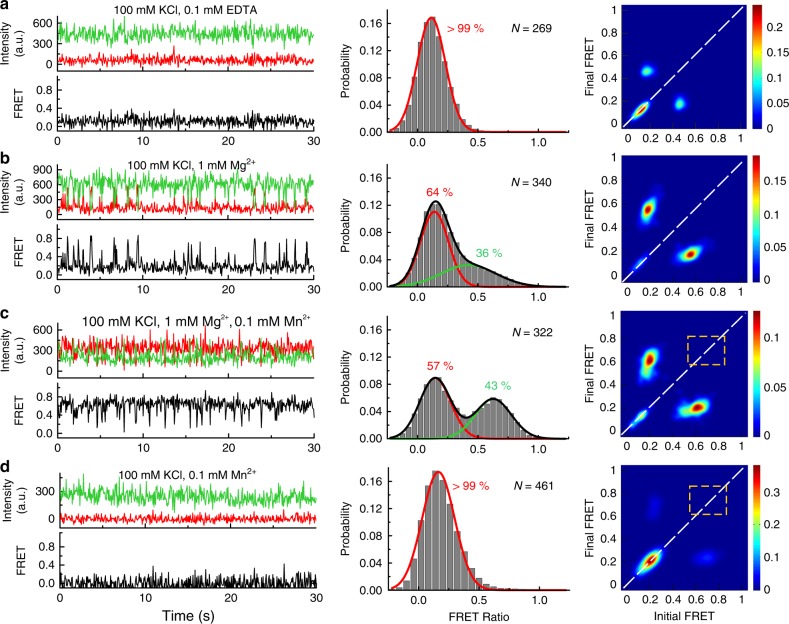
Mutation of the Mn^2+^ sensing A48 leads to loss of the SD conformation. Representative smFRET traces, FRET histograms and TODPs for the A48U mutant riboswitch under: **a** no divalents (+0.1 mM EDTA), **b** 1 mM Mg^2+^ alone, **c** 1 mM Mg^2+^ and 0.1 mM Mn^2+^, and **d** 0.1 mM Mn^2+^ alone. The dashed yellow box highlights the absence of SD conformations for the A48U riboswitch in the presence of Mn^2+^, in contrast to the WT riboswitch

The highly conserved discriminator base A48 in L3 is positioned to confer Mn^2+^ specificity via its N7 and also helps maintain L3 in a stacked conformation. We, therefore, tested the effect of a single A48U mutation on folding and Mn^2+^ sensing of the riboswitch. Similar to the WT, at 100 mM KCl without divalents, the mutant riboswitch shows SU population with a mean FRET value of 0.11 ± 0.12 (Fig. 6a). At 1 mM Mg^2+^, we observe dynamic traces with excursions into the docked higher FRET states, and the FRET histogram shows a major (64%) 0.14 ± 0.11 low-FRET peak and a minor (36 %) broad 0.52 ± 0.21 mid-FRET peak that corresponds to more extended docked conformations (Fig. 6b). Of note, 100% of the dynamic mutant traces show DU character under this condition (Fig. 6b). As a result, we observe fast single-exponential kinetics with *k*_undock_ ~ 6.67 s^−1^, while *k*_dock_ was double-exponential with a major $k_{\mathrm{dock}}^{\mathrm{fast}} = 3.70\,{\mathrm{s}}^{ - 1}$ (95%) and a minor $k_{\mathrm{dock}}^{\mathrm{slow}} = 0.46\,{\mathrm{s}}^{ - 1}$ (5%) (Supplementary Fig. 27). The TODPs show SU behavior in the absence of divalents, whereas both SU and DU behaviors are observed at 1 mM Mg^2+^ (Fig. 6a, b).

Next, we asked whether the mutant riboswitch can still respond to Mn^2+^. In the presence of 1 mM Mg^2+^ and 0.1 mM Mn^2+^, most smFRET traces show DD behavior, while only a small fraction remain in the SU state, similar to WT (Fig. 6c). FRET histograms show a similar ~0.14 ± 0.13 (57%) low-FRET undocked state but display a docked state now with a higher mean-FRET value of 0.63 ± 0.15 and a larger ~43% population, compared to the 1 mM Mg^2+^ only condition. The kinetics under these conditions are double-exponential with a $k_{\mathrm{dock}}^{\mathrm{fast}} = 6.67\,{\mathrm{s}}^{ - 1}$ (89%), $k_{\mathrm{dock}}^{\mathrm{slow}} = 0.64\,{\mathrm{s}}^{ - 1}$ (11%), $k_{\mathrm{undock}}^{\mathrm{fast}} = 3.10\,{\mathrm{s}}^{ - 1}$ (94%), $k_{\mathrm{undock}}^{\mathrm{slow}} = 0.88\,{\mathrm{s}}^{ - 1}$ (6%) (Supplementary Fig. 27). Importantly, the mutation causes a striking loss of SD traces, as evident from the complete absence of the on-diagonal ~0.7 FRET contour in the TODP (Fig. 6c), in stark contrast to the WT. In addition, the mutant interestingly loses the WT’s ability to sample the docked conformations in the presence of 0.1 mM Mn^2+^ alone (Fig. 6d); almost all smFRET traces at 0.1 mM Mn^2+^ are in the SU conformation, with the histogram showing a major single peak around 0.19 ± 0.13 FRET, further corroborated by a single on-diagonal ~0.2 FRET contour in the TODP (Fig. 6d). These data demonstrate that the conserved discriminator A48 is essential for Mn^2+^ inducing a SD riboswitch conformation. The presence of Mg^2+^ partially rescues the loss of Mn^2+^ sensing by the mutant, yet does not restore its ability to form the SD conformation. Possibly this reflects the ability of Mg^2+^ to bind at M_B_ in A48U, though with weaker affinity, via the uridine O4, as suggested in the A41U *L. lactis* structure^12^.

### Mn^2+^-binding enhances the stability of switch helix P1.1

**Fig. 7:**
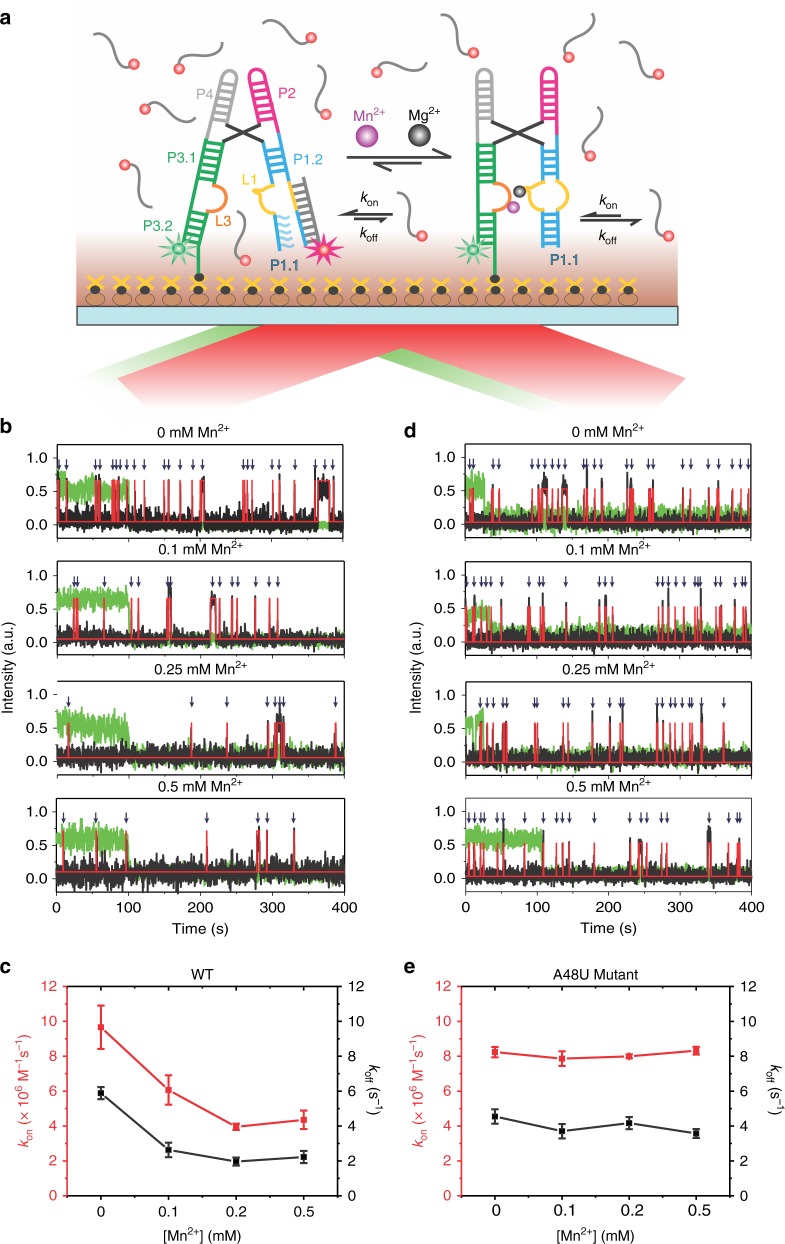
Accessibility of switch helix P1.1 probed using a SiM-KARTS assay. **a** Schematic of our SiM-KARTS assay to probe the accessibility of switch helix P1.1 in the presence and absence of Mn^2+^. **b** Representative SiM-KARTS traces at different Mn^2+^ concentrations show multiple binding events of the Cy5-labeled DNA oligonucleotide probe. Green, Cy3 intensity; black, Cy5 intensity; red, idealized Cy5 intensity fit to a two-state model. **c** The association (*k*_on_, red) and dissociation (*k*_off_, black) rate constants for binding of probe to P1.1 as a function of Mn^2+^ concentration for the WT riboswitch. The errors for the rate-constants are obtained using a boot-strapping program, as described in Methods. **d**, **e** Same as in (**b**) and (**c**), but for the A41U mutant riboswitch. Over 200 traces were analyzed for each condition to obtain reliable kinetic rate constants. The data for kinetic plots in (**c**) and (**e**) are provided in the Source Data file

The proposed mechanism of transcription antitermination of the *yybP–ykoY* riboswitch, supported by our structural and dynamic data, involves Mn^2+^-mediated stabilization of the inherently weak P1.1 switch helix. To more directly test this prevailing hypothesis, we probed the accessibility of the 3'-half of the P1.1 helix (last 12-nt) *in trans* by a complementary Cy5-labeled DNA oligonucleotide probe using our established single molecule kinetic analysis of RNA transient structure (SiM-KARTS) assay (Fig. 7a)^36^. In the current configuration, SiM-KARTS mimics the strand invasion by the downstream terminator sequence and reports on the stability of P1.1 helix at the single-molecule level. In the presence of 10 nM Cy5-labeled oligonucleotide and 1 mM Mg^2+^, repeated short binding events are observed with a probe binding rate constant *k*_on_ of 9.67 ± 1.24 × 10^6^ M^−1^ s^−1^ and a dissociation rate constant *k*_off_ of 5.88 ± 0.35 s^−1^ for the WT riboswitch (Fig. 7b, c). Addition of increasing concentrations of Mn^2+^ results in a decrease in the frequency of binding events, with a *k*_on_ value significantly reduced by ~2.5-fold in the presence of 200 µM Mn^2+^ (Supplementary Figs. 28 and 29). These data report on a decreasing accessibility of the 3'-segment of the P1.1 helix and thus a more stable P1.1 duplex in the presence of Mn^2+^. Interestingly, the *k*_off_ value also decreases with the addition of Mn^2+^, consistent with the notion that the RNA can still sense Mn^2+^ via interaction of the free 5'-half of P1.1—extending into the L1 loop—with loop L3, resulting in a stable L1 and P1.2 that can stack on top of the RNA–DNA hybrid. Remarkably, the effect of Mn^2+^ is completely lost in the A48U mutant, where the *k*_on_ and *k*_off_ values remain essentially unchanged at 8.24 ± 0.30 × 10^6^ M^−1^ s^−1^ and 4.54 ± 0.41 s^−1^, respectively, even at high (500 µM) Mn^2+^ concentration, showing no change in the frequency of individual binding events (Fig. 7d, e and Supplementary Figs. 28 and 30). These data demonstrate that the stability of P1.1 is enhanced once the riboswitch encounters Mn^2+^, an effect lost upon mutation of the Mn^2+^ sensing adenosine. These results thus provide a direct evidence for the prevailing mechanistic hypothesis of riboswitching by the *yybP–ykoY* RNA, wherein Mn^2+^ binding stabilizes the antiterminating P1.1 switch helix through long-range signal transduction from the metal ion sensing core, thereby preventing formation of the competing terminator hairpin.

## Discussion

Using a combination of X-ray crystallography, MD simulations and smFRET, our work here sheds light on how the binding of a single Mn^2+^ ion at a distant site can modulate the stability of the switch helix through a conformational cascade that couples local with global structural dynamics. While previous structures revealed the local Mn^2+^ recognition mechanism of *yybP–ykoY* RNA^12,19^, the structures reported here underscore the conformational plasticity around the metal ion binding core. Importantly, the structural differences observed between the two conformers in the crystal lead us to posit that Conformer 2 with L3 partially unfolded around M_B,Mn_ is likely an intermediate between the fully Mn^2+^-bound state and the empty M_B,Mn_ state observed in the *E. coli* Mn^2+^-free structures^12^. We hypothesize that the loss of phosphate contacts from C51, U52, and G9 to Mn^2+^ in Conformer 2 reflects their relative weakness as supported by MD simulations and likely represents a discrete step in Mn^2+^ binding/unbinding. Conversely, the variation in M_B,Mn_ site coordination we observe could represent inherent flexibility at this site, allowing it to recognize Mn^2+^ even if the metal ion is not fully dehydrated. This flexibility is also seen in the recent Cd^2+^-bound structures^19^ (see Supplementary Discussion).

Our MD simulations show that the SRL-like motif in L1 is inherently unstable in the L1–L3 undocked state, but is stabilized by the tertiary A-minor interaction of A10 with P3.1. Similarly, L1–L3 docking stabilizes L3 in a stacked conformation by direct stacking of A10 on top of it. Thus, a stable conformation of L1 and therefore P1.1 is intimately linked to the stability of L3 and the tertiary A-minor interaction. We suggest that the SRL-like motif acts as a molecular switch that links the Mn^2+^-dependent increase in docking observed by smFRET to stabilization of P1.1.

The observation of pairs of stably and dynamically docked and undocked states with double-exponential kinetics supports the existence of at least two undocked and docked dynamic (in addition to the static SU and SD) conformations, with similar FRET values and thus global folds, but likely different local conformations around the ligand sensing core. These conformations are possibly related to Conformers 1 and 2 in the crystal structure. We further find that SD conformations are formed only in the presence of submillimolar Mn^2+^ or the related soft transition metal ion Cd^2+^, and lost upon a single mutation of the invariant Mn^2+^-sensing adenine A48. While our work was in preparation, another smFRET study on the folding of a different *Llac* Mn^2+^ riboswitch became available. Using a similar labeling strategy, this *Llac* riboswitch showed two FRET states similar to the *Xory* riboswitch^37^. Interestingly, the kinetics of conformational dynamics for the *Llac* riboswitch were largely single-exponential, suggesting a two-state folding behavior, that agrees with a single conformation of the ligand-bound structure. This is in contrast to the *Xory* riboswitch studied here, which shows double-exponential kinetics, consistent with the two distinct conformations of the ligand-bound structure seen in our crystal structures. Such species-specific differences in the Mg^2+^ and Mn^2+^ mediated folding of the *yybP–ykoY* riboswitches likely enable bacteria to elicit a fine-tuned response to changes in their intracellular metal ion concentrations.

**Fig. 8:**
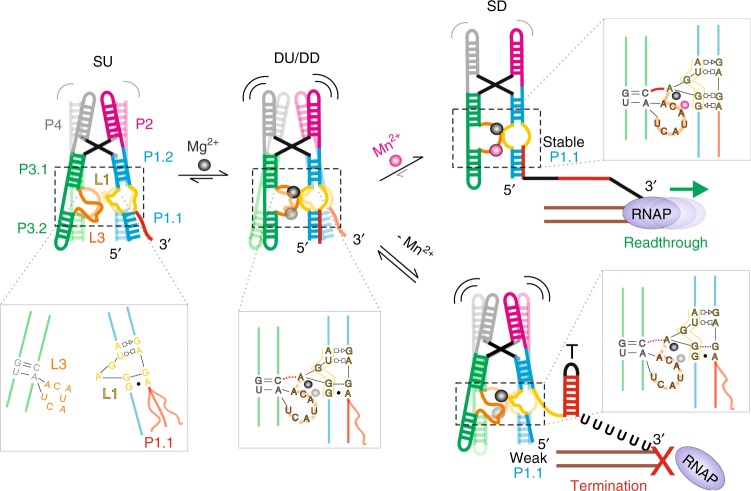
Local-to-global signal transduction pathway in the core of the *Xory* Mn^2+^ sensing riboswitch. The *Xory* riboswitch exists in an “X-shaped” extended SU conformation in the absence of Mg^2+^, with very rare transitions into docked “H-shaped” conformations. In the SU state, the legs are far apart in the absence of a stable A-minor tertiary interaction and loops L3 and L1 are less structured under these conditions. In the presence of physiological Mg^2+^ concentrations (mM), the riboswitch samples docked conformations with distinct kinetics, indicative of differences in L3 stacking as observed in our crystal structures. L1 adopts an S-turn conformation (as indicated by the yellow box in the inset) and L3 is partially stacked under these conditions, aided by the presence of Mg^2+^ ions in M_A,Mg_ and transiently in M_B,Mn_. In the presence of sufficient Mn^2+^, binding of the metal ion by A48 reinforces L3 stacking and stability of the A-minor interaction. This maintains L1 in a rigid noncanonical SRL-like conformation that enables co-axial stacking between P1.2 and the switch helix P1.1. In turn, this cascade of interactions stabilizes P1.1, thereby preventing strand invasion to favor formation of the antiterminator required for transcriptional read-through

Our structural snapshots, MD simulations and smFRET data support the long-range structural signal transduction model shown in Fig. 8. In the absence of any divalent ions, the *Xory* riboswitch adopts an extended conformation in which the two legs are distal and rarely interact, as shown by smFRET. Under these conditions, the metal-ion binding sites are likely unstructured and their interaction only transiently formed by L1–L3 contacts, as suggested by rare smFRET transitions, MD simulations, as well as previous ligand-free structures^12^. MD simulations in particular show that the A-minor interaction formed by A10 of L1 loop with the G–C base pair of stem P3.1 can at least transiently form even in the absence of divalents. The formation of this A-minor interaction helps pre-organize the M_A,Mg_ binding site. Under physiological (1 mM) concentrations of Mg^2+^, this ion binds first at the M_A,Mg_ site, allowing the riboswitch to sample dynamic folded conformations where the two legs are brought together via metal ion-mediated interaction between loops L1 and L3. These dynamic folded states then allow for local interactions mediated by L3, forming a pocket of high negative charge potential poised to sense Mn^2+^. Mg^2+^ binding at M_A,Mg_, therefore facilitates binding of Mn^2+^ at M_B,Mn_ in cooperative fashion, as suggested by smFRET at the global level and further visualized by atomistic MD simulations. Capture of Mn^2+^ at site M_B,Mn_ then acts as the final linchpin to hold the two legs together most stably. This compact docked conformation with a SRL-like fold of L1 and continuous coaxial stacking between P1.2, L1, and P1.1 stabilizes the remote P1.1 switch helix, as shown by our SiM-KARTS assay and MD simulations, that prevents the strand invasion required to form the terminator hairpin, instead promoting transcriptional read-through.

Contrasting with the Mn^2+^ riboswitch, the NiCo riboswitches, which cooperatively sense the transition metals Ni^2+^ or Co^2+^, resemble the overall “H” shaped architecture of the Mn^2+^ riboswitch but with a tight 4WJ that lacks an analogous tertiary docking interface. Accordingly, the NiCo riboswitch appears to act through a distinct mechanism utilizing four bound metal ions to weave together a network of interactions between the interhelical residues that stabilize the 4WJ and so prevent formation of a terminator via strand invasion^38^. Similarly, the Mg^2+^ sensing M-box riboswitch adopts an architecture with three parallel co-axially stacked helices that are brought together by the binding of six Mg^2+^ ions, leading to formation of a terminator^39^. Therefore, both the NiCo and Mg^2+^ sensing riboswitches achieve gene regulation by employing multiple metal ions that directly interact with and stabilize the switch helix. In contrast, the Mn^2+^ riboswitch is unique in requiring only a single-metal ion that does not make any direct contacts with switch helix P1.1. By combining high-resolution structural information with insights into both atomistic and global dynamics, our study outlines the mechanism by which a ligand as small as a single divalent metal ion couples local with global structure to give Mn^2+^ the ability to influence RNA folding and fine-tune gene expression by cooperating with a high background of Mg^2+^. The general lessons revealed here of how a ligand-binding signal can be transduced across an RNA are likely to become a recurring theme among riboswitches where the ligand represents a distal structural linchpin for the switch helix.

## Methods

### RNA preparation and crystallization

RNA was cloned, transcribed, purified, refolded with 2.5 mM Mn^2+^, and screened for crystallization as previously described^12^. The *X. oryzae* aptamer domain sequence was modified to improve the chances of crystallization, including replacing the terminal loops in variable regions with GAAA tetraloops and adding a GG at the beginning of the sequence to increase T7 RNA polymerase efficiency^40^. We also removed a single unconserved U flip-out near the base of P1.1. In this construct, a native CA dinucleotide at the four-way junction was omitted from the sequence (Fig. 1a), possibly aiding crystallization. Initial crystal hits were obtained using the Nucleic Acid Mini-Screen (Hampton Research). This RNA crystallized at 0.2 mM in 10% MPD, 40 mM Na cacodylate (pH 7), 12 mM spermine tetrahydrochloride, 80 mM SrCl_2_, and 20 mM MgCl_2_ after 2–3 months at 18 °C.

### X-ray data collection and structure building

A 2.96 Å resolution X-ray diffraction dataset was collected at NE-CAT beamline 24-ID-C at the Advanced Photon Source (Argonne, IL), at 0.769 Å wavelength in order to use the Strontium K-edge for phasing. An additional dataset was collected at a remote wavelength (0.7749 Å) for SIRAS. Datasets were processed by XDS^41^ as part of NE-CAT’s RAPD pipeline. An initial solution was achieved by molecular replacement using the central region of the *L. lactis* structure^12^ followed by finding portions of external helices^42^. This partial solution was then used for MR-SAD in AutoSol in the Phenix suite^43,44^ to find Sr sites and obtain an initial electron density map. Since this dataset was collected near the Sr K-edge wavelength (0.769 Å), we could observe anomalous signal from Sr^2+^ ions in the crystal. At 4*σ* (a relatively low threshold), there was modest signal at the Conformer 2 M_A,Mg_ site, suggesting only partial occupancy by Sr^2+^ (Fig. 2b). However, there is no occupancy seen at any of the other M_A,Mg_ or M_B,Mn_ sites, despite there being external sites in the lattice with stronger anomalous signal (Supplementary Fig. 1). This suggests that the Conformer 2 M_B,Mn_ site, despite lacking several contacts, can still distinguish between metals. It possibly discriminates by geometry; the Sr^2+^ ionic radius is 2.4 Å, compared to the smaller Mn^2+^ (2.2 Å) or Mg^2+^ (2.1 Å). Mg^2+^ cannot be differentiated from Mn^2+^ at the wavelength and resolution of this structure. Alternating rounds of building in Coot^45^ and refinement with phenix.refine^46^ and intermittent re-phasing and density modification in AutoSol were used to build the final model (Table 1).

### Molecular dynamics simulations

The crystal structures of yybP-ykoY riboswitch from *X. oryzae* from this study (both Conformers 1 and 2) and from *L. lactis* (PDB ID 4Y1I^12^ and PDB ID 6CB3^19^) were used as starting structures for MD simulations.

We aimed to study the effect of divalents in the metal ion binding sites on the structural dynamics of the riboswitch, so we prepared a set of starting structures containing different ions (Mn^2+^, Mg^2+^, and K^+^) in the M_A,Mg_ and M_B,Mn_ sites (in case of the 6CB3 structure chain A, we considered also the ion in the third M_C_ ion binding site reported in this structure, see Supplementary Table 1). In addition, we probed both for syn- and anti-conformations of A48 in Conformer 2 from *Xory* to verify refinement of the corresponding electron density as a *syn*-oriented nucleotide. Starting structures of the SRL-like motif lacking tertiary interactions with the rest of the aptamer were prepared from the crystal structures but entailing only the P1.1, P1.2, and L1 segments without divalents.

All MD simulations were carried out using pmemd.cuda (GPU code of AMBER 14 program package)^47,48^ with the ff99bsc0χ_OL3_ force field^49–51^. The simulation protocol was as follows. The structure with the RNA molecule and any ions in the M_A,Mg_ and M_B,Mn_ (and in case of 6CB3 chain A even the M_C_) sites was immersed into a solvation box of SPC/E explicit water molecules^52^. We added KCl salt excess corresponding to 150 mM KCl. We used following ionic parameters: K^+^ (*r* = 1.593 Å, *ε* = 0.4297 kcal/mol^53^), Mg^2+^ (*r* = 1.5545 Å, *ε* = 0.00295 kcal/mol^54^), Mn^2+^ (*r* = 1.4060 Å, *ε* = 0.0167 kcal/mol^55^), and Cl^−^ (*r* = 2.711 Å, *ε* = 0.0127 kcal/mol^53^). Where indicated, we replaced one or both divalent ions in the M_A,Mg_ and M_B,Mn_ sites by K^+^ monovalents so that we probed for each specific starting structure four different ionic configurations in these ion binding sites (Supplementary Table 1). In the case of 6CB3 *L. lactis* structures we replaced the Cd^2+^ ions in the M_A,Mg_, M_B,Mn_, or M_C_ sites with K^+^ monovalents or Mg^2+^ and Mn^2+^ divalents in similar manner (Supplementary Table 1). The solvated systems were equilibrated as follows. Geometrical optimization of hydrogen atoms was followed by optimization of waters and ions, while the position of the RNA molecule remained restrained. Subsequently, all RNA atoms were restrained and the solvent molecules with counter-ions were allowed to move during a 500-ps long MD run under NpT conditions (*p* = 1 atm, *T* = 298.16 K) to relax the total density of solvent surrounding the RNA. Next, the RNA molecule was relaxed by several minimization runs, with decreasing force constant of position restraints applied to the sugar-phosphate backbone. After full relaxation, the system was heated in two steps: The first step involved heating under NVT conditions for 100 ps, whereas the second step involved final density equilibration under NpT conditions for an additional 100 ps. The particle-Mesh Ewald method for treating electrostatic interactions was used, and simulation were performed under periodic boundary conditions in the NVT ensemble at 298.16 K using a weak coupling Berendsen thermostat with a coupling time of 0.2 ps. The SHAKE algorithm, with a tolerance of 10^−5^ Å, was used to fix the positions of all hydrogen atoms, and a 10.0 Å cut-off was applied to nonbonding interactions. The hydrogen mass repartitioning was used to change the mass of hydrogen and neighboring atoms to allow a 4 fs integration step. In the production runs of the isolated SRL-like motif (P1.2, L1, and P1.1 segments only) we applied upper-wall positional restraints to the hydrogen bonds of the terminal base pair of the P1.2 stem (i.e., the G16 = C89 base pair proximal to the P2 stem in the complete structure) to mimic the stabilization effect of coaxial stacking of this base pair on the P2 stem. A parabolic restraint potential was applied at heavy atom distances of the particular hydrogen bonds above 3.5 Å with a force constant of 2.5 kcal/mol/Å^2^. The simulation time for each studied system was at least 2 µs (Supplementary Table 1).

### Data analysis

All trajectories were analyzed with the Ptraj module of the AMBER package^48^ and the simulations were visualized using VMD^56^. B-factors were calculated as average mass-weighted fluctuation of each residue over the entire trajectory aligned to the stem closest to the residue. This guarantees that the B-factors are not influenced by global motions and reflect local flexibilities only. The stacking interactions were detected using the G-vector of an eRMSD metric and were annotated using baRNAba^57^.

### RNA preparation for single-molecule FRET

The RNA for smFRET studies was annealed from two synthetic RNA oligonucleotides (Supplementary Fig. 23), ordered with the indicated modifications from IDT. The native CA dinucleotide omitted for crystallization was present in the smFRET RNA. Oligonucleotide 1 has a 5'-Cy5 and oligonucleotide 2 has 5'-Cy3 and 3' biotin-TEG. In this two-oligonucleotide design of the smFRET construct, the donor fluorophore Cy3 and biotin-TEG were placed on the 5' and 3' ends of stem P1.1 while the acceptor fluorophore Cy5 is attached to the 5' end of P3.2, positioning the fluorophores on either end of the distal arms that dock. For specifically probing the docking interaction, we extended the stem P1.1 to prevent end fraying. The biotin on the 3'-end of P1.1 was used for immobilization of the RNA onto a microscope quartz slide and subsequent prism-based total internal reflection fluorescence (TIRF) microscopy (Fig. 4a). Optimal refolding of the RNA was achieved with a mixture of 1 µM oligonucleotide 1 and 1.5 µM oligonucleotide 2 in 20 mM HEPES pH 7.0, 50 mM KCl. In PCR tubes, samples were heated to 70 °C for 2 min, then 2 mM MgCl_2_ was added, samples were allowed to cool to RT for 10 min, then put on ice until further use. To check assembly, ~1 pmol of each oligonucleotide (1–2 µL) was added to 10 µL running buffer supplemented with 10% glycerol and loaded onto a nondenaturing 8% polyacrylamide gel run at 4 °C. A buffer of 0.5× TBE supplemented with 2 mM Mg(OAc)_2_ and 50 mM KCl was used in the gel and as the running buffer.

### smFRET data acquisition and analysis

A small microfluidic channel was made by sandwiching a cleaned quartz slide and a glass coverslip using double-side tape. Beforehand, two holes were drilled into the quartz slide and afterwards tubing was attached to them to act as inlet and outlet ports that were used for flowing buffer and introducing the RNA. The two RNA oligonucleotides containing the Cy3, Cy5 and biotin modifications were mixed at a concentration of 2 µM each in 50 mM HEPES, pH 7.2, 100 mM KCl (1× buffer). The RNA was heated at 90 °C for 2 min, left at RT for 30 s to cool down, followed by addition of 2 mM MgCl_2_ and slow cooling down to RT over 15 min. This annealed RNA stock was used for carrying out smFRET experiments, for which a 15–25 pM RNA solution was made by diluting the 2 µM annealed stock using 1× buffer.

Totally, 100 µL of diluted RNA solution was flowed onto the quartz slide coated with biotinylated-bovine serum albumin and streptavidin for immobilization and incubated for 2 min. Any unbound RNA was washed off using 1× buffer. This step also washes off small (low nM) amounts of residual Mg^2+^ present in the stock. For experiments in the absence of divalents, 0.1 mM EDTA was included in the 1× buffer to chelate any contaminating divalent ions. Mg^2+^ titration experiments were performed on the same slide after washing off the EDTA using 1× buffer. We included an enzymatic oxygen scavenging system (OSS) containing 50 nM protocatechuate-3,4-dioxygenase and 5 mM protocatechuic acid in 1× buffer to prolong the longevity of the fluorophores. In addition, 2 mM Trolox (6-Hydroxy-2,5,7,8-tetramethylchromane-2-carboxylic acid) was included in the imaging buffer to suppress photoblinking of the dyes.

All smFRET movies were acquired at ~16 Hz, unless otherwise specified, using a prism-based TIRF microscope with an intensified CCD (I-Pentamax, Princeton Instruments) or sCMOS camera (Hamamatsu ORCA-Flash4.0 V3), essentially as previously described^58,59^. Cy3 on the RNA was excited using a 532 nm laser and the emission from both Cy3 (donor) and Cy5 (acceptor) were simultaneously detected side-by-side on the camera. Toward the end of all movies, the Cy5 was directly excited using a 640 nm laser to check for the presence of the acceptor (Cy5) fluorophore. This helps in distinguishing the low-FRET (~0.1 FRET) states we observe from ~0 FRET states due to the absence or photobleaching of Cy5 in Cy3-labeled molecules. Raw movies were analyzed using IDL (Research Systems) to extract the time traces for all spots in Cy3 and Cy5 channels. Single molecule traces were then visualized using Matlab and only those with a minimum combined intensity (Cy3 + Cy5 intensity) of 300, showing single-step photobleaching of the dyes, a signal-to-noise ratio of >3, and longer than 6 s were selected for further analysis. Selected traces were then background-subtracted to correct for cross-talk and (minimal) bleed-through. We calculated the FRET ratio as *I*_*A*_/(*I*_*A*_ + *I*_*D*_), where *I*_*A*_ and *I*_*D*_ are the background corrected intensities of acceptor (Cy5) and donor (Cy3), respectively. FRET histograms were made using the first 50 frames (3 s) of all traces (at least 200) in a given condition and fit with a sum of Gaussians using OriginPro 8.5. For kinetic analysis, traces were idealized with a two-state model corresponding to undocked (low-FRET) and docked (high-FRET) states using the segmental k-means algorithm in QuB software as previously described^26,60^. Cumulative dwell-time histograms were plotted from all extracted dwell times and fit with single- or double-exponential functions using OriginPro 8.5 to obtain the lifetimes in the undocked (*τ*_undock_) and docked (*τ*_dock_) states. Rate constants of docking and undocking were then calculated as *k*_dock_ = 1/*τ*_undock_ and *k*_undock_ = 1/*τ*_dock_. For the double-exponential fits, kinetics were calculated similarly using both the short and long dwell lifetimes to obtain the fast and slow rate constants, respectively. The idealized smFRET traces were used for creating TODPs, which show the fraction of traces/molecules that exhibit a given type of transition at least once^26^. In TODPs, dynamic traces showing a FRET transition (regardless of the number of transitions in that trace) and static traces (with no transitions over the entire trace) are weighted equally, avoiding over-representation of the traces with fast transitions. This feature is distinct from traditional transition density plots that show all FRET transitions in a population as off-diagonal contours in the heat map, which leads to the visual dominance of rapid, frequent transitions. By contrast, TODPs are meant to show the fractions of different kinds of molecular behaviors and highlight slow or rare transitions found to be representing a large subpopulation of traces. In addition, the TODPs shown here also include static traces that appear as on-diagonal contours and are often critical in understanding the molecular mechanisms of riboswitches^61,62^. The on-diagonal contours in these TODPs (at ~0.2 and ~0.7 FRET values) thus do not represent any transitions, but rather highlight static traces that lack any FRET transitions over their entire observation length and are counted exactly once in the TODP heatmap.

### SiM-KARTS assay

The SiM-KARTS assay to probe P1.1 accessibility was performed on a prism-based TIRF microscope^36^. Totally, 20 pM of the riboswitch was bound to the slide and the Cy5 on the riboswitch was first photobleached by excitation with a red laser. Kinetic measurements of P1.1 accessibility was performed in the presence of 10 nM of a Cy5-labeled DNA oligonucleotide and under varying concentrations of the metal ions in the 1× buffer with OSS also used for smFRET. The 5' Cy5-labeled DNA oligonucleotide is complementary to the 12-nt sequence (5'-GAGACCAGGGAU-3') of the 3'-half of L1 and P1.1 in the smFRET RNA construct (Supplementary Fig. 23). Movies were acquired at 100 ms time resolution under dual excitation with both a green and red laser to localize the Cy3-labeled surface-bound riboswitch molecules and to image the short binding events of the Cy5-labeled oligonucleotide probe, respectively. The Cy5 versus intensity time traces were idealized to a two-state model using the segmental *k*-means algorithm implemented in QuB to obtain the bound and unbound dwell times. The unbound and bound lifetimes were obtained by single-exponential fits to the cumulative dwell time distributions, the inverse of which gives the rate constants *k*_on_ and *k*_off_, respectively. The errors of the rate constants were estimated using a boot-strapping algorithm by taking three random subsets from all the traces in a condition and calculating the rate constants. The means and standard deviations from the three calculated rates are reported in Fig. 7.

### Reporting summary

Further information on research design is available in the Nature Research Reporting Summary linked to this article.

## Supplementary information

Supplementary Information

Peer Review

Reporting Summary

Source Data

## Data Availability

A reporting summary for this Article is available as a Supplementary Information file. Atomic coordinates and structure factors for the reported crystal structure have been deposited with the Protein Data Bank under accession number 6N2V. The source data underlying Figs. 4e, h and 7c, e are provided as a Source Data file. The data supporting the findings of this study are available from the corresponding authors upon reasonable request.

## Code availability

Custom scripts used to analyze the smFRET and MD simulations data can be made available upon request.
