# Supplementary material for: Local-to-global signal transduction at the core of a Mn2+ sensing riboswitch
Source: Nat Commun. 2019 Sep 20;10:4304. doi: 10.1038/s41467-019-12230-5 (PMC6754395; doi:10.1038/s41467-019-12230-5)
Supplement: Supplementary file 1 — Supplementary Information [file 41467_2019_12230_MOESM1_ESM.docx]

Supplementary Information for

**Local-to-global signal transduction at the core of a Mn^2+^ sensing riboswitch**

Krishna C. Suddala^1^*, Ian R. Price^2^*, Shiba S. Dandpat^1^, Michal Janeček^3,5^, Petra Kührová^4,5^, Jiří Šponer^3,4^, Pavel Banáš^3,4,5^, Ailong Ke^2^, Nils G. Walter^1^

*These authors contributed equally

Correspondence and requests for materials should be addressed to A.K. (email: ailong.ke@cornell.edu) or to N.G.W. (email: nwalter@umich.edu)

**Supplementary Figure 1**

**Supplementary Figure 1|** *Xory* conformers 1 (orange) and 2 (cyan) with 2Fo-Fc electron density maps at the 1.5 sigma level.

.

**Supplementary Figure 2**


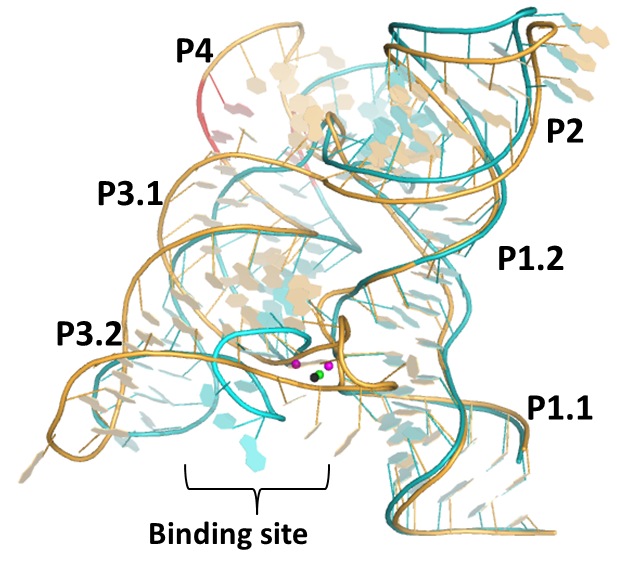

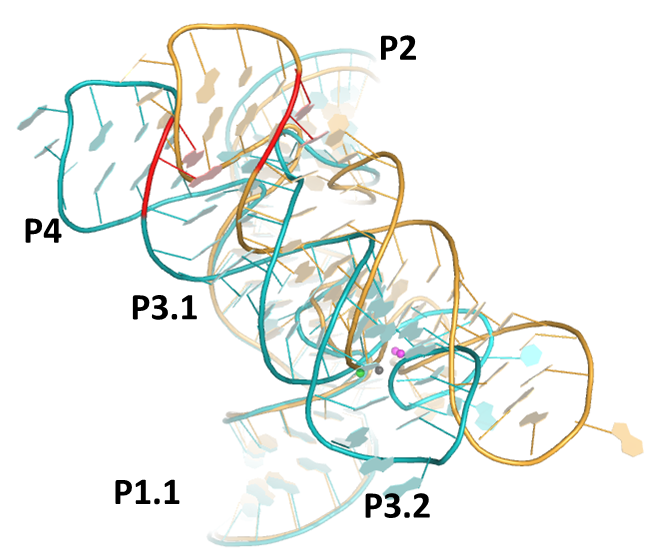


**Supplementary Figure 2| Overall comparison of Conformers 1 and 2**. When aligned by L1, there is also a shift of the P3/P4 helical stack between Conformers 1 (orange) and 2 (cyan). The *L. lactis* structure (not shown) is intermediate but more similar to Conformer 1 when aligned this way.

**Supplementary Figure 3**


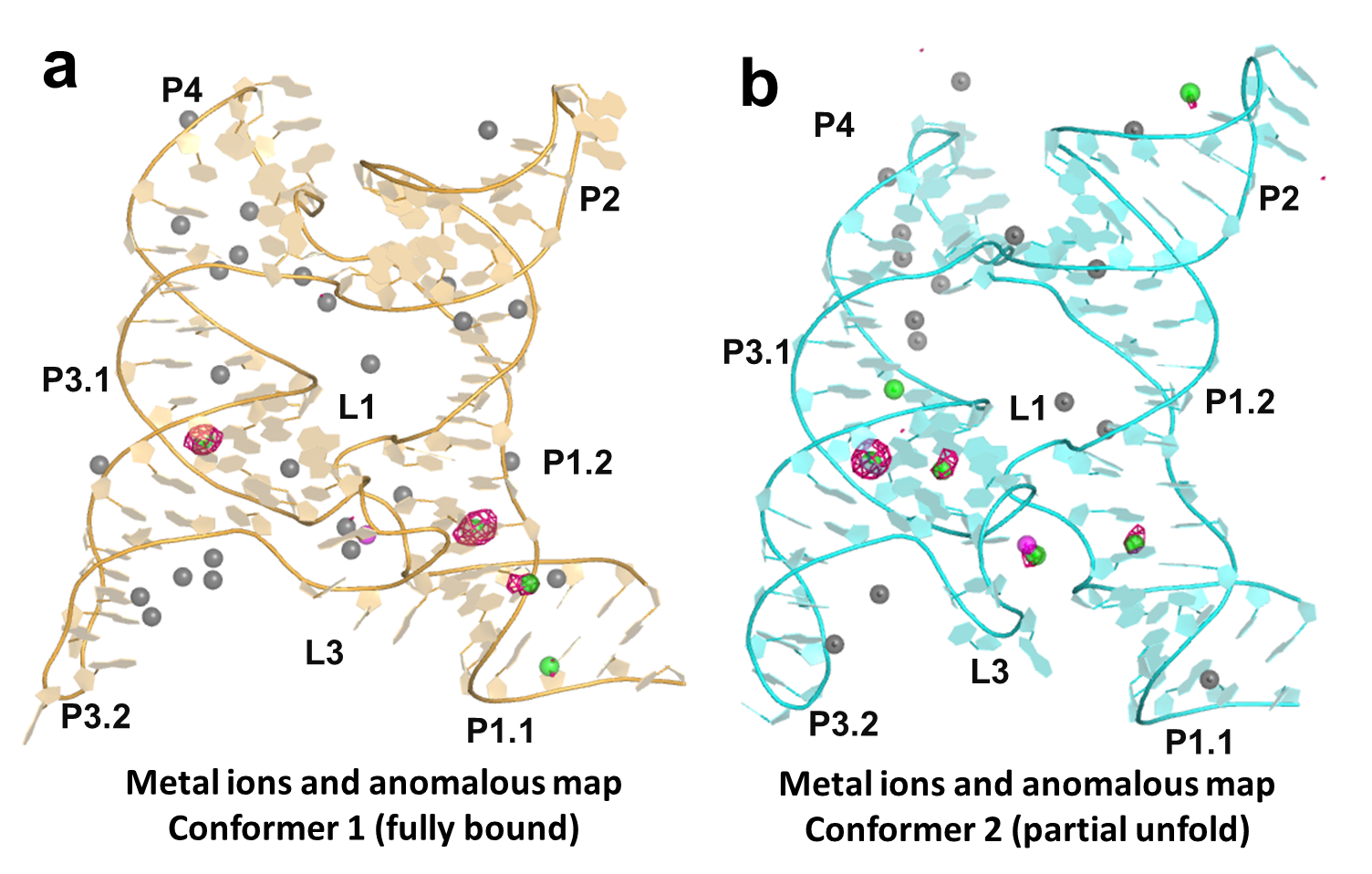


**Supplementary Figure 3| Overall ions and anomalous sites for the *X. oryzae* *yybP-ykoY* riboswitch crystal construct**. **a)** Conformer 1 (orange) and **b)** Conformer 2 (cyan). Total ions within 5 Å of A chain include 2 Mn^2+^ (magenta), 45 Mg^2+^ (or Mn^2+^) (black), and 9 Sr^2+^ (green). The anomalous difference map, collected at 0.769 Å, is shown in pink mesh at level 4 σ. Placement of Sr^2+^ ions was determined by anomalous map and/or high electron density. Since Mn^2+^ has minimal anomalous signal at this wavelength and it is similar in ionic radius to Mg^2+^, it could not be differentiated from Mg^2+^. Thus, Mn^2+^ (2.5 mM in the crystal) could partially or fully occupy sites denoted as Mg^2+^ (30 mM in the crystal). Binding site Mn^2+^ were predicted to be so based on previous analysis of the *L. lactis* structure, but were not confirmed by this structure.

**Supplementary Figure 4**

**Supplementary Figure 4|** 2F_o_-F_c_ electron density maps of the Mn^2+^-binding regions of conformers 1 **(a)** and 2 **(b)**. Maps (gray surfaces) are shown at 1.5 and 1.3 sigma level, respectively

**Supplementary Figure 5**

**Supplementary Figure 5**| **Metal binding sites in the *L. lactis yybP-ykoY* structure.** Close-up view of the M_B,Mn_ (purple) and M_A,Mg_ (black) metal binding sites showing different contacts with surrounding L3 and L1 residues. The oxygen atoms of water molecules are shown as red spheres.

**Supplementary**
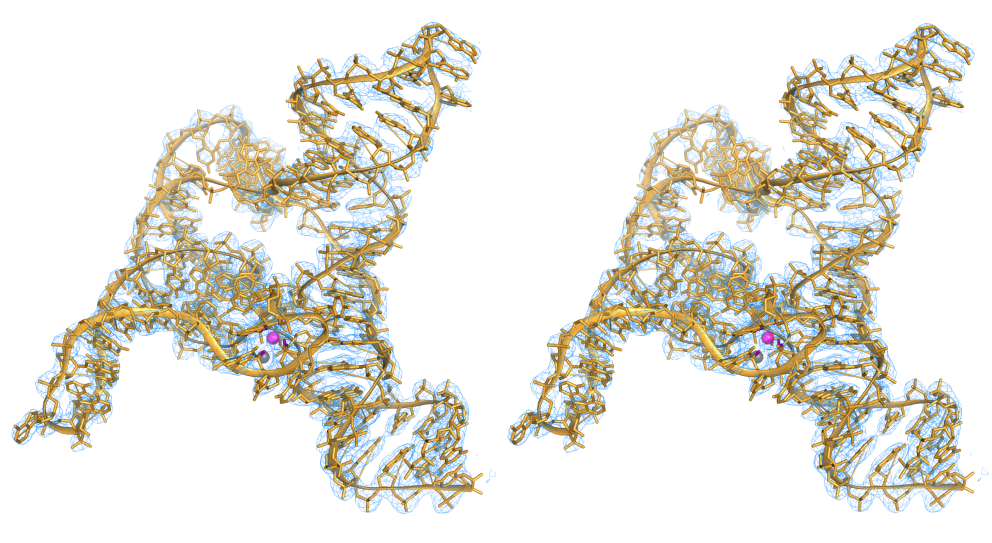
**Figure 6**

A


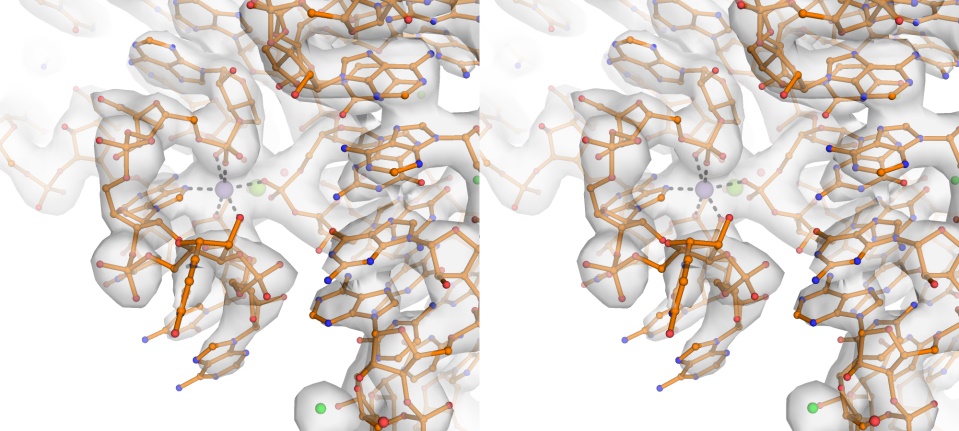


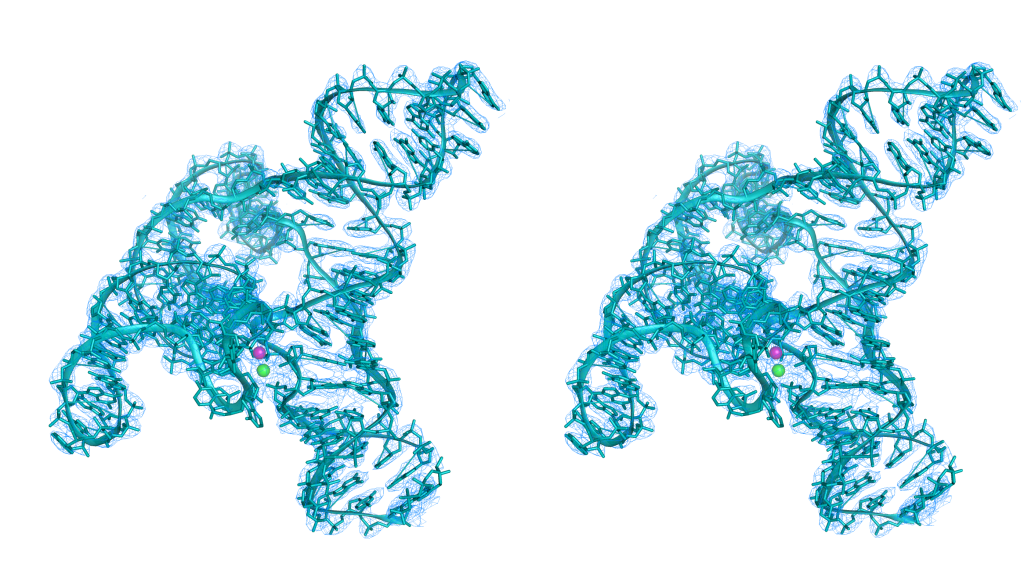


B

C


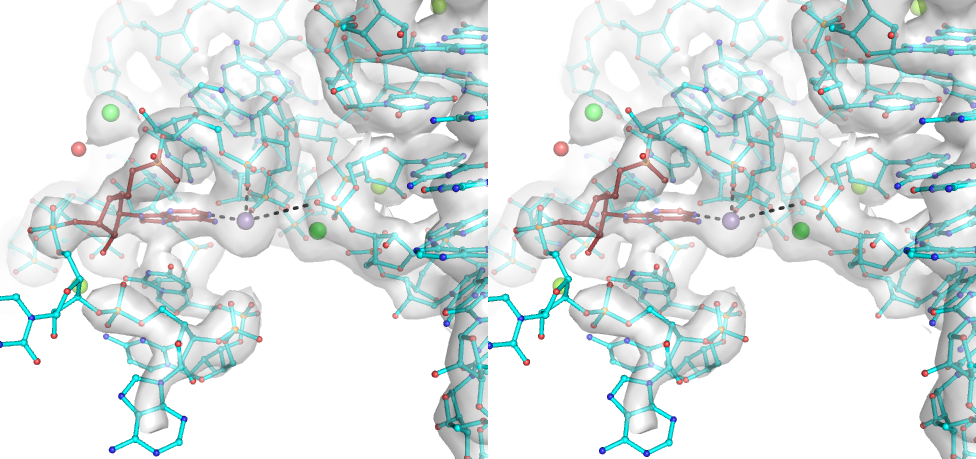
D

**Supplementary Figure 6**| **Stereoscopic images of the X*. oryzae* riboswitch structure.** The overall structures of conformers 1 (A) and 2 (B) are shown. Closer images of the Mn2+-binding regions for conformers 1 and 2 are shown in C and D, respectively. 2F_O_-F_C_ electron density maps are shown on all at 1.5-sigma level.

**Supplementary Table 1**. List of 43 MD simulations comprising a cumulative 113 µs simulation time

| Organism | PDB ID | Structure | Chain | Ions in active site | | | solvent box | Length |
| --- | --- | --- | --- | --- | --- | --- | --- | --- |
|  |  |  |  | M_A,Mg_ | M_B,Mn_ | M_C_ |  |  |
| *L. lactis* | 4Y1I | complete | A | Mg^2+^ | Mn^2+^ | - | rect. | Each  3 µs |
|  |  |  |  | Mg^2+^ | K^+^ | - |  |  |
|  |  |  |  | K^+^ | Mn^2+^ | - |  |  |
|  |  |  |  | K^+^ | K^+^ | - |  |  |
|  |  | P1.2\|L1\|P1.1 | A | - | - | - | rect. | 3 µs |
|  | 6CB3 | complete | A with canonical U44 | Mg^2+^ | Mn^2+^ | Mg^2+^ | rect. | Each  3 µs |
|  |  |  |  | Mg^2+^ | K^+^ | Mg^2+^ |  |  |
|  |  |  |  | K^+^ | Mn^2+^ | Mg^2+^ |  |  |
|  |  |  |  | K^+^ | K^+^ | Mg^2+^ |  |  |
|  |  |  |  | Mg^2+^ | Mn^2+^ | K^+^ |  |  |
|  |  |  |  | Mg^2+^ | K^+^ | K^+^ |  |  |
|  |  |  |  | K^+^ | Mn^2+^ | K^+^ |  |  |
|  |  |  |  | K^+^ | K^+^ | K^+^ |  |  |
|  |  |  | A with deprotonated U44^-^ | Mg^2+^ | Mn^2+^ | Mg^2+^ |  |  |
|  |  |  |  | Mg^2+^ | K^+^ | Mg^2+^ |  |  |
|  |  |  |  | K^+^ | Mn^2+^ | Mg^2+^ |  |  |
|  |  |  |  | K^+^ | K^+^ | Mg^2+^ |  |  |
|  |  |  |  | Mg^2+^ | Mn^2+^ | K^+^ |  |  |
|  |  |  |  | Mg^2+^ | K^+^ | K^+^ |  |  |
|  |  |  |  | K^+^ | Mn^2+^ | K^+^ |  |  |
|  |  |  |  | K^+^ | K^+^ | K^+^ |  |  |
|  |  |  | B | Mg^2+^ | Mn^2+^ | - |  |  |
|  |  |  |  | Mg^2+^ | K^+^ | - |  |  |
|  |  |  |  | K^+^ | Mn^2+^ | - |  |  |
|  |  |  |  | K^+^ | K^+^ | - |  |  |
| *X. oryzae* | This study | complete | Conformer 2 with  *anti* A48 | Mg^2+^ | Mn^2+^ | - | oct. | Each  2 µs |
|  |  |  |  | Mg^2+^ | K^+^ | - |  |  |
|  |  |  |  | K^+^ | Mn^2+^ | - |  |  |
|  |  |  |  | K^+^ | K^+^ | - |  |  |
|  |  |  | Conformer 2 with  *syn* A48 | Mg^2+^ | Mn^2+^ | - |  |  |
|  |  |  |  | Mg^2+^ | K^+^ | - |  |  |
|  |  |  |  | K^+^ | Mn^2+^ | - |  |  |
|  |  |  |  | K^+^ | K^+^ | - |  |  |
|  |  | P1.2\|L1\|P1.1 | Conformer 2 | - | - | - | rect. | 3 µs |
|  |  | complete | Conformer 1 | Mg^2+^ | Mn^2+^ | - | oct. | Each twice  2 µs |
|  |  |  |  | Mg^2+^ | K^+^ | - |  |  |
|  |  |  |  | K^+^ | Mn^2+^ | - |  |  |
|  |  |  |  | K^+^ | K^+^ | - |  |  |
|  |  | P1.2\|L1\|P1.1 | Conformer 1 | - | - | - | rect. | 3 µs |

**Supplementary Figure 7**


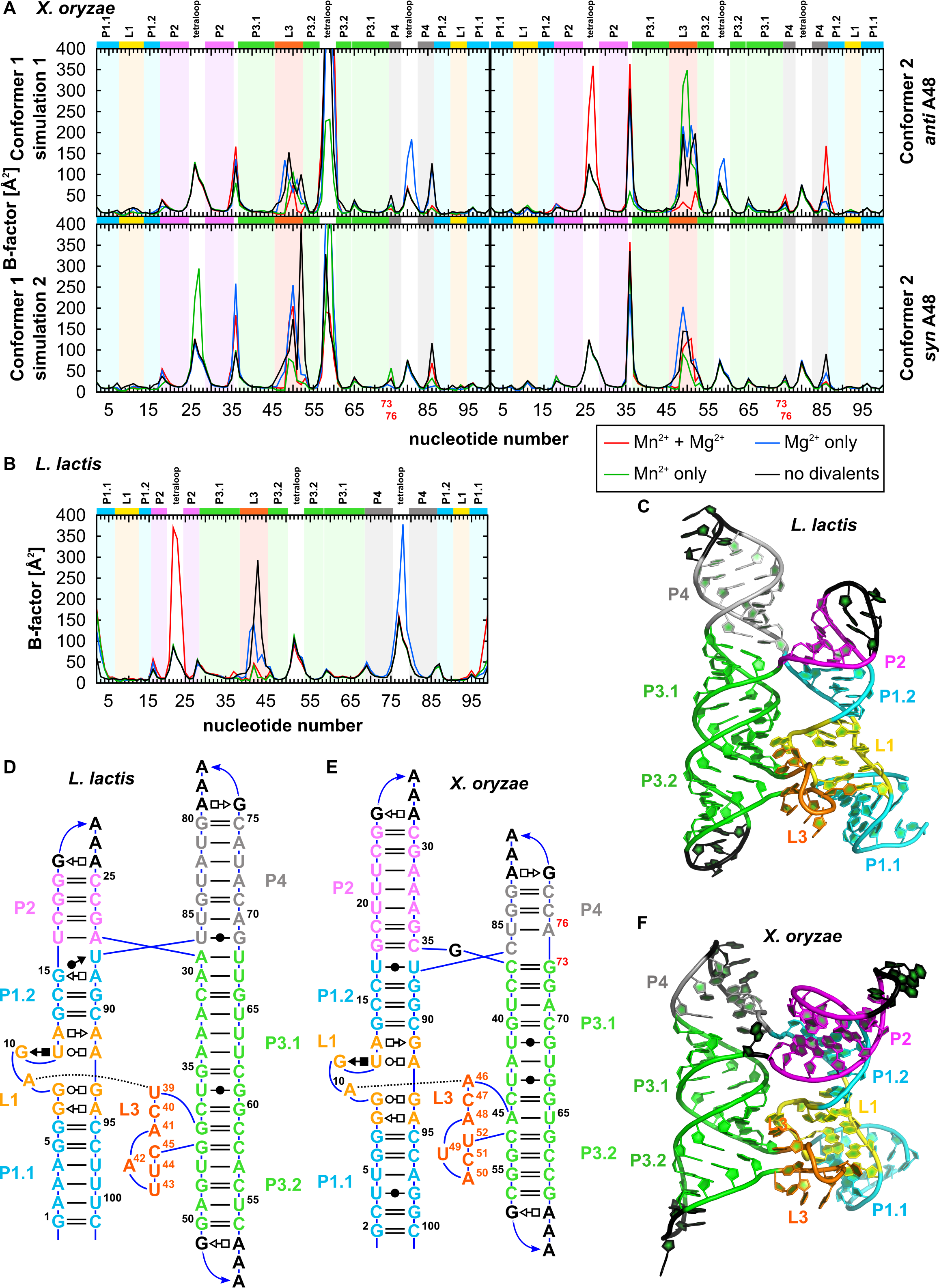


**Supplementary Figure 7**| The average mass-weighted B-factor as a function of residue number obtained in MD simulations of Mn^2+^ sensing riboswitch from A) *Xanthomonas oryzae* or B) *Lactococcus lactis* (see **Supplementary Table 1** in the main text for full list of the simulations). The colors of background bars correspond to the regions in the secondary and 3D structures shown on panels C-F.

**Supplementary Figure 8**


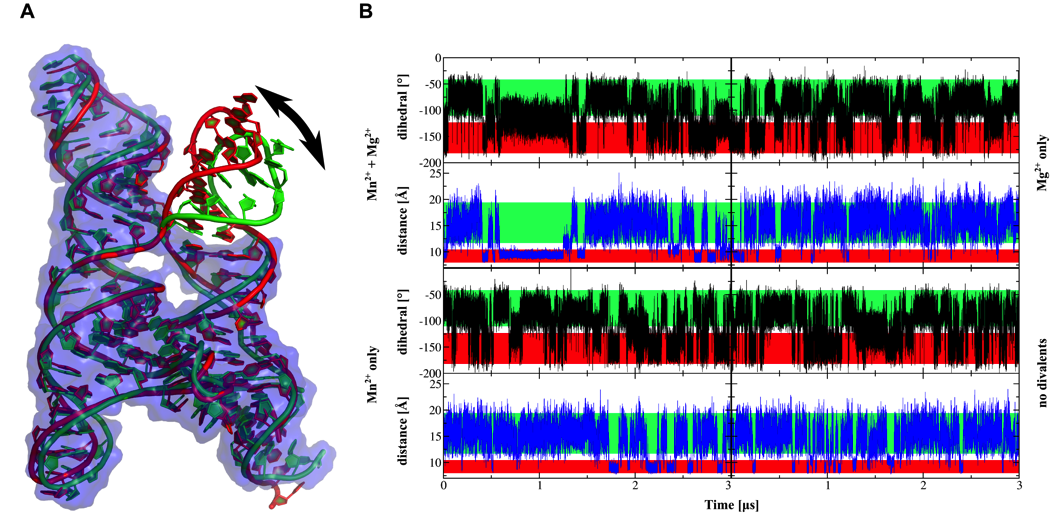


**Supplementary Figure 8**| A) Superposition of the open and closed conformation (in green and red, respectively) observed in MD simulation of the aptamer from *L. lactis*. B) Time dependence of ε dihedral angle of A28 (black line) and distance between P2 (center of mass of C17-G20 backbone) and P4 (center of mass of C71-A72 backbone) stems (blue line). The green and the red strips on the background correspond to the open (native crystal-like) and closed conformation, respectively.

**Supplementary Figure 9**


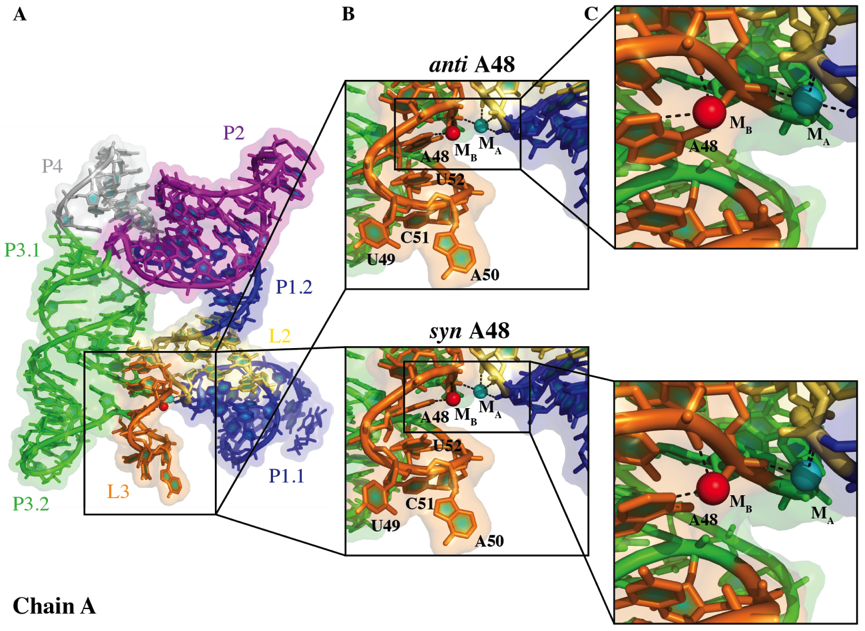


**Supplementary Figure 9|** Structure of *X. oryzae* aptamer domain of conformer 2 showing alternative *syn* and *anti*-orientations of A48.

**Supplementary Figure 10**


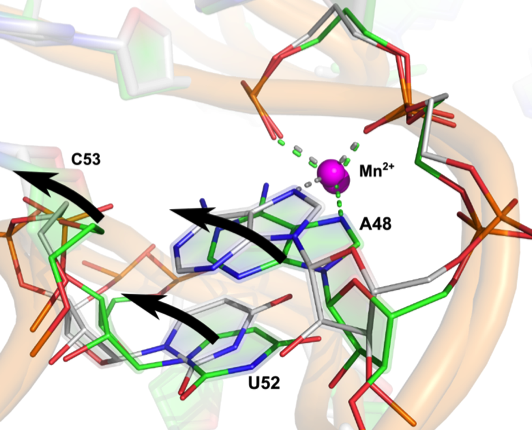


**Supplementary Figure 10|** Structural changes observed during early stages of the MD simulations of *X. oryzae* conformer 2 with *anti*-orientation of A48. Green structure corresponds to crystal structure, while the silver refers to the structure after initial rearrangements during these early stages.

**Supplementary Figure 11**


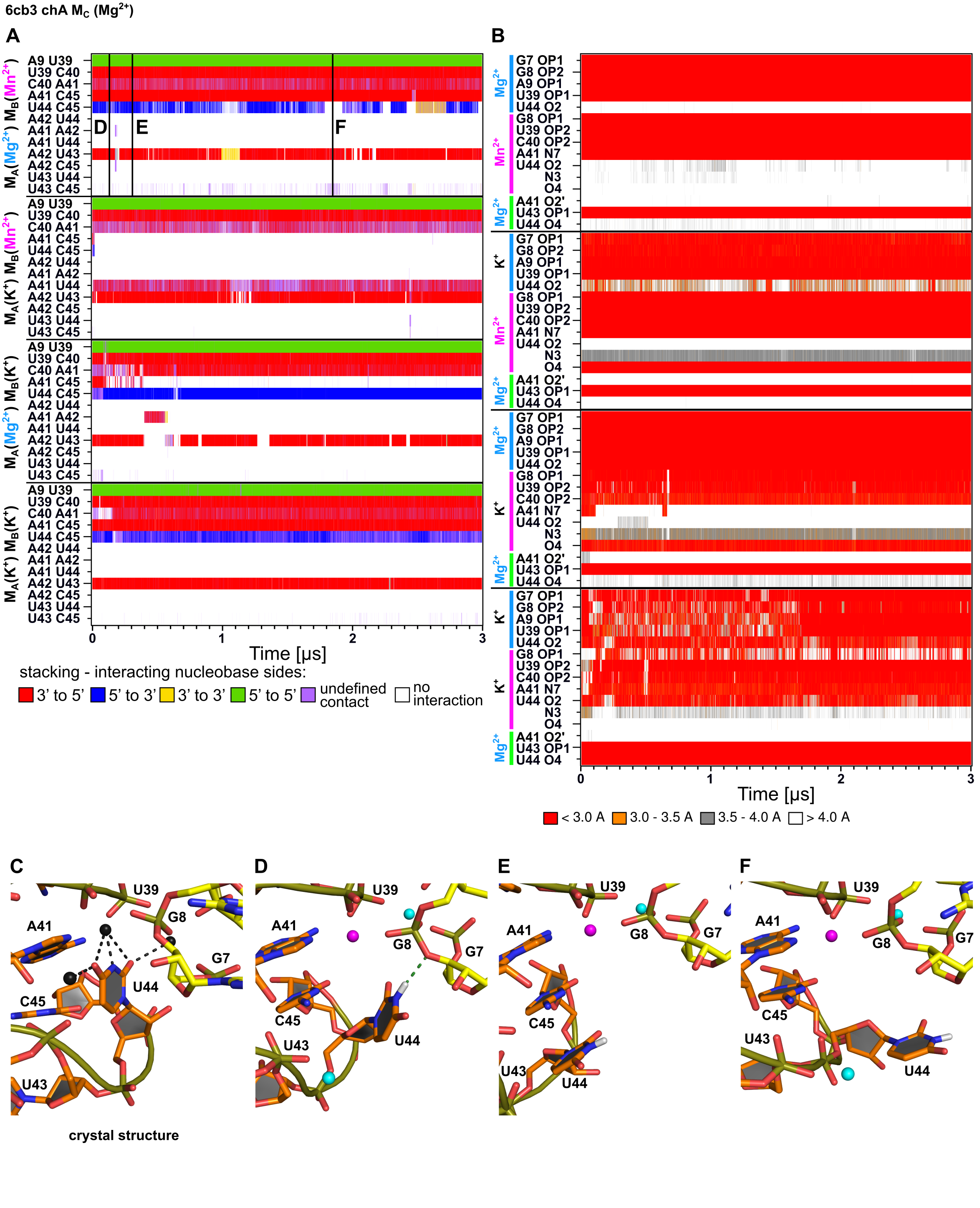


**Supplementary Figure** **11|** Conformational behavior of the L3 loop and the M_A_, M_B_ and M_C_ ion binding sites in MD simulations of the *L. lactis* riboswitch starting from the chain A of the 6CB3 structure with the M_C_ site occupied by a Mg^2+^ ion. (**A**) Time evolution of the stacking pattern of the loop; the colors correspond to different mutual orientations of nucleobases in stacking interactions indicated by the corresponding faces (3'-face and 5'-face) involved in the interaction. (**B**) Time evolution of ligand-ion interactions in the ion binding sites. (**C**) Unusual arrangement of the ion binding sites in the crystal structure involving three Cd^2+^ ions. (**D-F**) Snapshots from the MD simulations (indicated by black vertical lines in panel A), documenting different conformations that the canonical U44 sampled after its ejection from the ion binding site.

**Supplementary Figure 12**


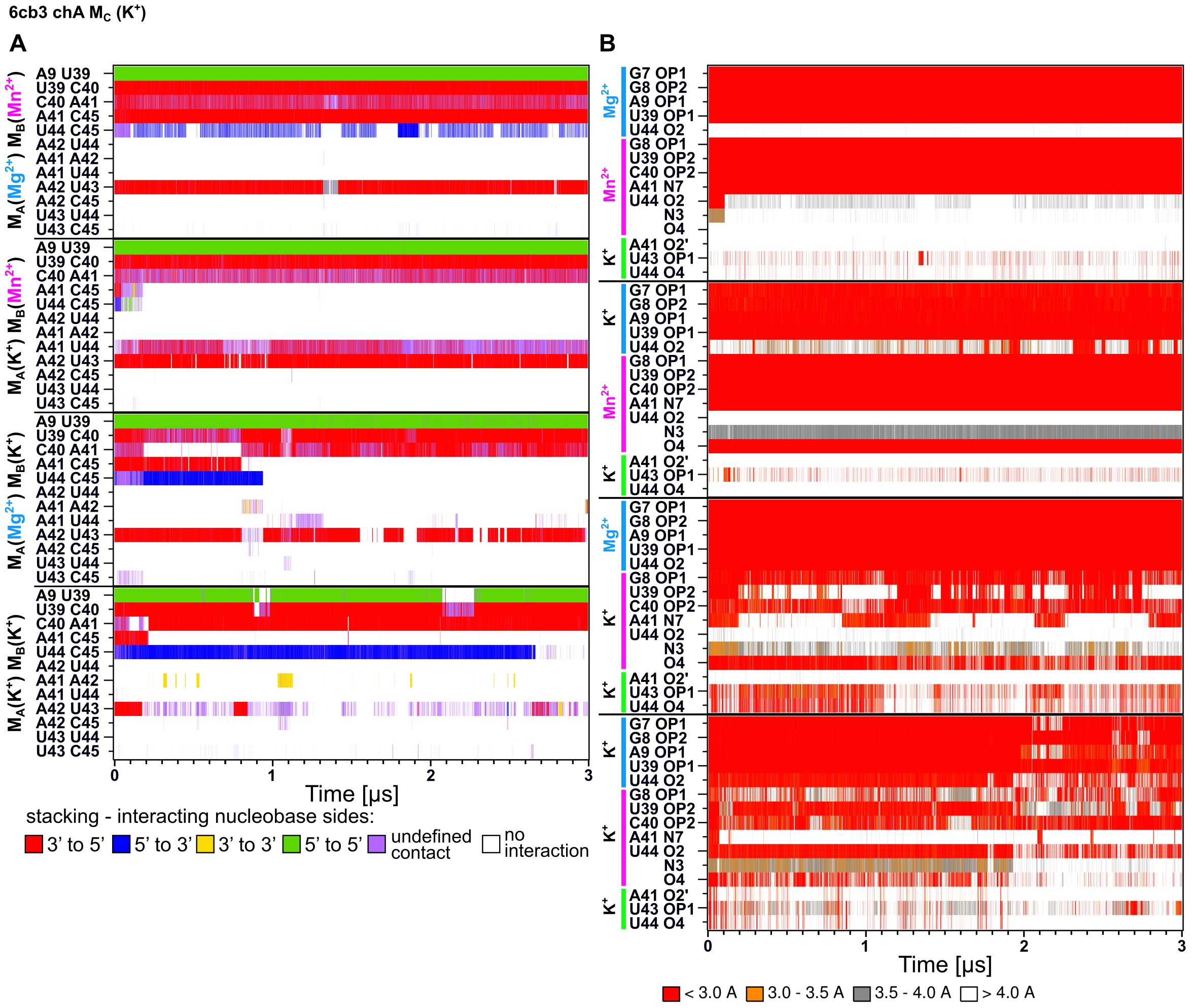


**Supplementary Figure 12|** Conformational behavior of the L3 loop and the M_A_, M_B_ and M_C_ ion binding sites in MD simulations of the *L. lactis* riboswitch starting from chain A of the 6CB3 structure with the M_C_ site occupied by a K^+^ ion. (**A**) Time evolution of the stacking pattern of the loop; the colors correspond to different mutual orientations of nucleobases in stacking interactions indicated by the corresponding faces (3'-face and 5'-face) involved in the interaction. (**B**) Time evolution of ligand-ion interactions in the ion binding sites.

**Supplementary Figure 13**

**
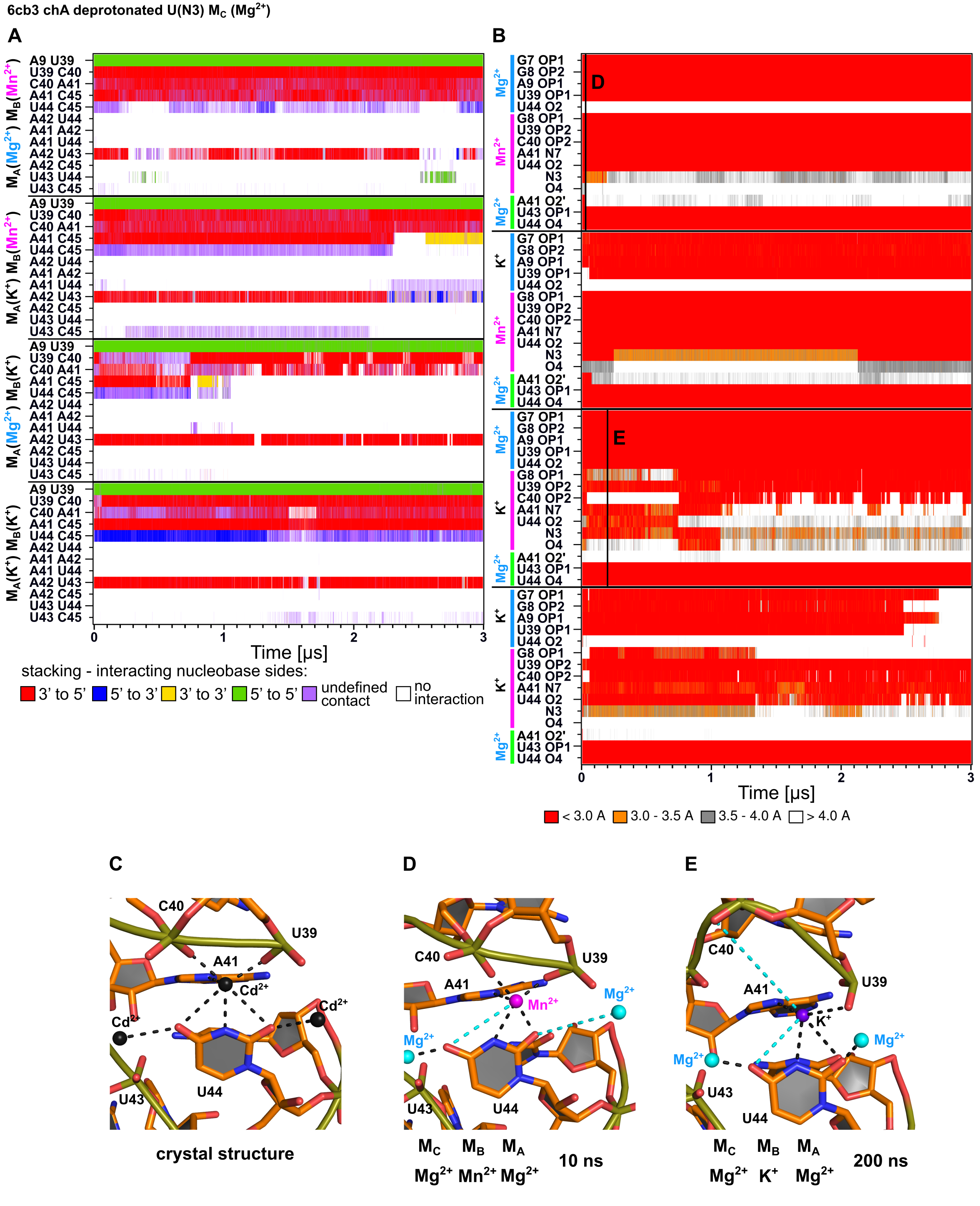
**

**Supplementary Figure** **13|** Conformational behavior of the L3 loop and the M_A_, M_B_ and M_C_ ion binding sites in MD simulations of the *L. lactis* riboswitch starting from chain A of the 6CB3 structure with the M_C_ site occupied by a Mg^2+^ ion and with an N3-deprotonated U44. (**A**) Time evolution of the stacking pattern of the loop; the colors correspond to different mutual orientations of nucleobases in stacking interactions indicated by the corresponding faces (3'-face and 5'-face) involved in the interaction. (**B**) Time evolution of ligand-ion interactions in the ion binding sites. (**C**) Unusual arrangement of the ion binding sites in the crystal structure involving three Cd^2+^ ions. (**D-E**) Snapshots from the MD simulations (indicated by black vertical lines in panel B) documenting modest shifts of N3-deprotonated U44^-^ within the binding site as observed in MD simulations. The stable and broken contacts are depicted by black and cyan dashed lines, respectively.

**Supplementary Figure 14**

**
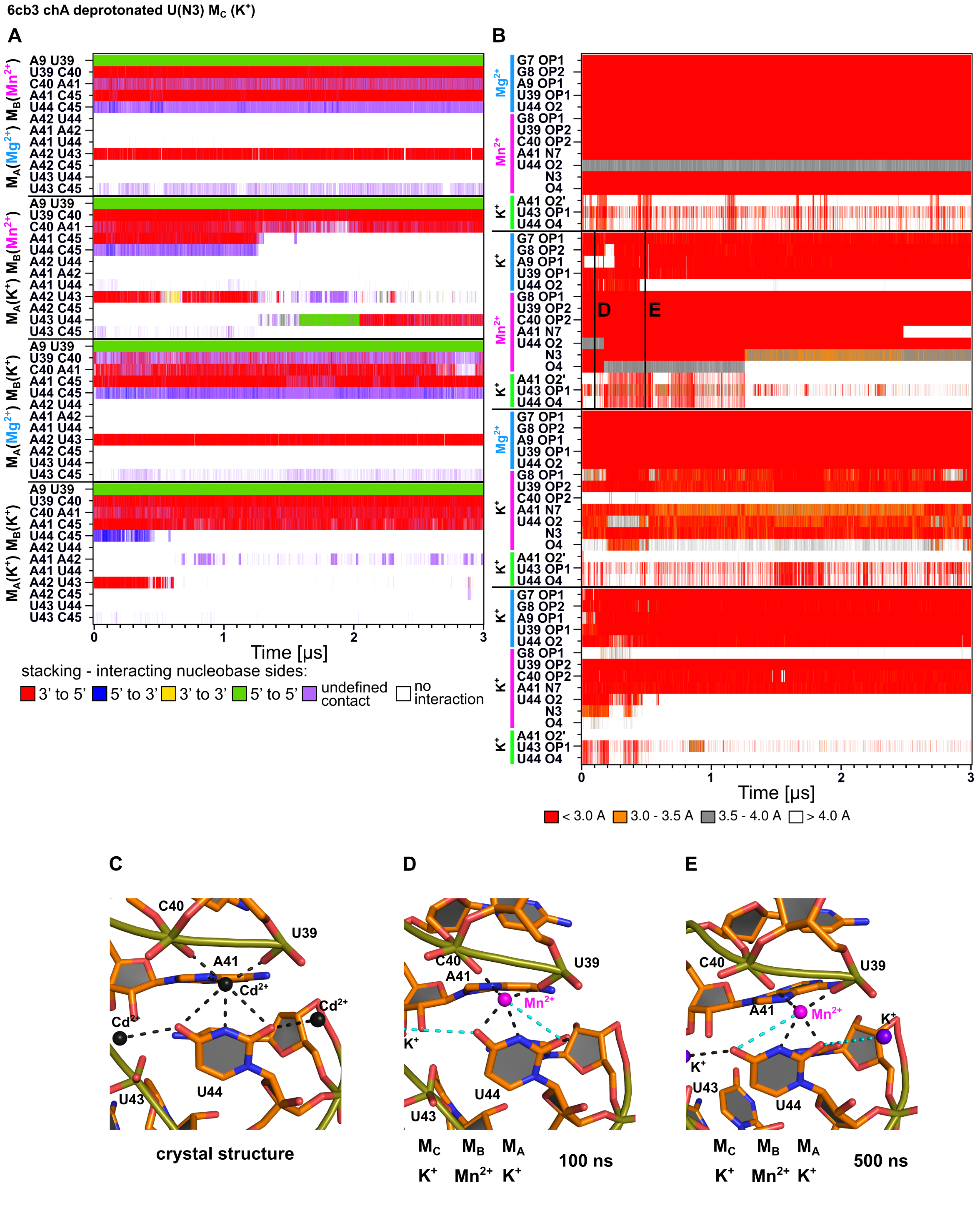
**

**Supplementary Figure** **14|** Conformational behavior of the L3 loop and the M_A_, M_B_ and M_C_ ion binding sites in MD simulation of the *L. lactis* riboswitch starting from chain A of the 6CB3 structure with the M_C_ site occupied by a K^+^ ion and with an N3-deprotonated U44. (**A**) Time evolution of the stacking pattern of the loop; the colors correspond to different mutual orientations of nucleobases in stacking interactions indicated by the corresponding faces (3'-face and 5'-face) involved in the interaction. (**B**) Time evolution of ligand-ion interactions in the ion binding sites. (**C**) Unusual arrangement of the ion binding sites in the crystal structure involving three Cd^2+^ ions. (**D-E**) Snapshots from the MD simulations (indicated by black vertical lines in panel B) documenting modest shifts of the N3-deprotonated U44^-^ within the binding site as observed in MD simulations. The stable and broken contacts are depicted by black and cyan dashed lines, respectively.

**Supplementary Table 2:** Percentage of native stacking preserved in the L3 loop during MD simulation (based on annotations calculated by the baRNAba software^1^) and a percentage of ion occupancy in the ion binding sites. 4Y1I_chA and 6CB3_chB correspond to simulations of the *L. lactis* riboswitch starting from the 4Y1I chain A and 6CB3 chain B structures, respectively. Xory_cf1_1, X.ory_cf1_2 and X.ory_cf2_syn signify two independent simulations of *X. oryzae* Conformer 1 structure and one simulation of Conformer 2 with *syn*-oriented A48, respectively (see **Supplementary Table 1**).

|  | **Ions** | | **Stacking interactions**  **[%]** | | | | | | **Occupancy of M_A_**  **[%]** | | | | | **Occupancy of M_B_**  **[%]** | | | | | |
| --- | --- | --- | --- | --- | --- | --- | --- | --- | --- | --- | --- | --- | --- | --- | --- | --- | --- | --- | --- |
|  | **M_A_** | **M_B_** | **A9 U39** | **U39 C40** | **C40 A41** | **A41 C45** | **U44 C45** | **A42 U44** | **G7 OP1** | **G8 OP2** | **A9 OP1** | **U39 OP1** | **C45 OP1** | **G8 OP1** | **U39 OP2** | **C40 OP2** | **A41 N7** | **U44 OP1** | **C45 OP2** |
| 4Y1I_chA | Mg^2+^ | Mn^2+^ | 100 | 98 | 77 | 98 | 92 | 76 | 100 | 100 | 100 | 100 | 100 | 100 | 100 | 100 | 100 | 100 | 100 |
|  | K^+^ | Mn^2+^ | 99 | 99 | 78 | 96 | 65 | 83 | 100 | 100 | 100 | 100 | 2 | 100 | 100 | 100 | 100 | 100 | 100 |
|  | Mg^2+^ | K^+^ | 99 | 99 | 2 | 4 | 5 | 6 | 100 | 100 | 100 | 100 | 100 | 100 | 100 | 98 | 6 | 0 | 100 |
|  | K^+^ | K^+^ | 100 | 93 | 64 | 88 | 36 | 42 | 95 | 98 | 95 | 100 | 0 | 27 | 99 | 78 | 97 | 42 | 67 |
| 6CB3_chB | Mg^2+^ | Mn^2+^ | 100 | 98 | 78 | 98 | 94 | 58 | 100 | 100 | 100 | 100 | 100 | 100 | 100 | 100 | 100 | 100 | 100 |
|  | K^+^ | Mn^2+^ | 98 | 100 | 81 | 96 | 66 | 92 | 100 | 100 | 100 | 100 | 2 | 100 | 100 | 100 | 100 | 100 | 100 |
|  | Mg^2+^ | K^+^ | 99 | 53 | 43 | 20 | 1 | 0 | 100 | 100 | 100 | 100 | 100 | 99 | 100 | 30 | 100 | 0 | 99 |
|  | K^+^ | K^+^ | 99 | 98 | 58 | 97 | 23 | 37 | 98 | 99 | 98 | 100 | 0 | 52 | 100 | 95 | 97 | 52 | 64 |
|  |  |  | **A10 A46** | **A46 C47** | **C47 A48** | **A48 U52** | **C51 U52** | **A50 C51** | **G8 OP2** | **G9 OP2** | **A10 OP1** | **A46 OP1** | **U52 OP1** | **G9 OP1** | **A46 OP2** | **C47 OP2** | **A48 N7/1** | **C51 OP1** | **U52 OP2** |
| *X.ory*_cf2_*syn* | Mg^2+^ | Mn^2+^ | 100 | 100 | 100 | 66 | 61 | 63 | 100 | 100 | 100 | 100 | 0 | 0 | 100 | 100 | 100 | 0 | 0 |
|  | K^+^ | Mn^2+^ | 91 | 100 | 100 | 81 | 86 | 89 | 99 | 99 | 100 | 100 | 0 | 0 | 100 | 100 | 100 | 0 | 0 |
|  | Mg^2+^ | K^+^ | 100 | 99 | 87 | 69 | 82 | 89 | 100 | 100 | 100 | 100 | 0 | 65 | 68 | 67 | 68 | 17 | 17 |
|  | K^+^ | K^+^ | 96 | 99 | 89 | 70 | 90 | 67 | 95 | 98 | 98 | 98 | 45 | 6 | 90 | 67 | 68 | 10 | 67 |
| *X.ory*_cf1_1 | Mg^2+^ | Mn^2+^ | 100 | 100 | 54 | 92 | 84 | 11 | 0 | 100 | 0 | 100 | 100 | 100 | 100 | 100 | 100 | 100 | 100 |
|  | K^+^ | Mn^2+^ | 98 | 96 | 70 | 75 | 70 | 47 | 99 | 100 | 100 | 100 | 1 | 100 | 100 | 100 | 100 | 100 | 100 |
|  | Mg^2+^ | K^+^ | 99 | 86 | 5 | 1 | 5 | 2 | 0 | 100 | 0 | 100 | 100 | 25 | 24 | 21 | 6 | 5 | 24 |
|  | K^+^ | K^+^ | 97 | 99 | 76 | 69 | 78 | 86 | 79 | 85 | 79 | 92 | 11 | 20 | 85 | 74 | 82 | 19 | 38 |
| *X.ory*_cf1_2 | Mg^2+^ | Mn^2+^ | 100 | 99 | 57 | 90 | 86 | 3 | 0 | 100 | 0 | 100 | 100 | 100 | 100 | 100 | 100 | 100 | 100 |
|  | K^+^ | Mn^2+^ | 93 | 97 | 82 | 45 | 86 | 11 | 99 | 100 | 99 | 100 | 1 | 100 | 100 | 100 | 100 | 100 | 100 |
|  | Mg^2+^ | K^+^ | 100 | 99 | 84 | 2 | 15 | 24 | 0 | 100 | 0 | 100 | 100 | 99 | 99 | 100 | 25 | 4 | 100 |
|  | K^+^ | K^+^ | 87 | 95 | 54 | 37 | 47 | 60 | 35 | 94 | 62 | 97 | 5 | 72 | 34 | 83 | 76 | 59 | 23 |

**Supplementary Table 2 Continuation:** 6CB3_chA_U44canonic and 6CB3_chA_U44_N3-deprot signify simulations starting from the 6CB3 chain A structure with canonical and N3-deprotonated U44, respectively (see **Supplementary Table 1**).

|  | **Ions** | | | **Stacking interactions**  **[%]** | | | | | | **Occupancy of M_A_ [%]** | | | | | **Occupancy of M_B_**  **[%]** | | | | | | | **M_C_**  **[%]** | | |
| --- | --- | --- | --- | --- | --- | --- | --- | --- | --- | --- | --- | --- | --- | --- | --- | --- | --- | --- | --- | --- | --- | --- | --- | --- |
|  | **M_A_** | **M_B_** | **M_C_** | **A9 U39** | **U39 C40** | **C40 A41** | **A41 C45** | **U44 C45** | **A42 U43** | **G7 OP1** | **G8 OP2** | **A9 OP1** | **U39 OP1** | **U44 O2** | **G8 OP1** | **U39 OP2** | **C40 OP2** | **A41 N7** | **U44 O2** | **N3** | **O4** | **A41 O2'** | **U43 OP1** | **U44 O4** |
| 6CB3 chA **U44 canonic** | Mg^2+^ | Mn^2+^ | Mg^2+^ | 100 | 98 | 66 | 99 | 55 | 86 | 100 | 100 | 100 | 100 | 0 | 100 | 100 | 100 | 100 | 0 | 0 | 0 | 0 | 100 | 0 |
|  | K^+^ | Mn^2+^ | Mg^2+^ | 100 | 94 | 41 | 0 | 0 | 94 | 100 | 100 | 100 | 100 | 29 | 100 | 100 | 100 | 100 | 0 | 0 | 100 | 0 | 100 | 0 |
|  | Mg^2+^ | K^+^ | Mg^2+^ | 100 | 96 | 81 | 5 | 97 | 83 | 100 | 100 | 100 | 100 | 100 | 99 | 96 | 97 | 5 | 0 | 16 | 99 | 0 | 100 | 0 |
|  | K^+^ | K^+^ | Mg^2+^ | 99 | 96 | 73 | 97 | 55 | 98 | 94 | 85 | 85 | 83 | 89 | 48 | 97 | 96 | 98 | 89 | 2 | 0 | 0 | 100 | 100 |
|  | Mg^2+^ | Mn^2+^ | K^+^ | 100 | 99 | 59 | 100 | 39 | 93 | 100 | 100 | 100 | 100 | 0 | 100 | 100 | 100 | 100 | 3 | 1 | 0 | 0 | 10 | 4 |
|  | K^+^ | Mn^2+^ | K^+^ | 100 | 96 | 44 | 1 | 1 | 95 | 100 | 100 | 100 | 100 | 25 | 100 | 100 | 100 | 100 | 0 | 0 | 100 | 0 | 13 | 0 |
|  | Mg^2+^ | K^+^ | K^+^ | 98 | 86 | 56 | 24 | 25 | 78 | 100 | 100 | 100 | 100 | 100 | 90 | 44 | 85 | 38 | 0 | 15 | 83 | 0 | 47 | 38 |
|  | K^+^ | K^+^ | K^+^ | 89 | 93 | 93 | 7 | 78 | 11 | 95 | 97 | 91 | 100 | 82 | 46 | 71 | 80 | 5 | 82 | 19 | 40 | 6 | 19 | 10 |
| 6CB3 chA **U44 N3-deprot** | Mg^2+^ | Mn^2+^ | Mg^2+^ | 100 | 98 | 75 | 71 | 6 | 55 | 100 | 100 | 100 | 100 | 0 | 100 | 100 | 100 | 100 | 100 | 6 | 0 | 1 | 100 | 100 |
|  | K^+^ | Mn^2+^ | Mg^2+^ | 100 | 85 | 74 | 75 | 1 | 61 | 100 | 100 | 100 | 98 | 0 | 100 | 100 | 100 | 100 | 100 | 85 | 0 | 2 | 100 | 100 |
|  | Mg^2+^ | K^+^ | Mg^2+^ | 98 | 72 | 49 | 23 | 3 | 93 | 100 | 100 | 100 | 100 | 100 | 84 | 99 | 57 | 43 | 24 | 62 | 18 | 0 | 100 | 100 |
|  | K^+^ | K^+^ | Mg^2+^ | 99 | 94 | 53 | 98 | 46 | 94 | 91 | 81 | 91 | 83 | 0 | 38 | 100 | 96 | 99 | 92 | 32 | 0 | 0 | 100 | 100 |
|  | Mg^2+^ | Mn^2+^ | K^+^ | 100 | 99 | 44 | 98 | 3 | 95 | 100 | 100 | 100 | 100 | 100 | 100 | 100 | 100 | 100 | 0 | 100 | 100 | 9 | 43 | 24 |
|  | K^+^ | Mn^2+^ | K^+^ | 100 | 98 | 71 | 40 | 4 | 34 | 100 | 98 | 92 | 100 | 14 | 100 | 100 | 100 | 83 | 94 | 68 | 6 | 16 | 32 | 19 |
|  | Mg^2+^ | K^+^ | K^+^ | 99 | 23 | 75 | 92 | 13 | 98 | 100 | 100 | 100 | 100 | 100 | 87 | 100 | 0 | 92 | 89 | 99 | 11 | 0 | 43 | 33 |
|  | K^+^ | K^+^ | K^+^ | 100 | 96 | 84 | 85 | 7 | 17 | 99 | 100 | 98 | 100 | 98 | 1 | 100 | 99 | 100 | 13 | 8 | 0 | 0 | 16 | 6 |

**Supplementary Figure 15**


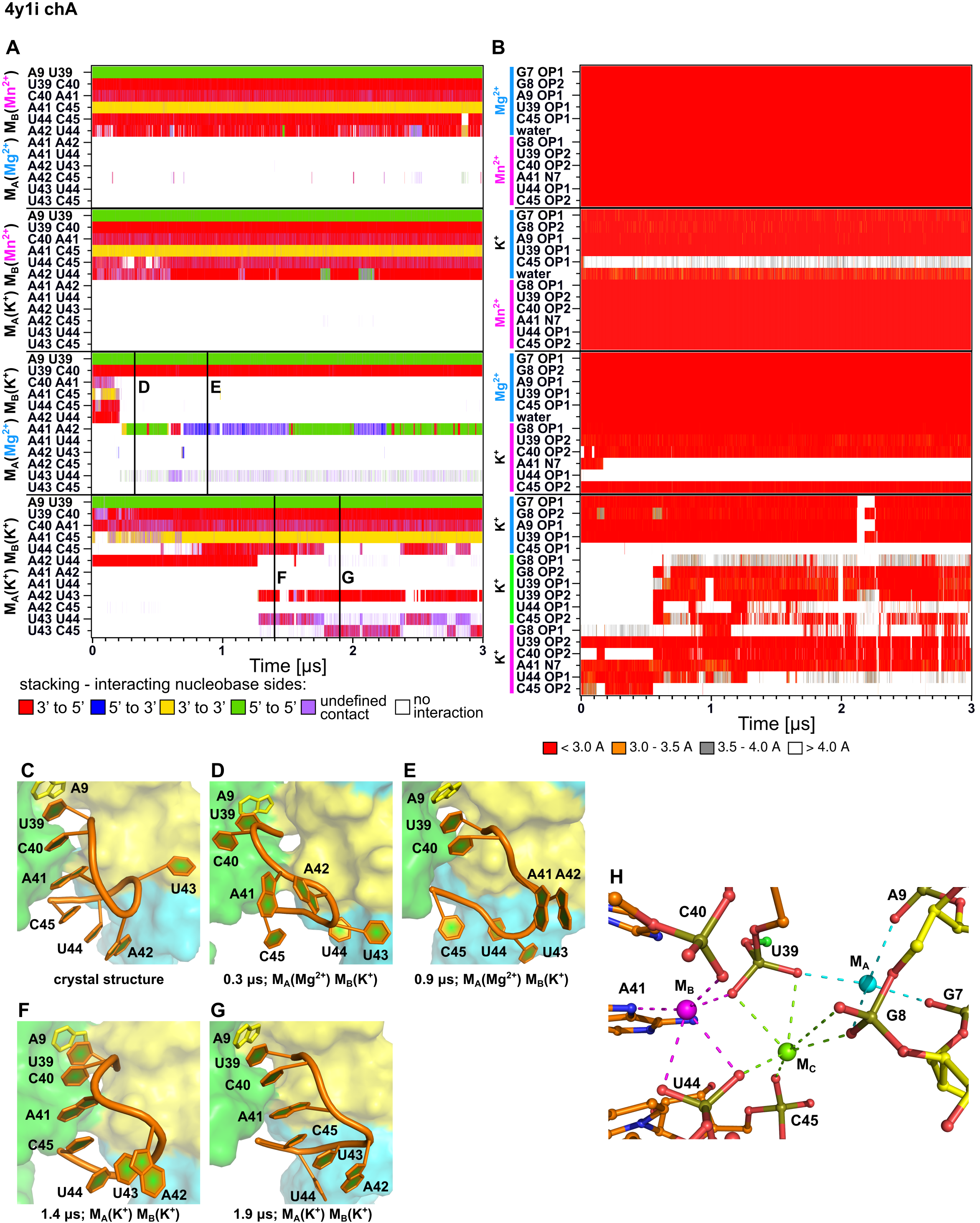


**Supplementary Figure 15|** Conformational behavior of the L3 loop and the M_A_ and M_B_ ion binding sites in MD simulations of the *L. lactis* riboswitch (4Y1I chain A). (**A**) Time evolution of the stacking pattern of the loop; the colors correspond to different mutual orientations of nucleobases in stacking interactions indicated by the corresponding faces (3'-face and 5'-face) involved in the interaction. (**B**) Time evolution of ligand-ion interactions in the ion binding sites. (**C-G**) Close view of the stacking patterns; panel C corresponds to the crystal structure while the other panels depict structures observed in MD simulations at the times indicated by vertical bars in the panel A. (**H**) New ion binding sites appear in simulations where both divalents were replaced by K^+^ (the colors of the ions match the colors of the bars in panel B, helping to identify the particular binding sites).

**Supplementary Figure 16**


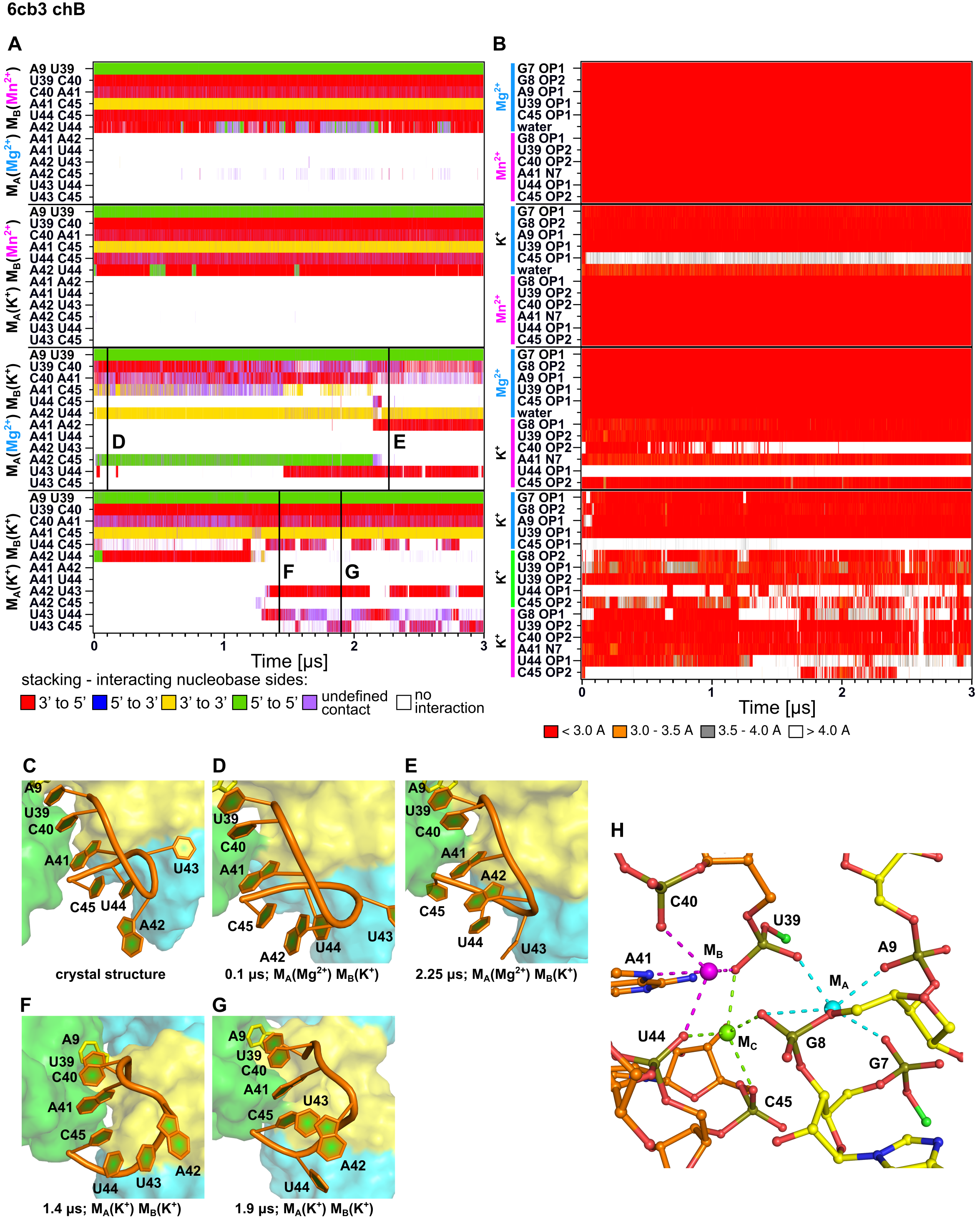


**Supplementary Figure 16|** Conformational behavior of the L3 loop and the M_A_ and M_B_ ion binding sites in MD simulations of the *L. lactis* ribozyme (6CB3 chain B). (**A**) Time evolution of the stacking pattern of the loop; the colors correspond to different mutual orientations of nucleobases in stacking interactions indicated by the corresponding faces (3'-face and 5'-face) exposed to the interaction. (**B**) Time evolution of ligand-ion interactions in the ion binding sites. (**C-G**) Close view on the stacking pattern; panel C corresponds to the crystal structure while the others depict structures observed in MD simulations at the times indicated by vertical bars in panel A. (**H**) New ion binding sites appear in simulations where both divalents were replaced by K^+^ (the colors of the ions match the colors of the bars in panel B, helping to indicate the particular binding site).

**Supplementary Figure 17**

**
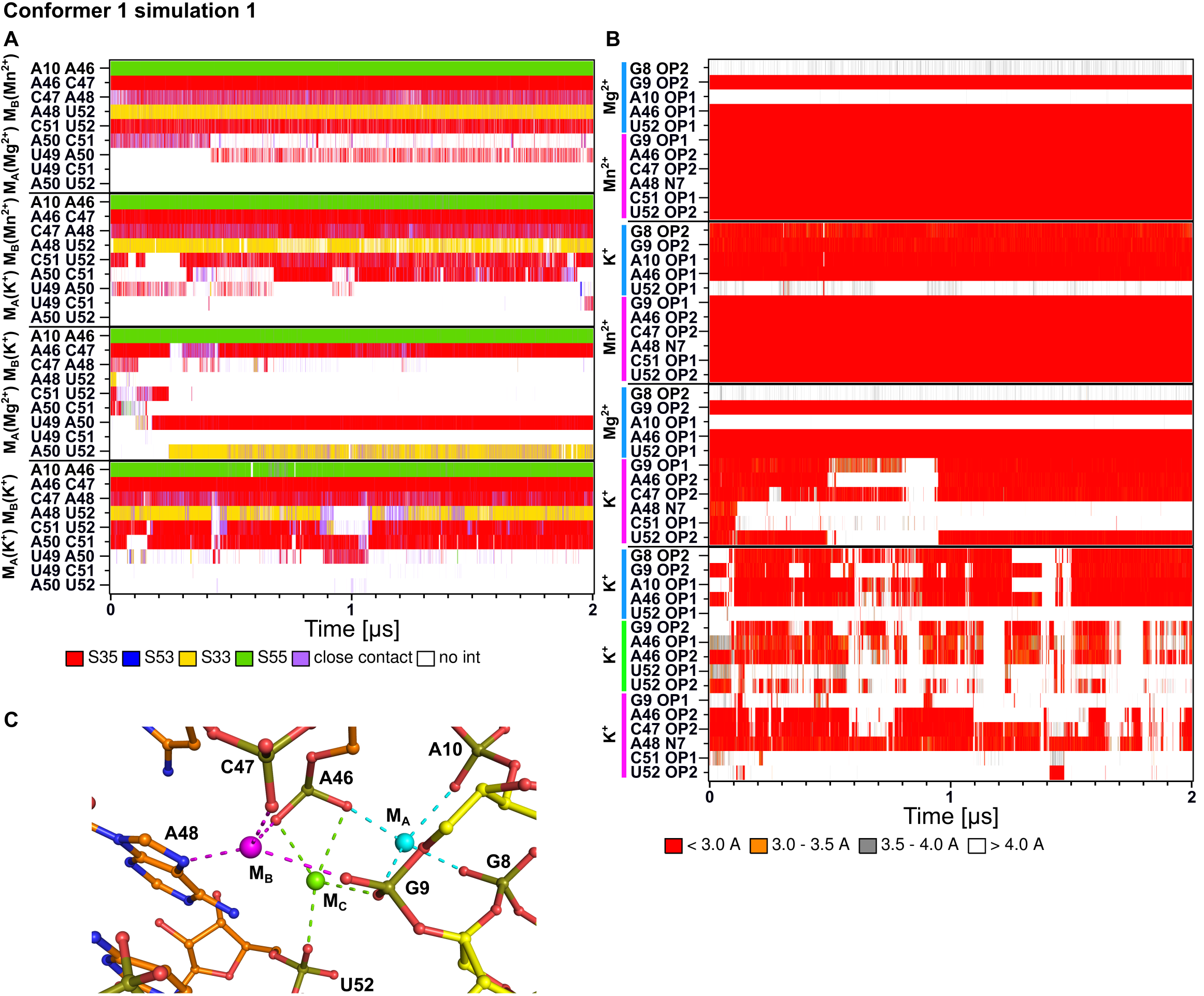
**

**Supplementary Figure 17**| Conformational behavior of the L3 loop and M_A_ and M_B_ ion binding sites in MD simulations of the *X. oryzae* riboswitch. (**A**) Time evolution of the stacking pattern of the loop; the colors correspond to different mutual orientations of nucleobases in stacking interactions indicated by the corresponding faces (3'-face and 5'-face) involved in the interaction. (**B**) Time evolution of ligand-ion interactions in the ion binding sites. (**C**) New ion binding sites appear in simulations where both divalents were replaced by K^+^ (the colors of the ions match the colors of the bars in panel B, helping to indicate the particular binding site). Here only the data from the *X. oryzae* Conformer 1 simulation 1 are shown, while the data from other simulations are presented below.

**Supplementary Figure 18**

**
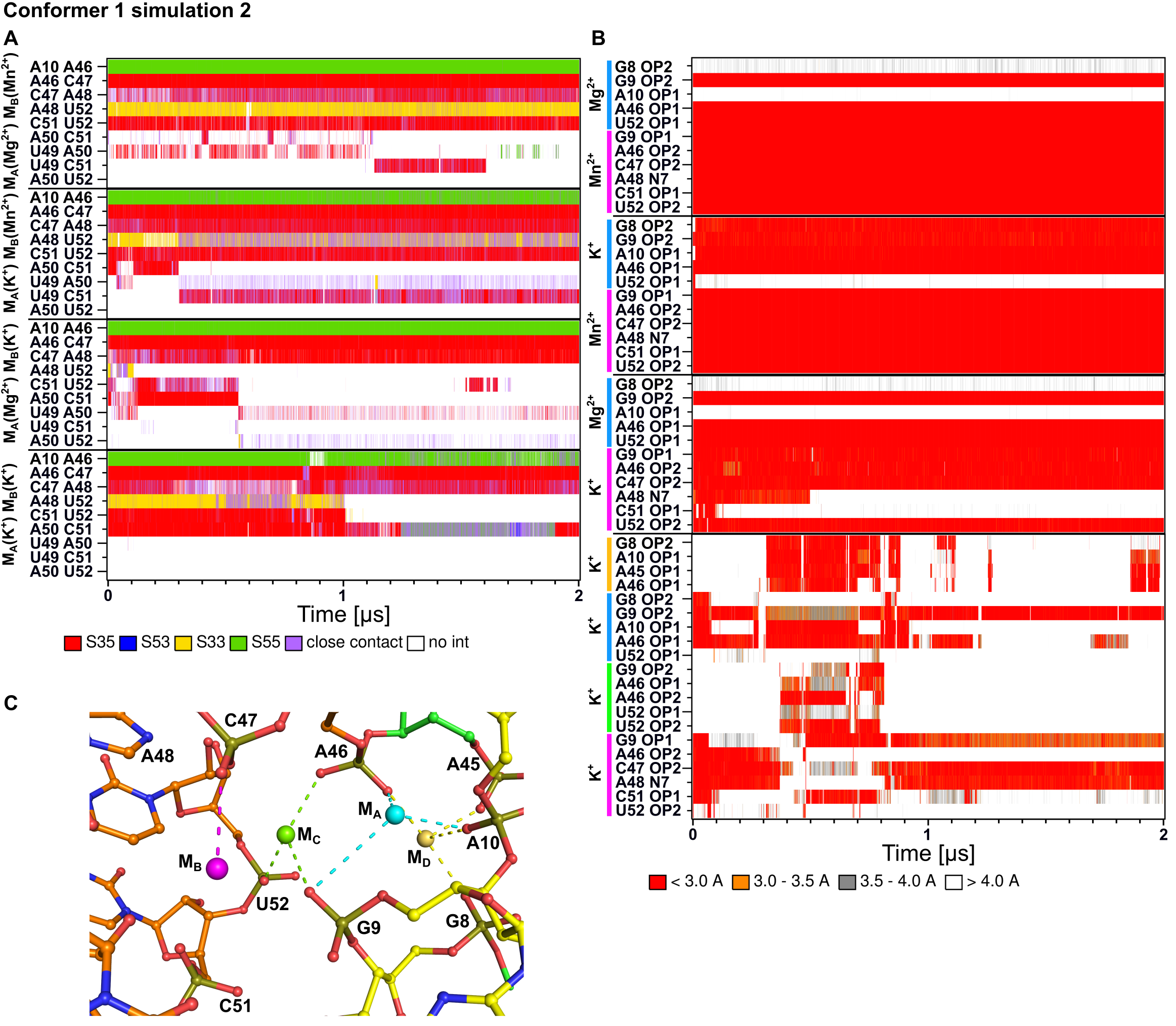
**

**Supplementary Figure 18|** Continuation of panels A-C for the data from *X. oryzae* Conformer 1 simulation 2.

**Supplementary Figure 19**

**
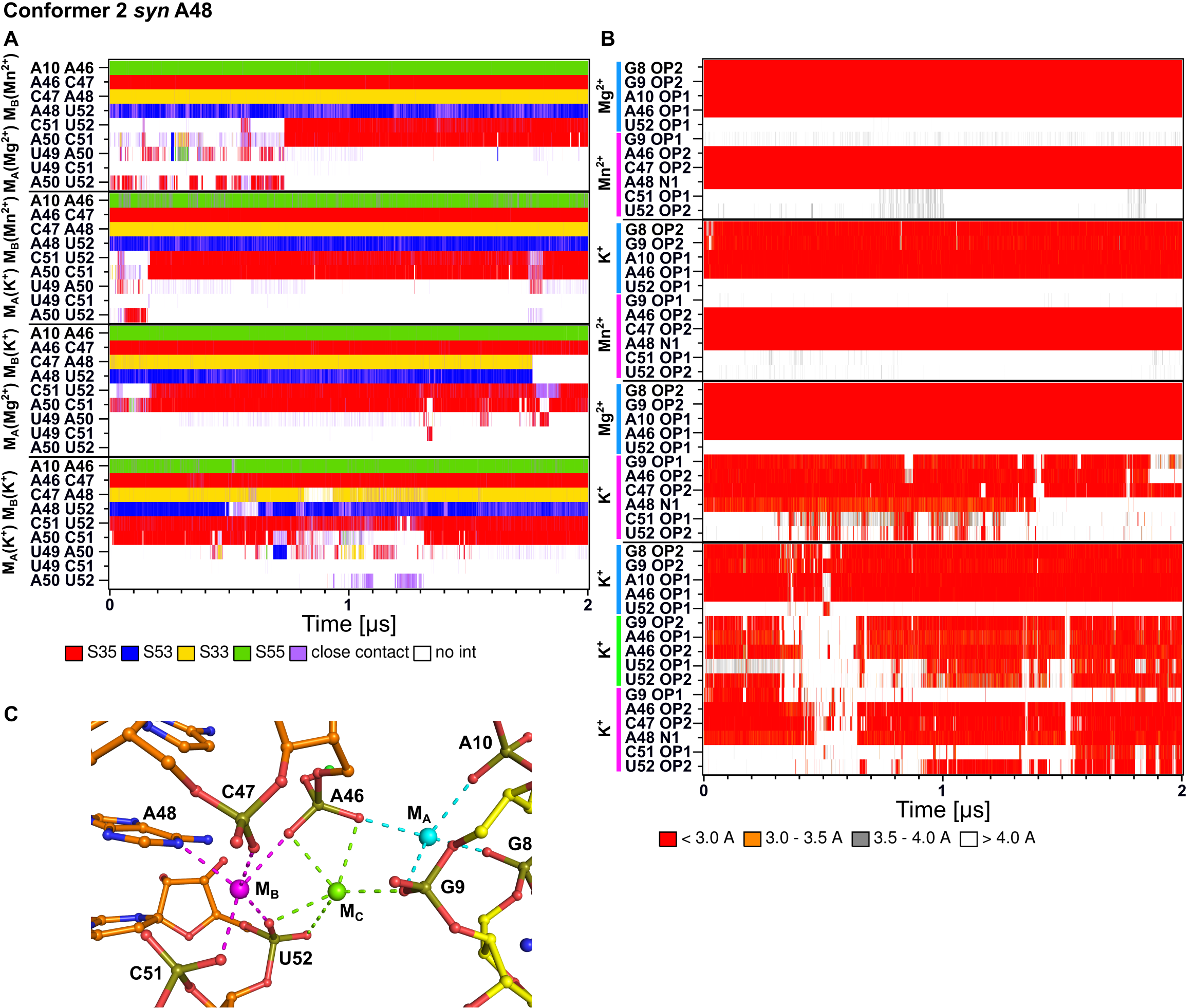
**

**Supplementary Figure 19|** Continuation of panels A-C for the data from *X. oryzae* Conformer 2 (the simulation with *syn*-oriented A48, see **Supplementary Table 1**)

**Supplementary Figure 20**

**
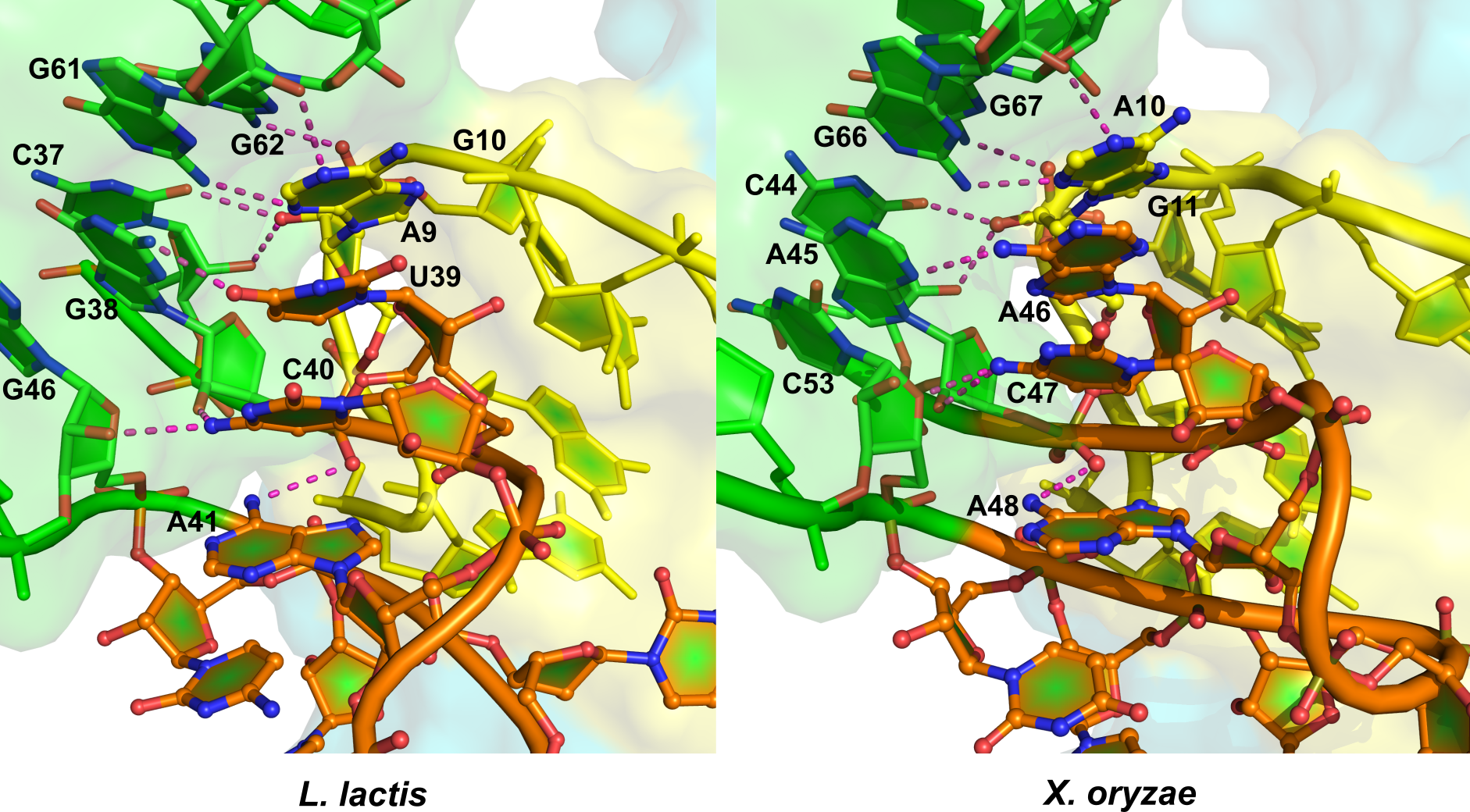
**

**Supplementary Figure 20**| The stacking pattern formed by A10 (A9 according to numbering of *L. lactis*) and nucleobases of loop L3 with depicted tertiary interactions (see **Supplementary Figure 21** for their structural stabilities in MD simulations).

**Supplementary Figure 21**

**
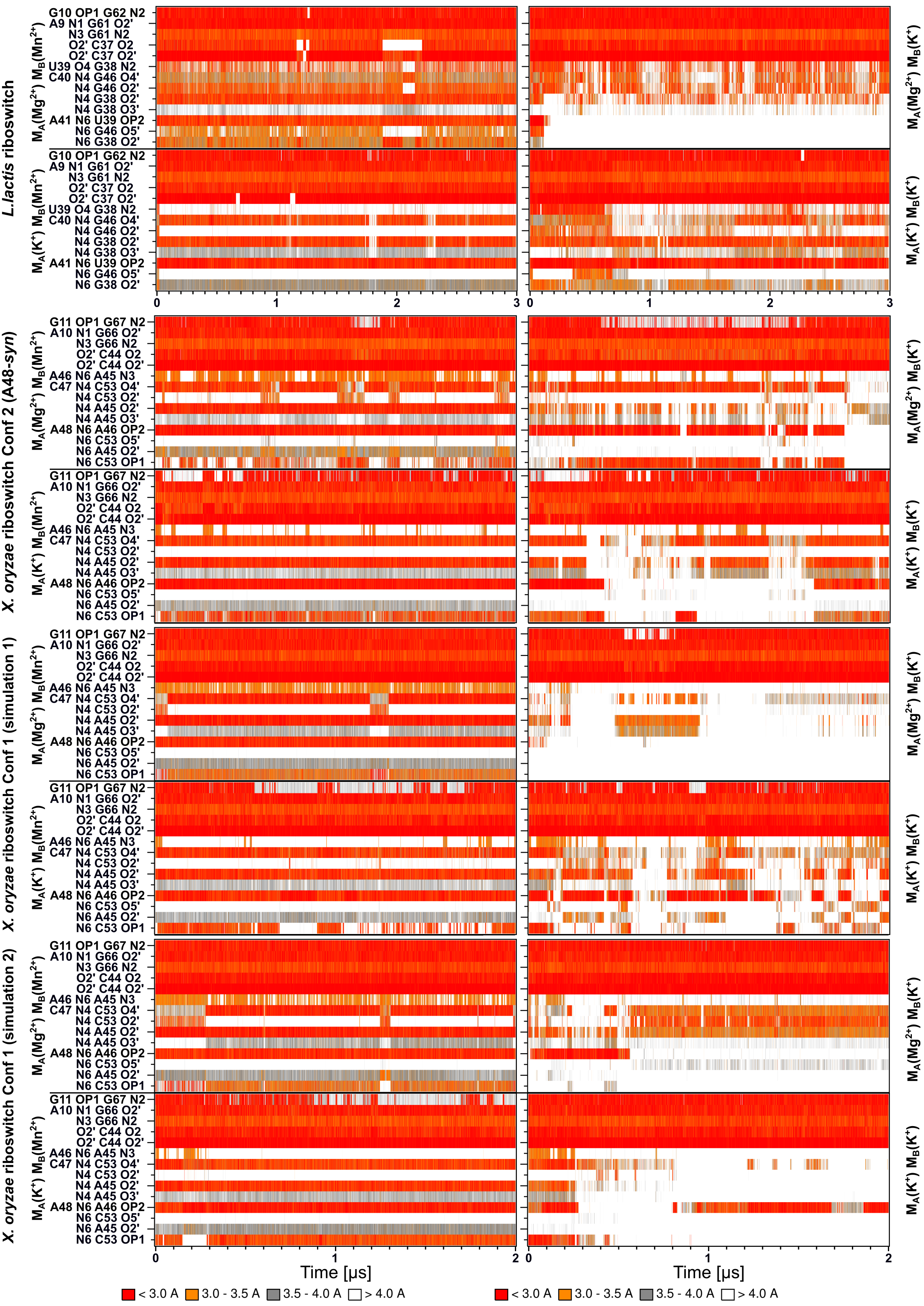
**

**Supplementary Figure 21|** Time evolution of tertiary interactions of nucleotides included in the stacking pattern of L3 loop (see **Supplementary Figure 20** for structural view of these tertiary contacts).

**Supplementary Figure 22**


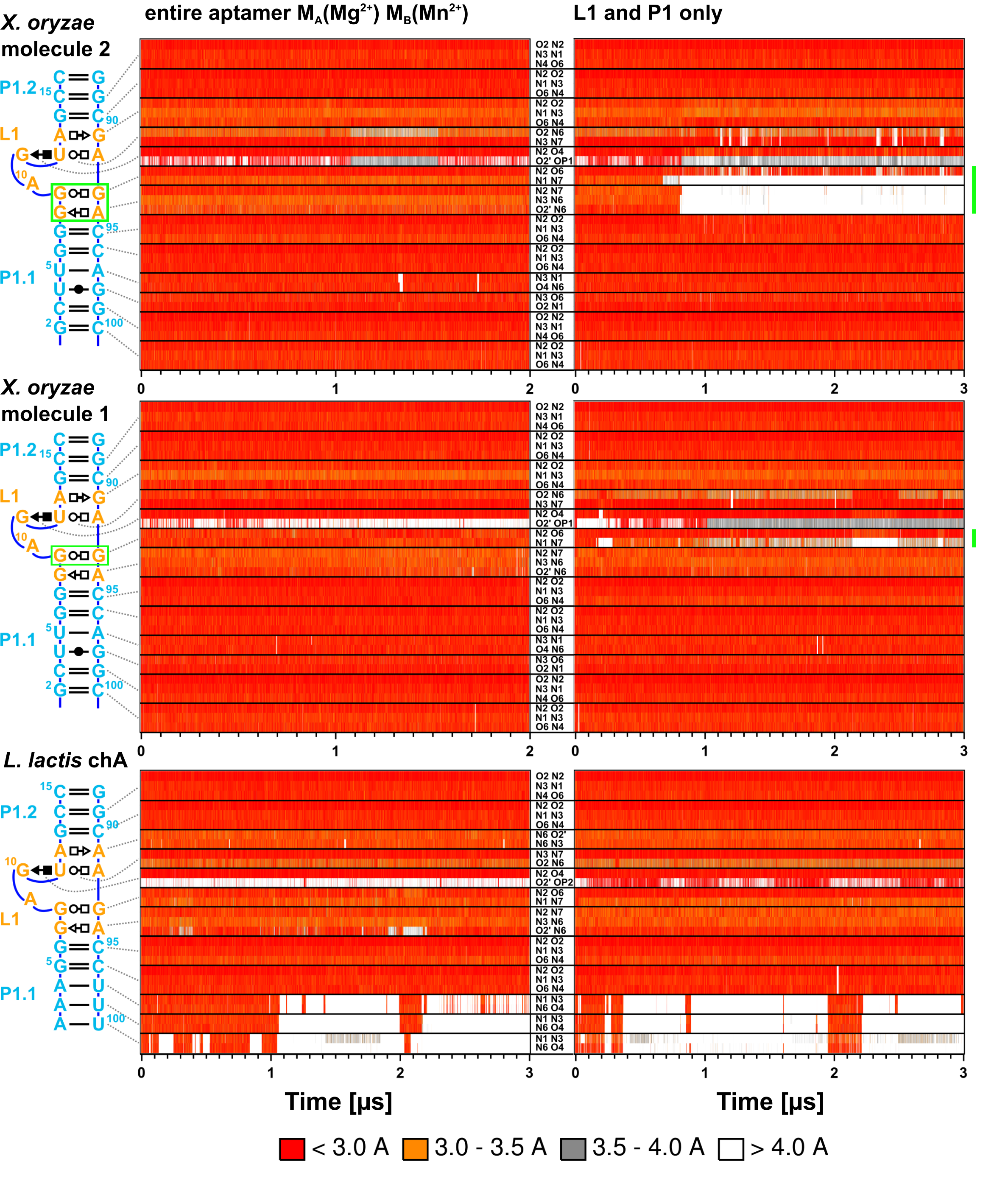


**Supplementary Figure 22**. Time evolution of native base pairing interactions of SRL-like motif of L1 in entire aptamer and in model structure (consisting only of P1.1, P1.2 and L1 and thus lacking A-minor interaction of A10 in *X. oryzae* and A9 in *L. lactis*, to L3 loop and P3 stem). The most labile interactions are highlighted in green.

**Supplementary Figure 23**


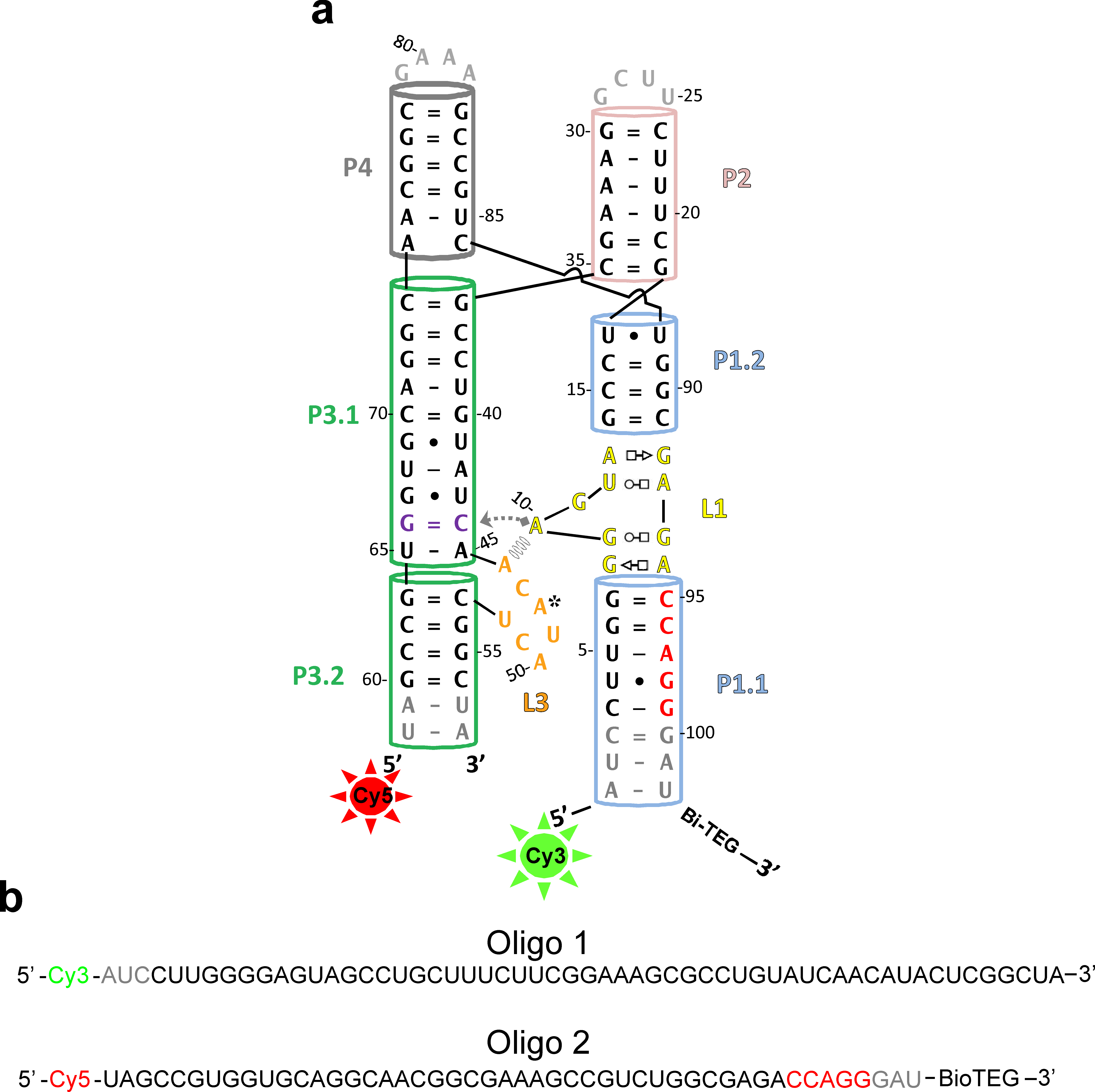


**Supplementary Figure 23|** smFRET construct design. (**a**) Sequence and secondary structure of the *Xory* *yybP-ykoY* riboswitch construct used for smFRET. The numbering of residues is kept consistent with the crystal construct of the RNA in Fig. 1a. The construct was made was hybridizing two chemically synthesized RNA oligonucleotides modifications at their 5' and 3' ends with Cy3, Cy5 fluorophores and Biotin-TEG. (**b**) Sequences of the two oligonucleotides with modifications used for the smFRET construct shown in (a)

**Supplementary Figure 24**


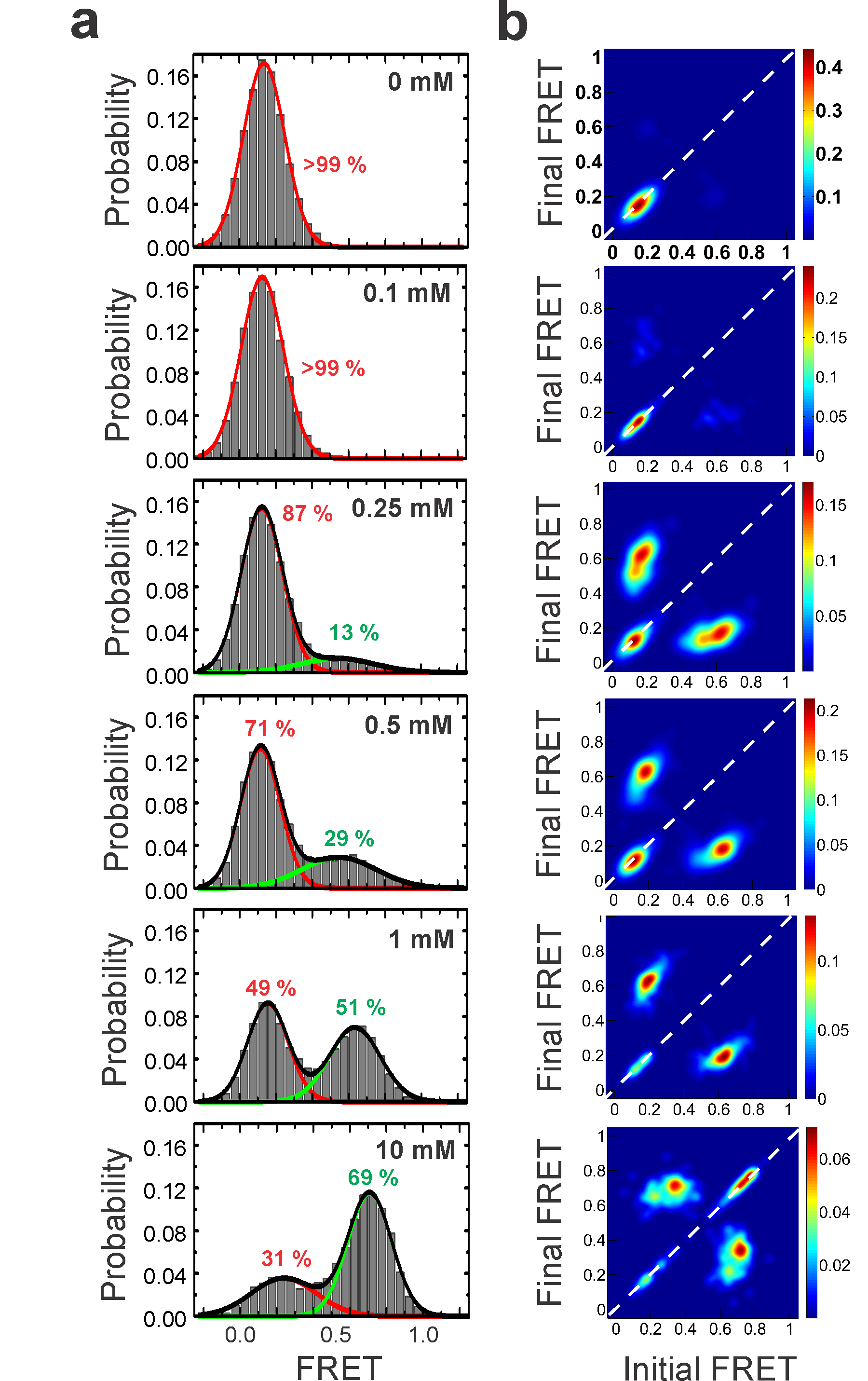


**Supplementary Figure 24| Mg^2+^ titration of the WT *Xory* riboswitch.** **(a)** FRET histograms showing the distribution of the two FRET states under various Mg^2+^ concentrations, fit to a sum of Gaussian functions. The Gaussian peaks for the low- and high-FRET states are shown in red and green, respectively, while the cumulative fit is shown in black. The histograms under 0 mM and 0.1 mM contain very low populations of the high-FRET state and were fit to a single Gaussian function. (**b**) TODPs under different Mg^2+^ concentrations showing the fraction of static ‘on-diagonal’ and dynamic ‘off-diagonal’ molecules. The SD populations under the high, 10 mM Mg^2+^ is evident in the TODP.

**Supplementary Figure 25**


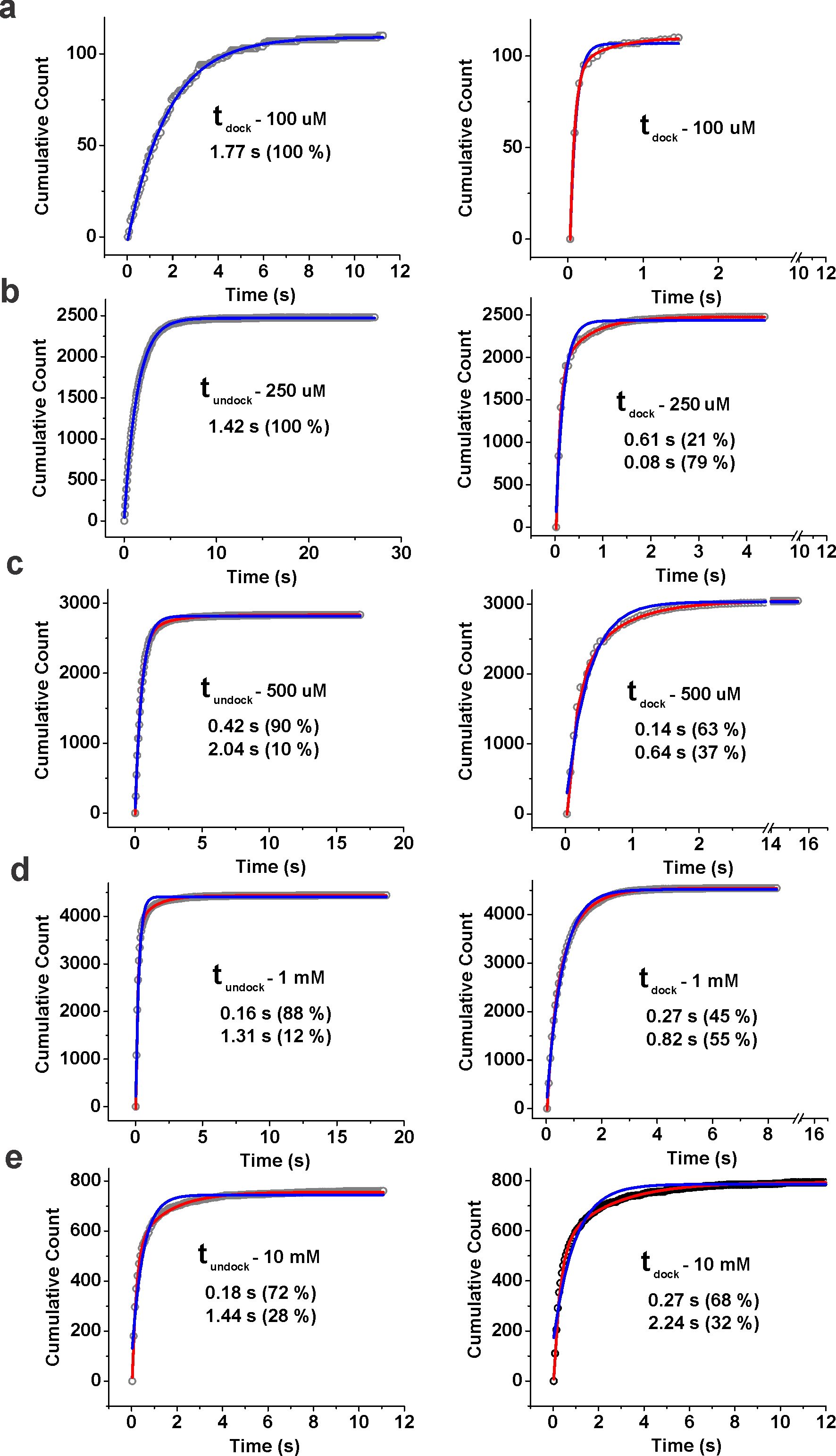


**Supplementary Figure S25| Kinetics of WT *Xory* Mn^2+^ sensing riboswitch. (a)** Cumulative dwell-time distributions of t_undock_ and t_dock_ in the presence of 0.1 mM MgCl_2_ fit to single (blue) and double-exponential (red) are shown. The life-times and amplitudes of slow and fast components are also shown. In case of double-exponential fits, fit to single exponential function is also shown for comparison. **(b-e)** Same as in (a) but in the presence of 0.25 mM, 0.5 mM, 1 mM and 10 mM MgCl_2_, respectively.

**Supplementary Figure 26**

**
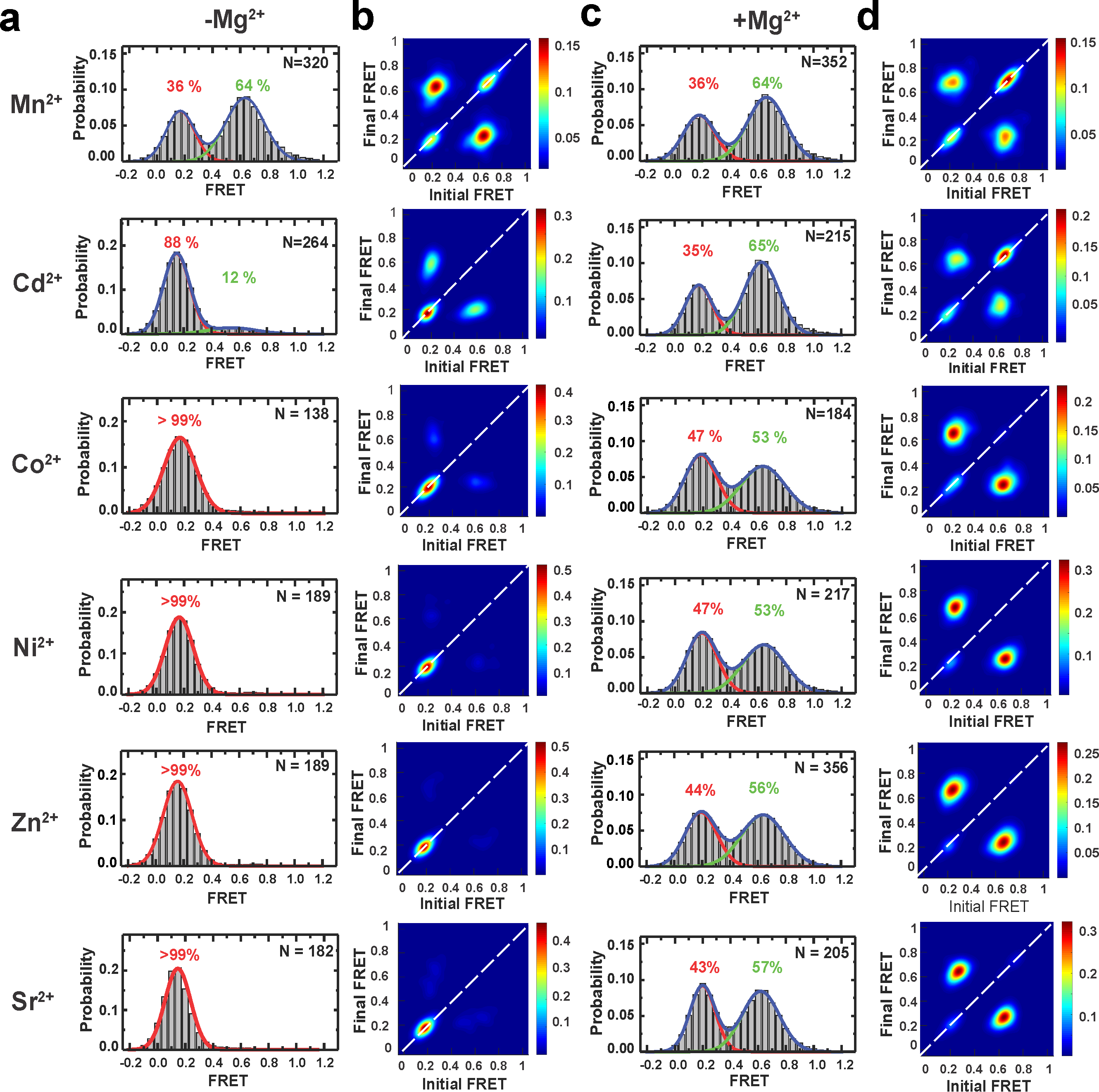
**

**Supplementary Figure S26| Effect of different metal ions on the WT *Xory* riboswitch. (a)** FRET histograms showing equilibrium distribution of docked and undocked conformations of the WT riboswitch at 0.1 mM concentration of different transition metal ions alone (i.e., in the absence of 1 mM MgCl_2_). Gaussian fits to the docked, undocked are shown as green and red curves, while the cumulative fit is shown in blue. The number of molecules, N, for each condition analyzed and the % of docked and undocked conformations in each condition are indicated. **(b)** TODPs corresponding to the histograms in (a). **(c)** FRET histograms and **(d)** TODPs at 0.1 mM concentration of different metal ions in the presence of of 1 mM MgCl_2_. For the different metal ions tested, except Mn^2+^ and Cd^2+^, almost all the traces remained in SU conformation, with >95 % in the low-FRET (~0.1) undocked conformation, in the absence of Mg^2+^.

**Supplementary Figure 27**


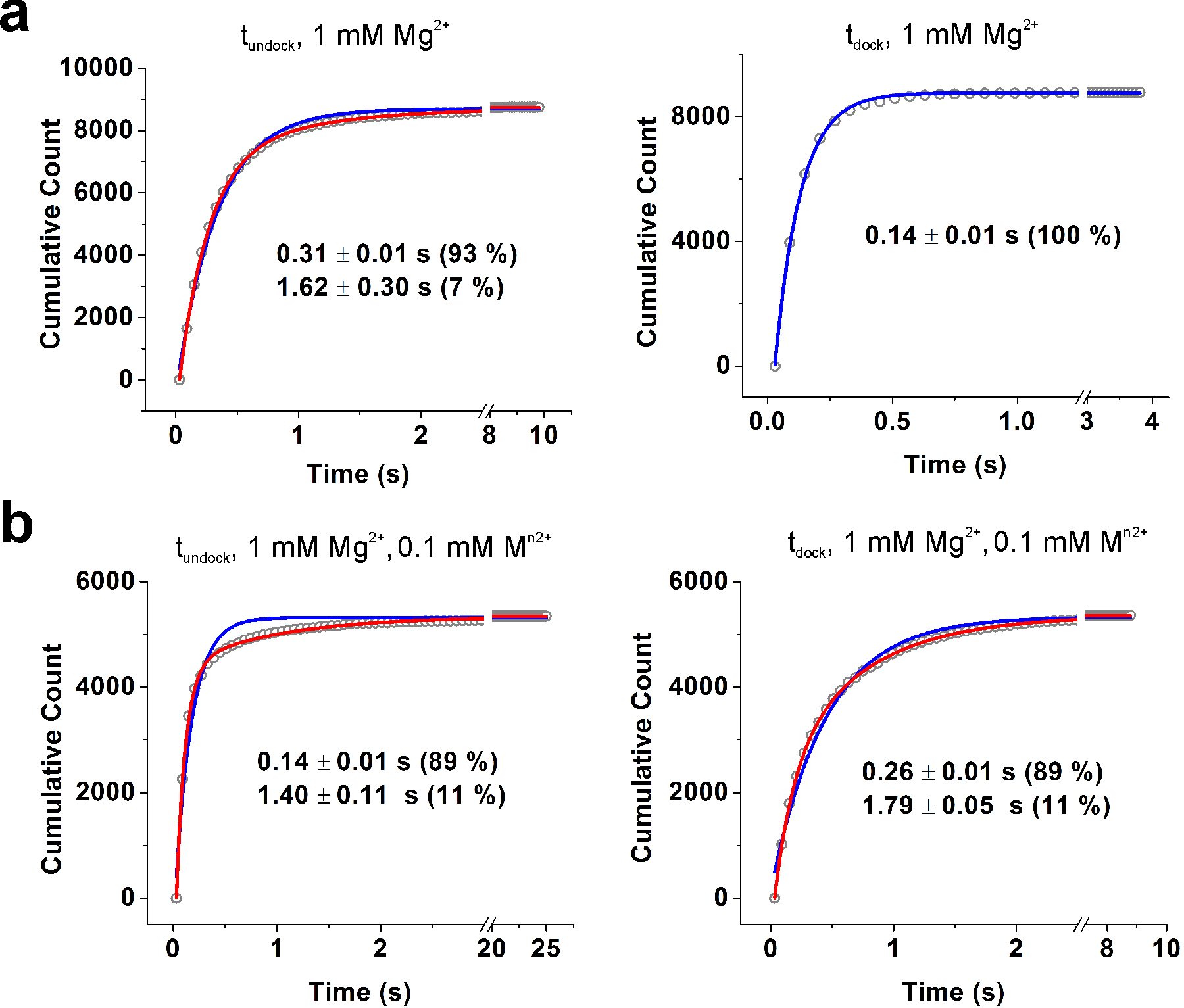


**Supplementary Figure 27| Kinetics of the *Xory* riboswitch A48U mutant. (a)** Cumulative dwell-time distributions of t_undock_ and t_dock_ in the presence of 1 mM MgCl_2_ fit with single (blue) and double-exponential (red) functions. The life-times and amplitudes of slow and fast components are also shown. In the case of double-exponential fits, fit to single exponential is also shown for comparison. **(b)** Same as in (a) but in the presence of 1 mM MgCl_2_ and 0.1 mM MnCl_2_.

**Supplementary Figure 28**


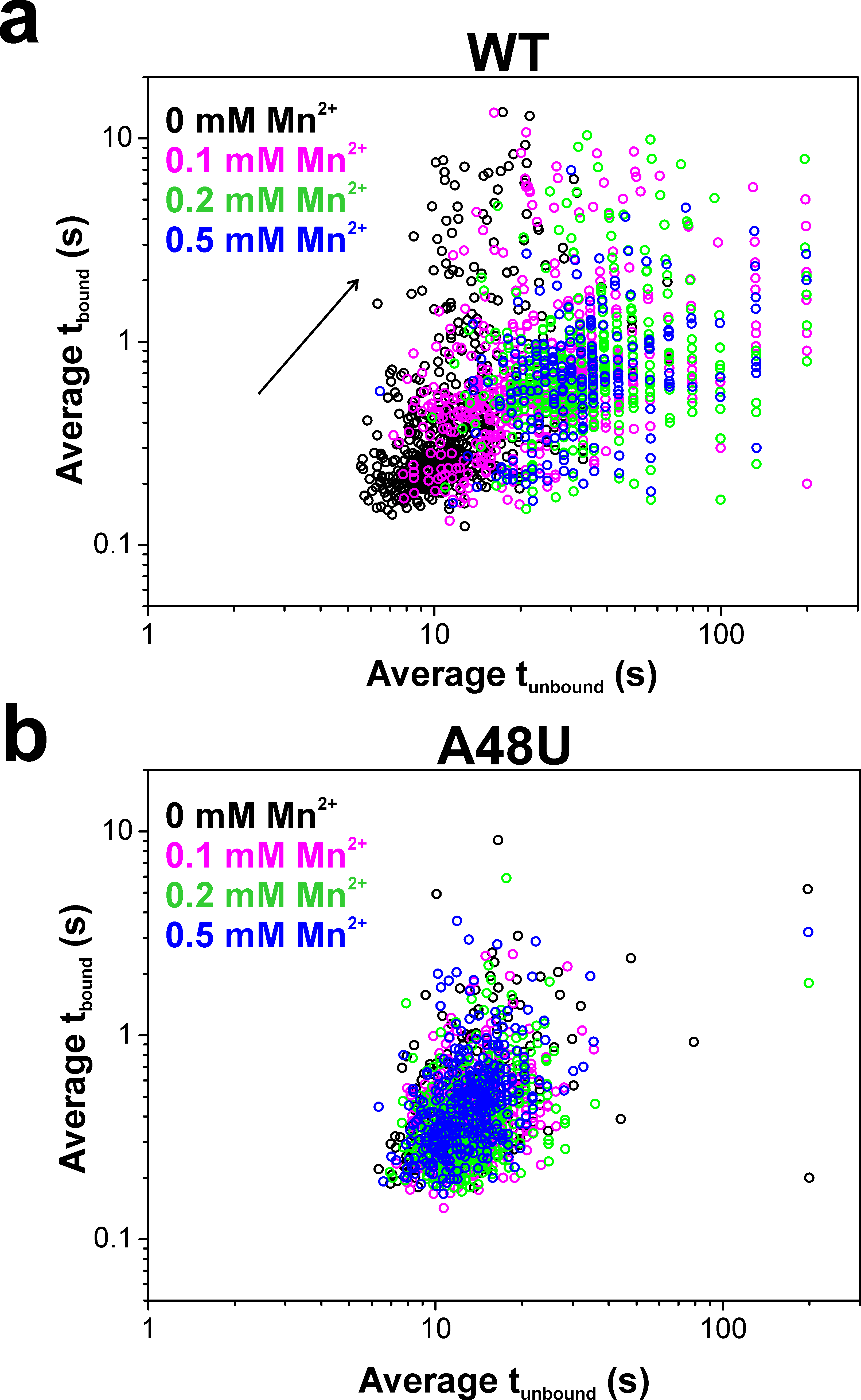


**Supplementary Figure 28| Scatter plots of average bound and unbound times for the DNA oligo. (a)** Scatter plot of average t_unbound_ *vs* average t_bound_ of the DNA oligo to the WT riboswitch in the presence of 1 mM Mg^2+^ and different Mn^2+^ concentrations. For every condition, over 200 single-molecule binding traces, each containing multiple binding events are analyzed and the average t_unbound_ and t_bound_ for individual traces are shown. The distribution shifts diagonally upwards and to the right indicating increasing average t_unbound_ and t_bound_, under increasing [Mn^2+^] showing decreasing *k_on_* (1/t_unbound_) and *k_off_* (1/t_bound_). (**b**) Same as in (a) but for the A48U mutant riboswitch. The distribution remains similar overall under increasing [Mn^2+^], showing little change in *k_on_* and *k_off_.*

**Supplementary Figure 29**


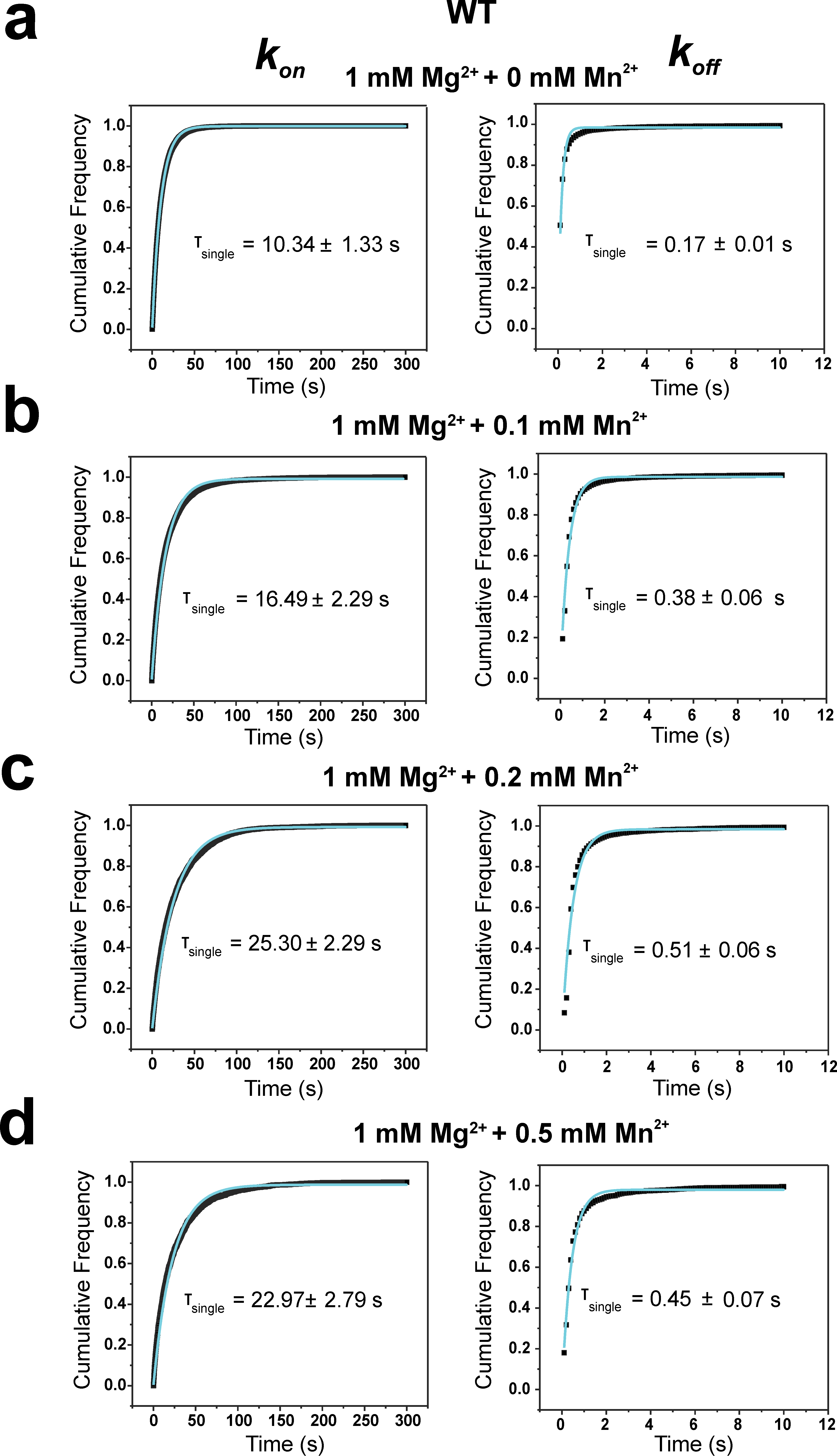


**Supplementary Figure 29| Kinetics fits of SiM-KARTS data for the WT Mn^2+^ riboswitch.** Cumulative dwell-time distribution plots of t_unbound_ and t_bound_, fit to single-exponential functions to obtain *k_on_* and *k_off_* in the presence of 1 mM Mg^2+^ and (**a**) 0 mM Mn^2+^, (**b**) 0.1 mM Mn^2+^, (**c**) 0.2 mM Mn^2+^, and (**d**) 0.5 mM Mn^2+^. The single-exponential fits to the data are shown in cyan along with the individual unbound and bound life-times.

**Supplementary Figure 30**


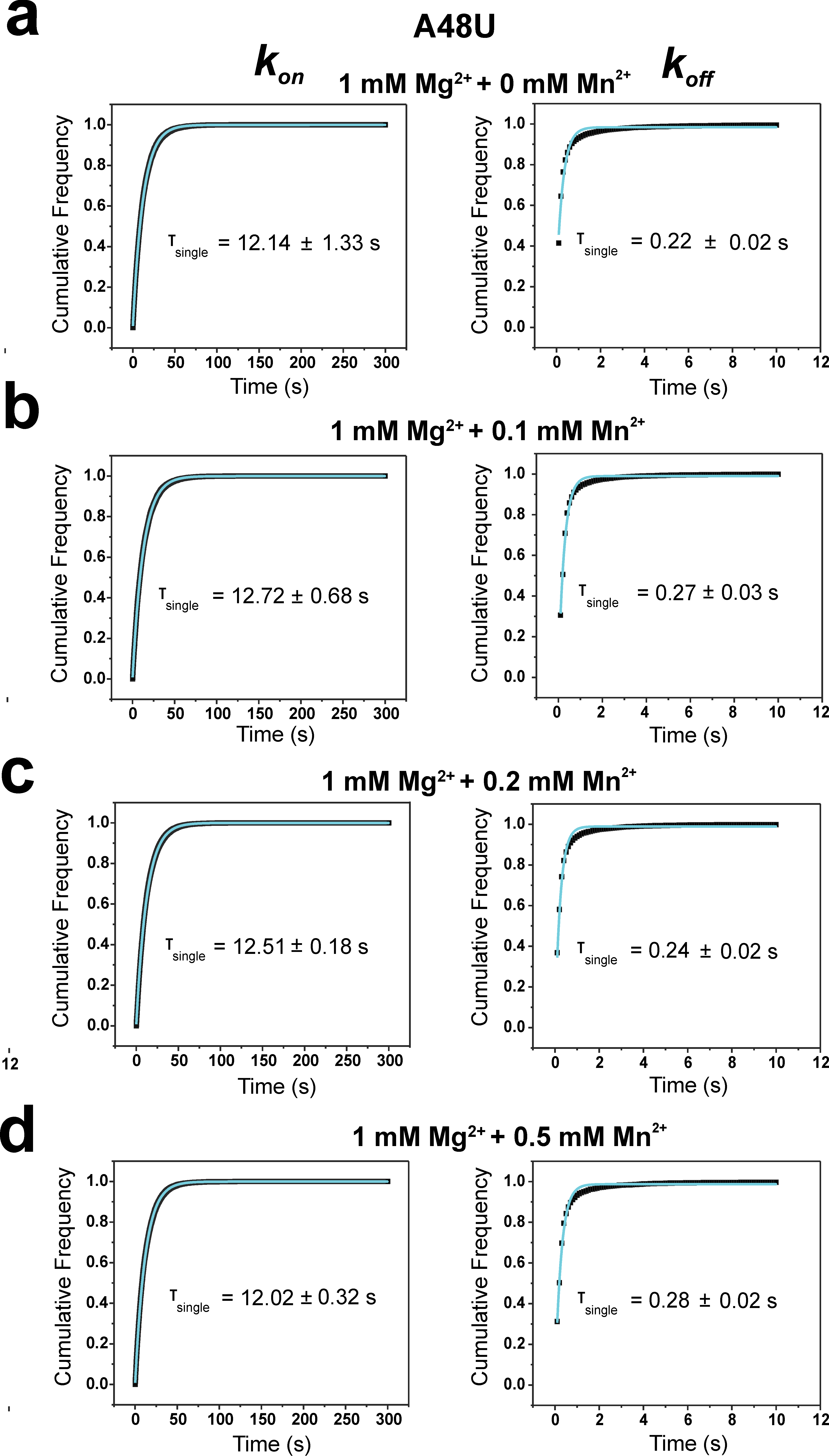


**Supplementary Figure 30| Kinetics fits of SiM-KARTS data for the A48U mutant Mn^2+^ riboswitch.** Cumulative dwell-time distribution plots of t_unbound_ and t_bound_, fit to single-exponential functions to obtain *k_on_* and *k_off_* in the presence of 1 mM Mg^2+^ and (**a**) 0 mM Mn^2+^, (**b**) 0.1 mM Mn^2+^, (**c**) 0.2 mM Mn^2+^, and (**d**) 0.5 mM Mn^2+^. The single-exponential fits to the data are shown in cyan along with the individual unbound and bound life-times.

**Supplementary Note 1 Global structural dynamics of docked structures of aptamers in the MD simulations**

To elucidate structural dynamics of the Mn^2+^ sensing riboswitch, we performed a set of explicit solvent MD simulations on microseconds time scale. In addition to simulations containing both M_A_ and M_B_ ion binding sites occupied by divalent ions, we performed a set of simulations testing the effect of replacement of these divalent ions by monovalents in each ion binding position separately or in both of them simultaneously (see **Supplementary** **Table 2**).

The global structural dynamics of the studied systems was visualized by the B-factors calculated per residues (**Supplementary** **Figure 7**). All simulations revealed similar trends in global structural dynamics except of behavior of L3 loop, which was found to be sensitive to type of ions in the M_A_ and M_B_ ion binding sites (see below). All stems exhibited high structural stability in all simulations. We observed larger fluctuations on the tips of P2, P3, and P4 stems. These fluctuations were found to be caused by unstacking of the L2 nucleotide, which should be considered as inherent part of native structural dynamics of GNRA tetraloops^2-6^. In the case of simulations of *L. lactis* structure, B-factor values indicated also large fluctuations of the P2 stem. Detailed analyses revealed that these fluctuations were connected with a reversible bending of the P2 stem and its movement toward and away from the P4 stem. This global movement was correlated with flipping of ε torsion angle of A28 nucleotide, which fluctuated between two states (see **Supplementary Figure 8**).

**Supplementary Note 2 MD simulations confirmed *syn* orientation of A48 in L3 loop of *X. oryzae* structure Conformer 2**

The electron density of the conformer 2 of the *X. oryzae* structure revealed adenine A48 in *syn-* rather than *anti-*conformation. In order to verify this unusual glycosidic bond orientation within the given structural context, we used two different refinements of the conformer 2 structure (**Supplementary Figure 9**) as starting structures for subsequent MD simulations. These simulations aimed to indirectly probe the orientation of A48 glycosidic bond via analyses of compatibility of a given orientation of A48 with its structural environment reflecting the native A48 orientation through molecular interactions. We thus expected that while simulation starting from native orientation of A48 will fluctuate around starting structure conformation, the non-native orientation of A48 in the starting structure should result in structural changes in initial phase of MD simulation. For sake of completeness, it is also possible that both orientations of A48 might coexist in the crystal lattice ensemble and electron density represents ensemble-averaged picture of A48, which is structurally compatible with its structural environment in both orientations. In such case, both A48 orientations would be equally tolerated by their structural environment and thus both would be equally stable in MD simulations.

Therefore, we performed MD simulations of conformer 2 both with *syn*- and *anti*-oriented A48. Namely, we compared simulations having their ion binding sites occupied by corresponding native divalent ions, i.e., by Mg^2+^ and Mn^2+^ in M_A_ and M_B_, respectively (see **Supplementary Table 2**). We observed that the simulation started from structure with *syn*-oriented A48 stably fluctuated around crystal conformation during the entire 2 μs simulation. In contrast, the simulation started with *anti*-oriented A48 revealed significant structural changes during initial phase of the simulation. Namely, the A48 nucleobase was shifted already in the initial geometrical optimization, so that it was coordinated to the Mn^2+^ ion by N7 nitrogen, while N6 exocyclic amino group was repelled away from the Mn^2+^ ion. This movement was accompanied by reconformation of the sugar-phosphate backbone between U52 and C53 that shifted away from A48 to avoid sterical clash (**Supplementary Figure 10**). Subsequently, in the early stages of the simulations, namely during thermal equilibration, such reconformation of U52-C53 sugar-phosphate backbone resulted in destabilization of the U52(H3)…A48(O2’) hydrogen bond and exposure of the U52 into solvent (**Supplementary Figure 10**). All these rapid structural changes should not be considered as native structural dynamics of the riboswitch and are unambiguously a consequence of starting structure bias, namely non-native refinement of A48 *anti* orientation. Thus, in agreement with the observed electron density, we found that *anti*-oriented A48 is not compatible with the overall structure of Conformer 2 containing open-conformation of loop L3. Thus, crystallographic data together with MD simulations provide clear evidence of *syn*-orientated A48 in this particular L3 loop conformation; the MD simulations of Conformer 2 started with *anti*-A48 were not further analyzed and discussed.

For the sake of completeness, it is worth noting that MD simulations of Conformer 1 (containing closed conformation of loop L3 and A48 clearly resolved as *anti*-orientated) did not reveal any rapid structural changes during the early stage relaxation that would point to any doubts of the refinement of the L3 loop conformation.

**Supplementary Note 3 MD simulations of chain A of the *L. lactis* 6CB3 structure support U44^-^ to be deprotonated in this particular arrangement involving three Cd^2+^ ions**

In chain A of the 6CB3 crystal structure, Bachas and Ferré-D’Amaré reported an unusual arrangement of the ion binding sites involving three Cd^2+^ ions. In this arrangement, the M_A,Mg_ and M_C_ ions were heptacoordinate, while the ion in the M_B,Mn_ site was reported to be hexacoordinate, in particular to two carbonyl groups of U44, the N7-nitrogen of A41, and three phosphate non-bridging oxygens of G40, U39, and G9. However, the U44(N3) nitrogen is located only 2.2 Å from the Cd^2+^ ion in the M_B,Mn_ site, which raises the question of its protonation state and its potential role as the seventh inner-shell ligand of thus heptacoordinated Cd^2+^ ion in the M_B,Mn_ site.

To address this question, we performed two sets of MD simulations, one set with a canonical U44 and another with N3-deprotonated U44^-^. In each set, we performed simulations with different ions in the M_A,Mg_, M_B,Mn_, and M_C_ binding sites so that the M_A,Mg_ and M_C_ binding sites were occupied by either a Mg^2+^ or K^+^ ion, while M_B,Mn_ involved either a Mn^2+^ or K^+^ ion. This strategy yielded eight combinations of ion types in these three binding sites for each U44 protonation state (**Supplementary Table 1**).

Clear rearrangements of the ion binding sites were observed in all eight simulations with the canonical form of U44. We even observed complete ejection of the canonical U44 from the ion binding site and loss of its first-shell contacts to the ions in three out of eight simulations, including the simulation containing three divalent ions (two Mg^2+^ ions in the M_A,Mg_ and M_C_ sites and one Mn^2+^ ion in the M_B,Mn_ site; Figures S10 and S11). In contrast, the simulations containing an N3-deprotonated U44^-^ revealed rather modest changes of the ion binding site arrangement and the U44^-^ always kept at least some inner-shell contacts of the crystal structure (Figures S12 and S13). Nonetheless, despite the deprotonation of the U44^-^, none of these simulations was able to completely reproduce all inner-shell contacts observed in the crystal structure. We hypothesize that the modest rearrangements are caused by the fact that we are using Mg^2+^ and Mn^2+^ (or monovalent K^+^) ions instead of Cd^2+^ ions. Note that the large Cd^2+^ ions are generally more prone to tolerate heptacoordination and nitrogen ligands than the smaller Mn^2+^ and Mg^2+^. Therefore, we suggest that the unusual arrangement of the binding site reported in chain A of the 6CB3 crystal structure is most likely induced by the presence of Cd^2+^ ion and requires an N3-deprotonated uracil U44.

**Supplementary Note 4 Stacking pattern of L3**

Most of the nucleobases from L3 loop form continuous stacking pattern (**Supplementary Figure 7 D,E** and **Supplementary** **Figure** **19**). This segment is further stacked by its 5'-end on adenine A10 (A9 according to numbering of *L. lactis*) from L1 loop, which forms type I A-minor interaction with G66=C44 (G61=C37 according to numbering of *L. lactis*) base pair. This A-minor interaction together with part of the stacking pattern formed by the A10(A9) adenine and two nucleotides at 5'-end of L3 loop (i.e., A10|A46|C47 and A9|U39|C40 in simulation of structure from *X. oryzae* and *L. lactis*, respectively) represent rather rigid part of the stacking pattern, which was found to be stable in all ionic conditions (**Supplementary Figures 15-19** and **Supplementary Table 2**). In addition to the A-minor interaction making tertiary contact between L1 and L3 loops, the above-mentioned A10|A46|C47 (A9|U39|C40) part of the stacking pattern is stabilized also by other tertiary interactions such as hydrogen bonding of C47 (C40 according to numbering of *L. lactis*) cytosine with riboses of C53 and A45 (G46 and G38 in *L. lactis*) of the P3.2 stem (see Figures S19 and S20 for evolution of all tertiary contacts between A10|A46|C47 (A9|U39|C40) part of the stacking pattern and its structural environment). The insensitivity of this region to types of ions in M_A_ and M_B_ sites suggests that the L1-L3 loop tertiary contact mediated by this pattern might be formed in all ionic conditions even if M_A_ and M_B_ sites are not yet properly formed and occupied by the corresponding divalent ions.

The rest of stacking pattern of L3 loop (i.e., A48|U52|C51|A50 and A41|C45|U44|U43 in *X. oryzae* and *L. lactis* structures, respectively) showed different dynamics depending mostly on the type of ion in M_B_ site (**Supplementary Figures 15-19** and **Supplementary Table 2**). The presence of Mn^2+^ ion in M_B_ significantly stabilized native stacking pattern in L3 loop, while this pattern was destabilized in simulations where the Mn^2+^ ion was replaced by K^+^. Surprisingly, when both divalent ions were replaced by K^+^ ions, the stability of L3 loop stacking pattern was higher than in case where only Mn^2+^ ion in M_B_ site was replaced by monovalent while M_A_ was occupied by Mg^2+^ ion, though still less stable than in simulations having also M_B_ site occupied by Mn^2+^. In order to explain this observation, we hypothesize that stacking pattern of L3 loop represents inherently quite stable conformation of this loop with life-times, when formed, at least on microsecond time scale. However, when M_A_ binding site is occupied by Mg^2+^, unlike K^+^, it enforces proper positioning of A46 and U52 (U39 and C45 according *L. lactis* numbering) phosphates which leads to destabilization of the stacking pattern of L3. This stacking-structure-destabilization effect of Mg^2+^ can be overcompensated only by binding of Mn^2+^ ion into the M_B_ binding site.

**Supplementary Note 5 Structural dynamics of SRL-like conformation of L1 in different structural contexts**

Besides structures of the whole aptamer which all remained in docked state in our simulations, we also performed simulations of the structures consisting only of P1.1, P1.2 and L1 (based on *X. oryzae* – Conformers 1 and 2 and *L. lactis* crystal structures, see **Supplementary Table 2**). Such truncated structure obviously lacks all tertiary contacts to L3 loop and P3 stem and thus its dynamics should correspond to the dynamics of this particular part in undocked state.

The base-pairing (**Supplementary Figure 22**) as well as backbone conformation were monitored and compared to the corresponding values observed in the simulations of the complete aptamer, i.e., in the docked state. Note that the overall conformation of L1 loop as well as base pairing in P1.1 stem (with exception of terminal A-U base pairs of the P1.1 stem in simulation of *L. lactis* aptamer which revealed base pair fraying) were entirely stable in the simulations of complete aptamers. The most significant structural changes were observed in simulation of the isolated P1.2|L1|P1.1 structure based on Conformer 2 of the *X. oryzae* structure. The disruption of G9(N1)…G93(N7) H-bond was followed by reconformation of G8-A94 from *trans-*Sugar Edge/Hoogsteen into *cis-*Watson Crick base pairing and loss of the S-turn conformation of the sugar-phosphate backbone of G8-A10. Interestingly, the simulation of the same structural motif derived from the Conformer 1 of *X. oryzae* crystal structure showed less pronounced changes, in particular, the G9(N1)…G93(N7) H bond was broken and recreated several times. Finally, in case of the simulation based on *L. lactis* structure, the L1 loop resembled conformation observed in the complete aptamer; however, we observed rather significant loss of pairing in P1.1 stem. This may be due to a weaker P1.1 in *L. lactis*, which is shorter by one base pair and has three A-U base pairs capping the stem, as opposed to the two G-C base pairs in the *X. oryzae* structure.

In summary, the data indicate that when A10 is sequestered in the A-minor interaction, the P1.2|L1|P1.1 segment adopts a conformation resembling the topology of a canonical sarcin-ricin loop. Compared to the sarcin-ricin loop consensus sequence, the A10 nt is inserted between its GpU platform and the flexible region. In addition, the trans-Hoogsteen/Hoogsteen base pair in the flexible region adjacent to the GpU platform is replaced by a trans-Watson-Crick/Hoogsteen pair. When the A10 is not involved in the tertiary interaction, it instead destabilizes the SRL-like topology.

**Supplementary Note 6 The *yybP-ykoY* riboswitch can discriminate between similar transition metal ions**

The selectivity of the *Xory* riboswitch for Mn^2+^ over Mg^2+^ arises in part from the inner-sphere contact with A48(N7), suggesting that other soft transition metal ions may also be recognized^7^. To test this hypothesis, we probed the effects of different divalent metal ions on the conformation of the riboswitch, at 0.1 mM concentration alone or in the presence of 1 mM Mg^2+^. FRET histograms showed that out of all the different metal ions tested, Cd^2+^ is most effective in promoting docked conformations (**Fig. 5b, Supplementary Fig. 26**). In the presence of 1 mM Mg^2+^, addition of 0.1 mM Cd^2+^ resulted in ~65 % of the high-FRET docked conformation, comparable to the docked population upon addition of 0.1 mM Mn^2+^. Examination of individual smFRET traces as well as the TODP showed that this is due to a large fraction of SD traces (**Supplementary Fig. 26**), in agreement with the tight binding of Cd^2+^ to the *yybP-ykoY* riboswitch shown recently^7^. Among the other metals tested, Ni^2+^, Co^2+^, Sr^2+^ or Zn^2+^ had little effect on promoting the folded conformations of the riboswitch under these conditions. Interestingly, in the absence of Mg^2+^, while 0.1 mM Mn^2+^ alone led to the appearance of DD and SD traces with ~62 % docked population (mean FRET 0.67±0.12) (**Fig. 5c**), 0.1 mM of Ni^2+^, Co^2+^, Sr^2+^ or Zn^2+^ did not affect SU traces and Cd^2+^ had only a small effect in promoting DD traces (**Supplementary Fig. 26**). This suggests that, while Mg^2+^ and Mn^2+^ may both bind at M_A,Mg_, Cd^2+^ may be more specific to the M_B,Mn_ site. These results suggest that while the *Xory* riboswitch has some degree of plasticity in recognizing ligands, in a background of Mg^2+^, it preferentially recognizes Mn^2+^ and Cd^2+^ and can effectively discriminate against similar divalent transition metal ions.

**Supplementary Discussion**

**Comparison of Xory Mn^2+^ bound structures to Cd^2+^ bound structures**

With respect to the recent finding that Cd^2+^ can also bind to the *yybP-ykoY* riboswitch^7^, our smFRET results and unpublished data from a *yybP-ykoY* and Broccoli-based fluorescent sensor agree with this conclusion. However, our structure (at 2.96 Å resolution) and previous ones with Mn^2+^ cannot provide strong evidence for or against their intriguing argument for heptacoordination as the mechanism of spescificity for Mn^2+^. While Cd^2+^ is heptacoordinated in the Llac-MntP structure (PDB ID 6CC3), with water as its seventh ligand, it is not clear how that water could enable a mechanism of specificity for Mn^2+^ against other metals. Further, in the highest-resolution structure (6CB3), Cd^2+^ at M_B_ was found to be hexacoordinate. The Llac-alx structure (6CC1) is also modelled as bound in a heptacoordinate fashion to M_B_, with the seventh ligand here coming from a second phosphoryl oxygen from the same phosphate of U44. However, this structure is similar to all of our Mn^2+^-bound structures, in that the resolution is not high enough to show the subtle difference in orientation of a phosphate that would be required to distinguish hexa- from heptacoordination. As for the M_A_ site, our Cd^2+^-only smFRET data suggest that M_A_ probably prefers either Mg or Mn over Cd^2+^. Upon inspection of their data, M_A_ does not appear clearly heptacoordinate in any of their structures. It is actually octacoordinated in the *Llac* structure with Cd/Mg/Ba (PDB 6CB3) and another claim (Llac-MntP) relies on placed waters not seen in the electron density. A very high-resolution structure of a *yybP-ykoY* riboswitch with Mn^2+^ is required to address this interesting question. In any case, taken together with our structures, these prior structures agree with the finding of flexibility in the Mn-binding area, even when the riboswitch ligand is bound. It is a separate question whether Cd^2+^ is a biologically relevant ligand, as it is typically considered toxic and xenobiotic for most organisms, and its high-affinity binding to enzymes at sites of other metals is generally considered aberrant^8^.

**Supplementary References**

1. Bottaro, S., Di Palma, F. & Bussi, G. The role of nucleobase interactions in RNA structure and dynamics. *Nucleic Acids Res.* **42**, 13306-14 (2014).

2. Kuhrova, P., Banas, P., Best, R.B., Sponer, J. & Otyepka, M. Computer Folding of RNA Tetraloops? Are We There Yet? *J. Chem. Theory Comput.* **9**, 2115-25 (2013).

3. Sponer, J.E. et al. Theoretical studies on the intermolecular interactions of potentially primordial base-pair analogues. *Chemistry (Easton)* **16**, 3057-65 (2010).

4. Menger, M., Eckstein, F. & Porschke, D. Dynamics of the RNA hairpin GNRA tetraloop. *Biochemistry* **39**, 4500-7 (2000).

5. Zhao, L. & Xia, T. Direct revelation of multiple conformations in RNA by femtosecond dynamics. *J. Am. Chem. Soc.* **129**, 4118-9 (2007).

6. Kuhrova, P. et al. Computer Folding of RNA Tetraloops: Identification of Key Force Field Deficiencies. *J. Chem. Theory Comput.* **12**, 4534-48 (2016).

7. Bachas, S.T. & Ferre-D'Amare, A.R. Convergent Use of Heptacoordination for Cation Selectivity by RNA and Protein Metalloregulators. *Cell Chem Biol* **25**, 962-973 e5 (2018).

8. Begg, S.L. et al. Dysregulation of transition metal ion homeostasis is the molecular basis for cadmium toxicity in Streptococcus pneumoniae. *Nat Commun* **6**, 6418 (2015).
