# Supplementary material for: Local-to-global signal transduction at the core of a Mn2+ sensing riboswitch
Source: Nat Commun. 2019 Sep 20;10:4304. doi: 10.1038/s41467-019-12230-5 (PMC6754395; doi:10.1038/s41467-019-12230-5)
Supplement: Supplementary file 3 — Reporting Summary [file 41467_2019_12230_MOESM3_ESM.pdf]

nature research

Corresponding author(s): Nils G Walter, Ailong Ke

Last updated by author(s): Aug 12, 2019

Reporting Summary

Nature Research wishes to improve the reproducibility of the work that we publish. This form provides structure for consistency and transparency in reporting. For further information on Nature Research policies, see Authors & Referees and the Editorial Policy Checklist .

Statistics

For all statistical analyses, confirm that the following items are present in the figure legend, table legend, main text, or Methods section.

n/a	Confirmed	
☐	☒	The exact sample size (n) for each experimental group/condition, given as a discrete number and unit of measurement
☐	☒	A statement on whether measurements were taken from distinct samples or whether the same sample was measured repeatedly
☒	☐	The statistical test(s) used AND whether they are one- or two-sided<br>*Only common tests should be described solely by name; describe more complex techniques in the Methods section.*
☒	☐	A description of all covariates tested
☒	☐	A description of any assumptions or corrections, such as tests of normality and adjustment for multiple comparisons
☐	☒	A full description of the statistical parameters including central tendency (e.g. means) or other basic estimates (e.g. regression coefficient) AND variation (e.g. standard deviation) or associated estimates of uncertainty (e.g. confidence intervals)
☒	☐	For null hypothesis testing, the test statistic (e.g. F, t, r) with confidence intervals, effect sizes, degrees of freedom and P value noted<br>*Give P values as exact values whenever suitable.*
☒	☐	For Bayesian analysis, information on the choice of priors and Markov chain Monte Carlo settings
☒	☐	For hierarchical and complex designs, identification of the appropriate level for tests and full reporting of outcomes
☒	☐	Estimates of effect sizes (e.g. Cohen's d, Pearson's r), indicating how they were calculated

Our web collection on statistics for biologists contains articles on many of the points above.

Software and code

Policy information about availability of computer code

Data collection	AMBER 14, Matlab 2012b
Data analysis	XDS, Phenix, Coot, Pymol, AMBER 14, VMD, baRNAba, Matlab R2012b, IDL (Research Systems), QuB, MicroCal Origin Pro 8.5

For manuscripts utilizing custom algorithms or software that are central to the research but not yet described in published literature, software must be made available to editors/reviewers. We strongly encourage code deposition in a community repository (e.g. GitHub). See the Nature Research guidelines for submitting code & software for further information.

Data

Policy information about availability of data

All manuscripts must include a data availability statement. This statement should provide the following information, where applicable:

- Accession codes, unique identifiers, or web links for publicly available datasets
- A list of figures that have associated raw data
- A description of any restrictions on data availability

The data supporting the findings of this study are available from the corresponding authors upon reasonable request. Atomic coordinates and structure factors for the reported crystal structure have been deposited with the Protein Data Bank under accession number 6N2V. The source data underlying Figs 4e, h and 7c, e and Supplementary Figs 1a and 5d are provided as a Source Data file.

Field-specific reporting

Please select the one below that is the best fit for your research. If you are not sure, read the appropriate sections before making your selection.

Life sciences study design

All studies must disclose on these points even when the disclosure is negative.

Sample size	Sample size for smFRET is number of molecules under each condition. Generally, at least 100 molecules are analyzed in each condition for reproducibility and to obtain better estimates for the parameters of interest.
Data exclusions	No data were excluded from the analyses.
Replication	All smFRET experiments were repeated at least two times to verify reproducibility and the replicative experiments were successful. Representative measurement for each condition tested is shown in the paper.
Randomization	No Randomization was done in the analysis, as this is not relevant for the study.
Blinding	Investigators were not blinded to collection and analysis of experiments. The data reported for X-ray structure, MD simulations and smFRET were not subjective but based on quantit

Reporting for specific materials, systems and methods

We require information from authors about some types of materials, experimental systems and methods used in many studies. Here, indicate whether each material, system or method listed is relevant to your study. If you are not sure if a list item applies to your research, read the appropriate section before selecting a response.

Materials & experimental systems

n/a	Involved in the study
☒	☐ Antibodies
☒	☐ Eukaryotic cell lines
☒	☐ Palaeontology
☒	☐ Animals and other organisms
☒	☐ Human research participants
☒	☐ Clinical data

Methods

n/a	Involved in the study
☒	☐ ChIP-seq
☒	☐ Flow cytometry
☒	☐ MRI-based neuroimaging
