# Supplementary material for: Local-to-global signal transduction at the core of a Mn2+ sensing riboswitch
Source: Nat Commun. 2019 Sep 20;10:4304. doi: 10.1038/s41467-019-12230-5 (PMC6754395; doi:10.1038/s41467-019-12230-5)
Supplement: Supplementary file 2 — Peer Review [file 41467_2019_12230_MOESM2_ESM.pdf]

Editorial Note: This manuscript has been previously reviewed at another journal that is not operating a transparent peer review scheme. This document only contains reviewer comments and rebuttal letters for versions considered at *Nature Communications*.

REVIEWERS' COMMENTS:

Reviewer #1 (Remarks to the Author):

The revised manuscript by Suddala et al. entitled "Local-to-global signal transduction at the core of the Mn2+ sensing riboswitch" adequately addresses reviewers' concerns. The improved manuscript includes technical corrections and additional crystallographic analyses, and includes new "SiM-KARTS" assay data that address the accessibility of the P1.1 helix involved in terminator-antiterminator switching. Overall, the authors fairly address criticisms in their rebuttal.

Given apparent differences in Mn2+ sensing riboswitches from X.ory and L.lac newly addressed in the discussion of the manuscript, which are interesting from a divergent or convergent evolutionary perspective and possibly different physiological needs of the organisms, the precise mechanistic and functional properties of Mn2+ sensing riboswitches do not appear to be universal. Therefore, it would seem appropriate that the title of the manuscript be revised to state "a Mn2+ sensing riboswitch" or "the X. oryzae Mn2+ sensing riboswitch" to be precise.

Some minor word choices:
1. Line 103, "hence providing a nice reference point", consider changing "nice" to something more descriptive like "appropriate", "comparable", or "common".
2. Line 108, "flips out", consider "extrudes". Also Line 610, "single unconserved U flip-out", consider "unconserved U single nucleotide bulge".
3. Line 357-358, "These results thus provide a direct evidence yet for the prevailing mechanistic hypothesis of riboswitching" is probably meant to say "provide direct evidence for".

Signed,
Garrett Soukup

Reviewer #2 (Remarks to the Author):

I was asked to comment explicitly on the smFRET data analysis in this manuscript, which I note has previously been out to review. During this initial review process reviewer 2 noted some reasonable concerns over the TODP plots presented by the authors, I will limit my comments here to this specific issue.

The TODP plots are a non-standard analysis, which are easily confused with the more common TDP plots. This is what confused the initial reviewer 2, and in my opinion would continue to confuse even expert readers of the revised manuscript.

I do not feel the authors have sufficiently explained this analysis method in their revised manuscript (even though they provide a reference). The analysis itself appears to be sound, in trying to represent the percentages of different types of traces (rather than different types of transitions), however, this subtle but crucial distinction is not sufficiently well explained in neither the main text, the figure legends, the methods nor the supp. material (I could not find any additional explanation in the methods in the new version!).

I would recommend this be addressed before publication.

Reviewer #3 (Remarks to the Author):

The authors have addressed my concerns. I still noted one typo (line 118, "discernable") and some left-over use of "metal" instead of "metal ion" (line 157, inter alia). Finally, if the authors plan to stay in the nucleic acids field, they would be well served to turn off the "fancy quotes" option in their software, so that they can write 5-prime and 3-prime, as opposed to their current "5-fancy quote" and "3-fancy-quote".

REVIEWERS' COMMENTS:

Reviewer #1 (Remarks to the Author):

The revised manuscript by Suddala et al. entitled "Local-to-global signal transduction at the core of the Mn2+ sensing riboswitch" adequately addresses reviewers' concerns. The improved manuscript includes technical corrections and additional crystallographic analyses, and includes new "SiM-KARTS" assay data that address the accessibility of the P1.1 helix involved in terminator-antiterminator switching. Overall, the authors fairly address criticisms in their rebuttal.

Given apparent differences in Mn2+ sensing riboswitches from X.ory and L.lac newly addressed in the discussion of the manuscript, which are interesting from a divergent or convergent evolutionary perspective and possibly different physiological needs of the organisms, the precise mechanistic and functional properties of Mn2+ sensing riboswitches do not appear to be universal. Therefore, it would seem appropriate that the title of the manuscript be revised to state "a Mn2+ sensing riboswitch" or "the X. oryzae Mn2+ sensing riboswitch" to be precise.

Some minor word choices:
1. Line 103, "hence providing a nice reference point", consider changing "nice" to something more descriptive like "appropriate", "comparable", or "common".
2. Line 108, "flips out", consider "extrudes". Also Line 610, "single unconserved U flip-out", consider "unconserved U single nucleotide bulge".
3. Line 357-358, "These results thus provide a direct evidence yet for the prevailing mechanistic hypothesis of riboswitching" is probably meant to say "provide direct evidence for".

Signed,
Garrett Soukup

We thank the reviewer Dr. Garrett Soukup for his nice words on the manuscript. We have changed the title of the manuscript to state "a Mn2+ sending riboswitch" as per the suggestion. In addition, we have included their suggested minor word changes.

Reviewer #2 (Remarks to the Author):

I was asked to comment explicitly on the smFRET data analysis in this manuscript, which I note has previously been out to review. During this initial review process reviewer 2 noted some reasonable concerns over the TODP plots presented by the authors, I will limit my comments here to this specific issue.

The TODP plots are a non-standard analysis, which are easily confused with the more common TDP plots. This is what confused the initial reviewer 2, and in my opinion would continue to confuse even expert readers of the revised manuscript.

I do not feel the authors have sufficiently explained this analysis method in their revised manuscript (even though they provide a reference). The analysis itself appears to be sound, in trying to represent the percentages of different types of traces (rather than different types of transitions), however, this subtle but crucial distinction is not sufficiently well explained in neither the main text, the figure legends, the methods nor the supp. material (I could not find any additional explanation in the methods in the new version!).

I would recommend this be addressed before publication.

We agree with the reviewer that some details about the (non-standard) TODPs were missing in the previous draft. We have now included a more detailed description of the TODPs in the Methods section and refer the reader to this segment when talking about TODPs in the Results section. We have also added two new references on TODPs for the benefit of the readers. We thank the reviewer for their advice.

Reviewer #3 (Remarks to the Author):

The authors have addressed my concerns. I still noted one typo (line 118, "discernable") and some left-over use of "metal" instead of "metal ion" (line 157, inter alia). Finally, if the authors plan to stay in the nucleic acids field, they would be well served to turn off the "fancy quotes" option in their software, so that they can write 5-prime and 3-prime, as opposed to their current "5-fancy quote" and "3-fancy-quote".

We have changed all instances of “metal” into “metal ion” along with fixing the typographical error. As per the suggestion, we have now changed all single "smart quotes" to straight quotes and thank the reviewer for their suggestions.
